# LUBAC enables tumor-promoting LTβ receptor signaling by activating canonical NF-κB

**DOI:** 10.1038/s41418-024-01355-w

**Published:** 2024-08-30

**Authors:** Yu-Guang Chen, Eva Rieser, Amandeep Bhamra, Silvia Surinova, Peter Kreuzaler, Meng-Hsing Ho, Wen-Chiuan Tsai, Nieves Peltzer, Diego de Miguel, Henning Walczak

**Affiliations:** 1 Centre for Cell Death, Cancer, and Inflammation (CCCI), UCL Cancer Institute, University College London, London, UK; 2 Division of Hematology/Oncology, Department of Internal Medicine, Tri-Service General Hospital, National Defense Medical Center, Taipei, Taiwan; 3 Institute of Biochemistry I, Medical Faculty, University of Cologne, Cologne, Germany; 4 CECAD Research Centre, University of Cologne, Cologne, Germany; 5 Proteomics Research Translational Technology Platform, UCL Ciancer Institute and Cancer Research UK UCL Centre, University College London (UCL), London, UK; 6 Division of General Surgery, Department of Surgery, Tri-Service General Hospital, National Defense Medical Center, Taipei, Taiwan; 7 Department of Pathology, Tri-Service General Hospital, National Defense Medical Center, Taipei, Taiwan; 8 Department of Translational Genomics and Center for Molecular Medicine Cologne (CMMC), University of Cologne, Medical Faculty, Cologne, Germany; 9 Department of Genome Editing, University of Stuttgart, Stuttgart, Germany; 10 Aragon Health Research Institute (IIS Aragon), Biomedical Research Centre of Aragon (CIBA), Zaragoza, Spain

**Keywords:** Proteomics, Cell death and immune response

## Abstract

Lymphotoxin β receptor (LTβR), a member of the TNF receptor superfamily (TNFR-SF), is essential for development and maturation of lymphoid organs. In addition, LTβR activation promotes carcinogenesis by inducing a proinflammatory secretome. Yet, we currently lack a detailed understanding of LTβR signaling. In this study we discovered the linear ubiquitin chain assembly complex (LUBAC) as a previously unrecognized and functionally crucial component of the native LTβR signaling complex (LTβR-SC). Mechanistically, LUBAC-generated linear ubiquitin chains enable recruitment of NEMO, OPTN and A20 to the LTβR-SC, where they act coordinately to regulate the balance between canonical and non-canonical NF-κB pathways. Thus, different from death receptor signaling, where LUBAC prevents inflammation through inhibition of cell death, in LTβR signaling LUBAC is required for inflammatory signaling by enabling canonical and interfering with non-canonical NF-κB activation. This results in a LUBAC-dependent LTβR-driven inflammatory, protumorigenic secretome. Intriguingly, in liver cancer patients with high LTβR expression, high expression of LUBAC correlates with poor prognosis, providing clinical relevance for LUBAC-mediated inflammatory LTβR signaling.

## Introduction

Lymphotoxin-β (LT-β) receptor (LTβR) is a member of the tumor necrosis factor (TNF) receptor (TNFR) superfamily (SF) (TNFRSF) and is indispensable for the maintenance of immune system homeostasis [1–4]. Engagement of LTβR by either of its two TNFSF member ligands, heterotrimeric LTɑ1β2 or homotrimeric LIGHT, can trigger activation of both, canonical and noncanonical NF-κB signaling, resulting in the secretion of a distinct set of chemo- and cytokines [5–8]. Certain homeostatic chemokines, namely CXCL13, CCL19 and CCL21, which are mainly driven by LTβR-mediated activation of non-canonical NF-κB, were demonstrated to be crucial for immune cell homeostasis and maturation of lymphoid organs [9–11]. In mice in which LTβR signaling was blocked or LTβR was genetically ablated in endothelial cells, an impaired development of lymph nodes (LNs), accompanied by reduced expression of CXCL13, CCL19 and CCL21, was observed [12]. Intriguingly, mice lacking LTβR in CXCL13-expressing cells presented with similar phenotypic abnormalities in terms of decreased cellularity of LNs and ablated formation of B-cell follicles [13]. LTβR-mediated chemokine secretion by thymic endothelial cells is, in turn, required for proper T cell selection in the thymus [14–16], and LTβR-mediated chemokine secretion by lymphatic endothelial cells is responsible for the homing of immune cells from the bone marrow to different target organs [17, 18].

Whilst the role of LTβR in immune regulation and inflammation has been well established, less is known about its implication in cancer, especially in terms of the relative contribution of classical versus alternative NF-κB activation. In patients with non-alcoholic steatohepatitis (NASH)-derived hepatocellular carcinoma (HCC), renal cell carcinoma (RCC) and head and neck squamous cell carcinoma (HNSCC), increased expression of LTβR correlated with disease progression and a worsened prognosis [19–21]. Mechanistically, LTβR-derived signaling cascades and subsequent chemo-/cytokine production are required for the recruitment of different immune cells which cooperatively promote tumorigenesis in both, AKT/β-catenin-derived intrahepatic cholangiocarcinoma and in LTβ-hepatocyte-specific overexpression-derived HCC [8, 22–24]. Intriguingly, in the latter model the incidence of HCC was decreased by specific *Ikkβ* deletion in hepatocytes and by inhibition of LTβR signaling using soluble LTβR-Fc, but not upon deletion of TNFR1 or TNFR2 [19]. Furthermore, conditional deletion of LTβR in hepatocytes or whole-body ablation of LIGHT abrogated hepatocarcinogenesis by inducing a substantial decrease in proinflammatory chemo-/cytokine production and liver infiltration by CD8 and natural killer T (NKT) cells in a long-term choline-deficient high-fat diet-driven HCC model in mice [25]. On the other hand, activation of LTβR in HNSCC was shown to induce activation of both canonical and non-canonical NF-κB pathways, encompassing with secretion of proinflammatory cytokines, both in patient samples and in cell lines [26]. This study identified a unique expression pattern of genes involved in NF-κB pathways, inflammatory cytokine production and metastasis upon LTβR activation. Although it described a potential crosstalk between canonical and non-canonical NF-κB pathways, their respective contribution to the observed pro-inflammatory gene signature remained unresolved [26]. Together, these studies indicate that LTβR activation induces the production of pro-inflammatory chemo- and cytokines and that this promotes carcinogenesis and disease progression by recruiting pro-tumorigenic immune cells to the tumor tissue. This identification of a crucial role of LTβR signaling in tumorigenesis calls for a thorough understanding of the biochemical mechanisms underlying LTβR-mediated signaling and its functional output, as it may provide insight on previously unrecognized therapeutic vulnerabilities, particularly for cancer patients with high LTβR expression.

Currently, LTβR signaling is known to be regulated by ubiquitination and phosphorylation of different proteins and that this, together, enables the induction of chemo-/cytokine expression [4, 8]. Similar to other TNFRSF members, stimulation by either of its cognate ligands, LIGHT or LTɑ1β2, triggers LTβR multimerization and recruitment of different members of the TRAF protein family to the intracellular domains of the crosslinked receptors. In the case of LTβR, these are TRAF2, TRAF3 and TRAF5 [27, 28]. These adaptor proteins mediate the recruitment of the ubiquitin E3s cIAP1 and cIAP2 which catalyze the formation of K48- and K63-linked ubiquitin chains on different substrates within the LTβR signaling complex (LTβR-SC). K48-linked ubiquitination of TRAF3 induces its proteasomal degradation, resulting in the stabilization of NIK and activation of the non-canonical NF-κB pathway by phosphorylation of p100 [29]. On the other hand, K63-linked ubiquitination of the NF-κB essential modulator (NEMO/IKKɣ) stabilized the inhibitor of NF-κB kinase (IKK) complex (composed of IKKα, IKKβ and NEMO/IKKɣ) and enabled its recruitment to the LTβR-SC, resulting in activation of canonical NF-κB signaling [5]. Despite the importance of LTβR signaling in immune homeostasis and tumorigenesis, the precise molecular mechanisms that regulate LTβR signaling, including activation of the canonical and non-canonical NF-κB signaling pathways and how they respectively impact the LTβR-derived tumor-promoting chemo-/cytokine production, remain largely unknown.

We therefore undertook an unbiased proteomic analysis of LTβR signaling and functionally examined the respective contributions of its different signaling arms, especially with regards to the tumor-promoting chemo-/cytokine production. This revealed the linear ubiquitin chain assembly complex (LUBAC) as a previously unrecognized component of the native LTβR-SC that is required for tumorigenic inflammatory LTβR signaling. Intriguingly, our results show that LUBAC regulates the respective signaling outputs of LTβR and DRs, such as TNFR1, in decisively distinct manners: whilst linear ubiquitination prevents cell death-driven inflammation downstream of DRs [30–36], LUBAC enables, and is indeed required for, LTβR-induced inflammatory signaling by favoring canonical over non-canonical NF-κB, importantly, independently of cell death induction. Analysis of cancer patient-derived RNA-sequencing data from public databases, revealed a strong correlation between decreased overall survival and the combination of high expression of both, HOIP and LTβR in HCC patients. Together, we here identify LUBAC as a previously unrecognized crucial factor that determines the functional signaling output of LTβR activation, which could be exploited for cancer patient stratification and new therapeutic approaches.

## Results

### An unbiased functional and proteomic analysis unveils previously unrecognized components of the LTβR-SC

To gain a better understanding of LTβR signaling in cancer cells, we first performed mechanistic and functional analyses of LTβR activation in several cancer cell lines representing different tissues of origin. For our analyses, we chose cell lines that abundantly express LTβR on their surface whilst lacking the expression of HVEM, the other known receptor for LIGHT (Fig. S1A) [8]. We first studied the signaling events triggered by LTβR activation upon LIGHT stimulation by Western blotting. Treatment with LIGHT resulted in robust activation of canonical NF-κB within 15 min as determined by phosphorylation of IκBα, IKKα/β and p65 (Fig. S1B). At later times, activation of non-canonical NF-κB was also observed, as expression of NIK and processing of p100/p52 were increased and the levels of TRAF3 and cIAP1 decreased over time (Fig. S1B).

**Fig. 1:**
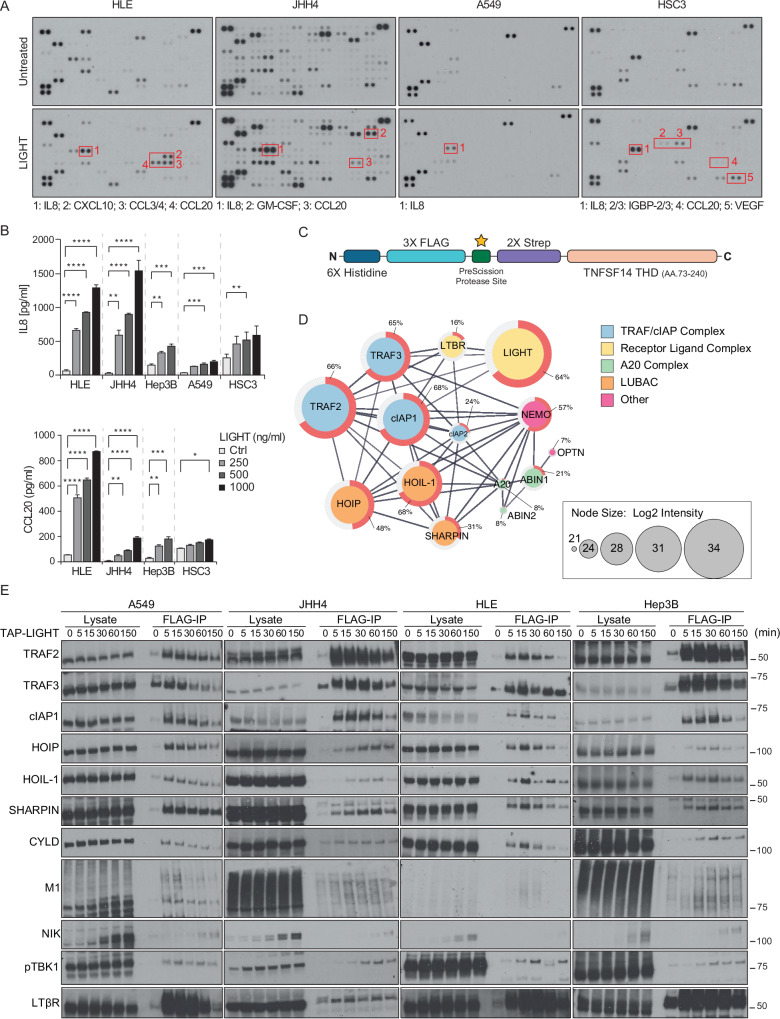
Unbiased functional and proteomic analysis reveals LUBAC as a new component of the native LTβR-SC. **A** The indicated four cell lines were stimulated with 500 ng/ml of TAP-LIGHT overnight or left untreated, and the correspondent supernatants were collected and subjected to a secretome analysis by using a human cytokine array. **B** The indicated cell lines were stimulated with increasing concentrations TAP-LIGHT for 24 h, and the concentration of IL-8 or CCL20 was determined by ELISA. Error bars represent the mean ± SEM of three independent experiments. Statistical significance was assessed by two-tailed Student’s t-test. *p < 0.05, **p < 0.01, ***p < 0.001, ****p < 0.0001. **C** Schematic representation of TAP-LIGHT. **D** Hep3B cells were stimulated with TAP-LIGHT (2000 ng/ml) followed by total protein extraction. The native LTβR-SC was purified via anti-FLAG M2 beads, and analyzed by mass spectrometry (*n* = 2). A STRING functional connectivity network was generated for the LTβR-SC. Relative protein abundances are represented by the node size and sequence coverage is depicted on the outer ring in red. **E** Analysis of native LTβR-signaling complex (SC) in four different cancer cell lines. Cells were stimulated with TAP-LIGHT (2000 ng/ml) for the indicated times, LTβR-SC was immunoprecipitated as in (**D**) and analyzed by Western blotting. Representative results of at least two independent replicates are shown.

To study the secretome profile induced by LIGHT-induced stimulation of LTβR, we performed chemo-/cytokine profiling. Stimulation with LIGHT for 24 h induced a distinct pattern of chemo-/cytokine secretion characterized by the presence of pro-inflammatory cytokines such as IL-8 and CCL20 (Fig. 1A). IL-8 and CCL20 were induced by LTβR stimulation in two different liver cancer cell lines (HLE and JHH4) and in one HNSCC line (HSC3). IL-8 was also increased in A549 lung adenocarcinoma cells upon stimulation with LIGHT. Increased secretion of CCL20 and IL-8 upon LTβR activation was validated by ELISA (Fig. 1B). To test for a possible effect of an autocrine TNF loop induced by LTβR stimulation, we employed the soluble TNF inhibitor Etanercept (Enbrel®; TNFR2-Fc) and found that, whereas this effectively blocked TNF-induced IL-8 secretion, it did not interfere with LIGHT-mediated IL-8 secretion (Fig. S1C). Further confirming the specificity of this effect on the LTβR signaling axis, stimulation of LTβR-proficient cells with the other LTβR ligand, LTα1β2, also induced secretion of IL-8 and CCL20 (Fig. S1D). Moreover, secretion of IL-8 and CCL20 was completely abrogated in LTβR-deficient cells generated by CRISPR/Cas9 (Fig. S1E). Surprisingly, despite a clear activation of the non-canonical NF-κB pathway in the cancer cells tested, they did not secrete the chemokines CXCL12, CXCL13, CCL19 and CCL21, known to be induced as a consequence of activation of this signaling pathway upon LTβR stimulation of other cell types [12]. To further confirm that the secretion of IL-8 and CCL20 following LTβR stimulation was dependent on the canonical NF-κB pathway, we used small molecule inhibitors to block key components of both, the canonical and non-canonical NF-κB pathways. As shown in Fig. S1F, whereas specific inhibition of TAK1 or IKKα/β with 5z-7-oxozeaenol or TPCA, respectively, prevented LIGHT-induced secretion of IL-8 and CCL20, inhibition of NIK with B022 failed to do so, confirming that LTβR-induced secretion of IL-8 and CCL20 is mediated by canonical NF-κB activation.

We next set out to explore the composition of the native LTβR-SC. To do so, we took an unbiased approach based on mass spectrometry, similar to what we previously employed to determine the composition of other native receptor-associated SCs, including the CD40-SC, the TNFR1-SC and the TRAILR-SC [30, 31, 37]. We first produced recombinant LIGHT with an N-terminal modified tandem affinity purification (moTAP) tag (Fig. 1C). Following stimulation of Hep3b cells with this recombinant moTAP-tagged LIGHT (TAP-LIGHT), we purified the native LTβR-SC via two sequential affinity purification steps (one FLAG- and one Streptag-based) before performing liquid chromatography-coupled tandem mass spectrometry (LC-MS/MS) on the resulting purified native LTβR-SC. Apart from confirming the presence of known components of the LTβR-SC, including TRAF2, TRAF3 and cIAP1, this unbiased proteomic analysis revealed, amongst others, the linear ubiquitin chain assembly complex (LUBAC), composed of HOIL-1, HOIP and SHARPIN, as a prominent and previously unrecognized component of the native LTβR-SC (Fig. 1D and Supplementary Table 1). Apart from LUBAC, we found NEMO, A20 and OPTN, all previously reported to be capable of binding linear ubiquitin chains [38–40], as well as the A20-binding inhibitor of NF-κB activation (ABIN)1 and ABIN2, to form part of the native LTβR-SC (Fig. 1D).

Using Western blotting, we corroborated that LUBAC is recruited to the LTβR-SC in a LIGHT-stimulation-dependent manner in four different cancer cell lines (Fig. 1E). This analysis also revealed that LUBAC recruitment to the LTβR-SC occurred with kinetics similar to those of recruitment of TRAF2, TRAF3 and cIAP1. Notably, we also found TBK1 to be recruited to the LTβR-SC upon LIGHT stimulation. LUBAC is the only known ubiquitin E3 capable of generating linear (also referred to as M1-linked) ubiquitin chains de novo under physiological conditions [41, 42]. In line with this, we detected linear ubiquitination within the native LTβR-SC, implying LUBAC activity within this SC (Fig. 1E).

### Secretion of pro-inflammatory cytokines by LTβR-mediated canonical NF-κB activation is dependent on LUBAC and its activity

**Fig. 2:**
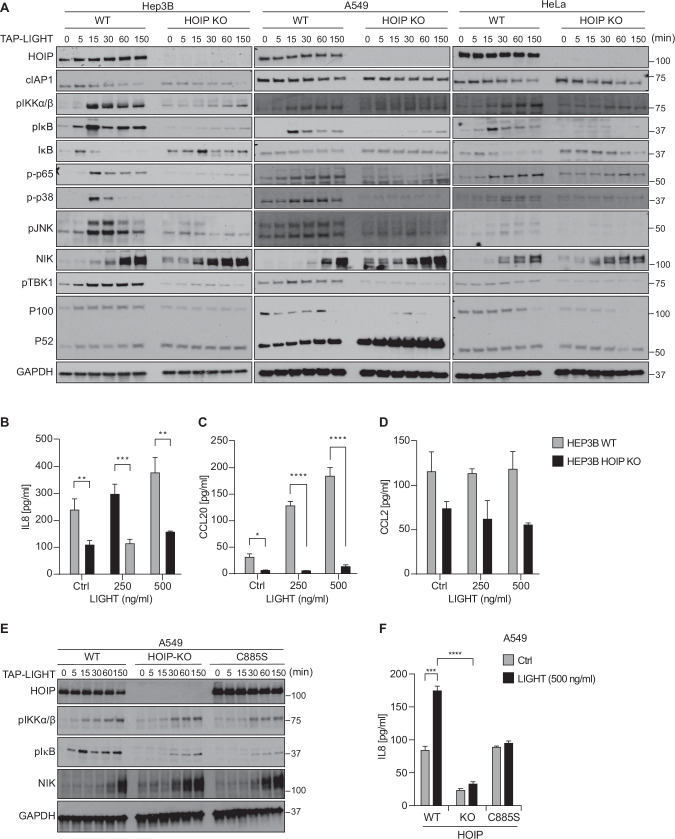
Secretion of proinflammatory cytokines by LTβR-induced canonical NF-κB is dependent on LUBAC. **A** HOIP proficient or deficient cell lines were stimulated with TAP-LIGHT (200 ng/ml) for the indicated time points followed by total protein extraction. Cell lysates were subjected to Western blot analysis and probed for the indicated proteins. Representative results of at least three independent replicates are shown. HOIP proficient or deficient Hep3B cells were stimulated overnight with the indicated concentrations of LIGHT. Supernatants were collected and levels of IL-8 (**B**), CCL20 (**C**) and CCL2 (**D**) were quantified by ELISA. Error bars represent the mean ± SEM of three independent experiments. Statistical significance was assessed by two-tailed Student’s t-test. *p < 0.05, **p < 0.01, ***p < 0.001, ****p < 0.0001. **E** A549 WT, HOIP KO or HOIP-C885S cell lines were stimulated with TAP-LIGHT (200 ng/ml) for the indicated time points followed by total protein extraction. Cell lysates were subjected to Western blot analysis and probed for the indicated proteins. Representative results of at least three independent replicates are shown. **F** A549 WT, HOIP KO or HOIP-C885S cell lines were stimulated overnight with TAP-LIGHT (200 ng/ml). Supernatants were collected and levels of IL-8 were quantified by ELISA. Error bars represent the mean ± SEM of three independent experiments. Statistical significance was assessed by two-tailed Student’s t-test. *p < 0.05, **p < 0.01, ***p < 0.001, ****p < 0.0001.

We and others previously showed that LUBAC plays a crucial role in determining the signaling output of various immune receptor SCs [30, 32, 35, 36, 43–53]. In many of these protein complexes, LUBAC plays an eminent role in gene-activatory signaling by enabling optimal activation of canonical NF-κB following activation of these receptors [42, 54–56]. Importantly, canonical NF-κB activation driven by LTβR activation in hepatocytes is responsible for the recruitment of lymphocytes and, in consequence thereof, the promotion of hepatocarcinogenesis [19]. Therefore, we sought to determine the role of LUBAC in LTβR-induced NF-κB activation in cancer cells. We first analyzed the kinetics of NF-κB activation upon stimulation with LIGHT in several HOIP-proficient as compared to HOIP-deficient cancer cell lines. Genetic deletion of HOIP severely impaired phosphorylation and activation of canonical NF-κB mediators such as IKKα/β, IκBα and p65, as well as of the MAPKs JNK and p38 and, finally, also of TBK1 (Fig. 2A). Intriguingly, however, HOIP-deficient cells showed an earlier and more robust activation of the non-canonical NF-κB pathway as shown by increased accumulation of NIK and processing of p100/p52. In line with decreased activation of the canonical NF-κB pathway observed by Western blotting, the LIGHT-induced production of IL-8 and CCL20 was drastically decreased in HOIP-deficient cells as compared to WT cells (Fig. 2B–D). Importantly, cells expressing a catalytically inactive version of HOIP (HOIP-C885S), also showed reduced canonical and increased non-canonical NF-κB signaling upon LIGHT stimulation and, consequently, impaired secretion of IL-8, similar to what we observed in HOIP-deficient cells (Fig. 2E, F). Collectively, these results show that LUBAC and its linear ubiquitin chain-forming enzymatic activity are required for the activation of canonical NF-κB and pro-inflammatory chemo-/cytokine production upon stimulation of LTβR.

### LUBAC deficiency does not sensitize to cell death upon engagement of LTβR

It was previously reported that LTβR signaling can promote cell death in certain cancer types [27, 57, 58]. This is thought to occur as a consequence of the gradual degradation of cIAP1/2 levels induced upon activation of LTβR which triggers the non-canonical NF-κB pathway. However, we could not detect a reduction in cell viability or induction of cell death upon LTβR activation among the different cancer cell lines we tested (Fig, S2A, B). We also examined whether LIGHT could promote cytotoxicity upon depletion of cIAP1/2 using a SMAC mimetic (SM) compound. However, whereas SM efficiently killed Hep3B cells in combination with TNF, when combined with LIGHT it did not (Fig, S2C, D). As we previously showed that LUBAC acts as a gatekeeper for cell death induction by TNF, FasL and TRAIL, independently of NF-κB regulation [36, 59, 60], we next examined whether LUBAC deficiency could sensitize cancer cells to death induced by LTβR activation. However, cells deficient in HOIP or its catalytic activity were not sensitized to cell death upon LIGHT stimulation (Fig. S2E, F). Collectively, these results show that the main functional outcome of LTβR activation in cancer cells is the secretion of chemo-/cytokines, that this requires the LUBAC- and linear ubiquitin-dependent activation of canonical NF-κB and that LTβR stimulation is not capable of inducing the death of these cells even in the absence of linear ubiquitination.

### LUBAC is required for the recruitment of NEMO, A20 and OPTN to the LTβR-SC

**Fig. 3:**
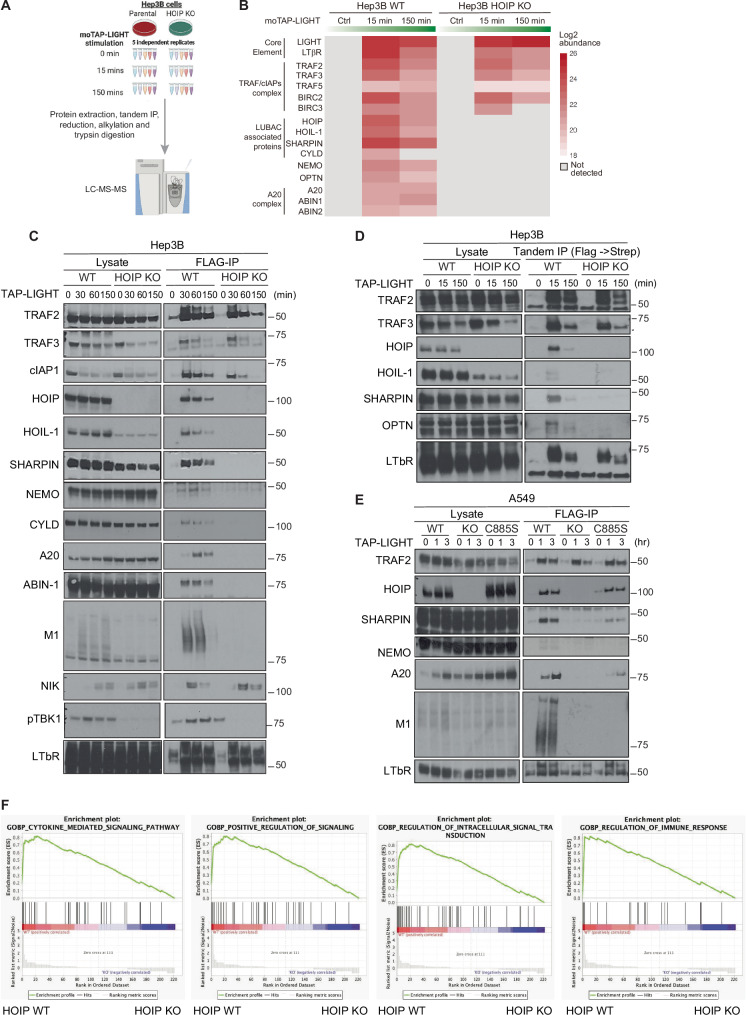
LUBAC is required for the recruitment of NEMO, A20 and OPTN to the LTβR-SC. **A** Schematic overview of the proteomic analysis of the native LTβR-SC performed in HOIP proficient and deficient Hep3B cells. **B** Heatmap represents a linear model-based of log2 protein abundances of five independent experiments. **C**, **D** HOIP proficient or deficient Hep3B cells were stimulated with TAP-LIGHT (2000 ng/ml) for the indicated times followed by total protein extraction. Native LTβR-SC was isolated by using M2-beads and analyzed by Western blotting. Representative results of at least three independent replicates are shown. **E** A549 WT, HOIP KO or HOIP-C885S were stimulated with TAP-LIGHT (2000 ng/ml) for the indicated times followed by total protein extraction. Native LTβR-SC was isolated by using M2-beads and analyzed by western blotting. Representative results of at least three independent replicates are shown. **F** Gene Set Enrichment Analysis (GSEA) comparing the results obtained by LC-MS/MS on the composition of the LTβR-SC obtained from WT vs. HOIP-deficient cells using the quantitative peptide number data obtained from five independent experiments. The thresholds for this analysis were established as follows: (1) an FDR of less than 0.25, and (2) an adjusted *P*-value of less than 0.01.

Having confirmed the functional relevance of LUBAC in LTβR-induced secretion of pro-inflammatory chemo-/cytokines via canonical NF-κB, we next sought to biochemically dissect the role of LUBAC in the formation of the LTβR-SC. To this end, we performed LC–MS/MS analysis of the LTβR-SC in both, HOIP-proficient and -deficient Hep3B cells (Fig. 3A). This analysis revealed that, whilst recruitment of TRAF2, TRAF3 and cIAP1/2 was not affected by the absence of HOIP, it completely abrogated recruitment of NEMO, OPTN, TBK1, CYLD and the A20–ABIN1–ABIN2 complex to the LTβR-SC (Fig. 3B–D and Supplementary Table 2). On the other hand, cells lacking the linear ubiquitin-generating enzymatic activity of HOIP showed an impaired recruitment of NEMO and A20, demonstrating that linear ubiquitin chains are required for proper assembly of the LTβR-SC (Fig. 3E). Since we already established the catalytic activity of HOIP to be required for activation of LTβR-induced canonical NF-κB signaling, we next determined the targets of linear ubiquitination within the LTβR-SC. To do so, we performed a linear ubiquitin pulldown (M1-AP) under denaturing conditions using a specific linear ubiquitin binder we previously established [37]. This analysis revealed that TRAF2, TRAF3 and NEMO, but neither NIK nor LTβR, were linearly ubiquitinated upon LIGHT stimulation (Fig. S3).

We next determined the functional impact of HOIP on LTβR signaling by performing a Gene Set Enrichment Analysis (GSEA) comparing the results obtained by LC–MS/MS on the composition of the native LTβR-SC obtained from WT vs. HOIP-deficient cells using the quantitative proteomic data obtained from five independent experiments. Whereas the protein signatures obtained in WT cells positively correlated with pathways involved in cytokine-mediated signaling, regulation of the immune response and signal transduction, the protein signatures from HOIP-deficient cells presented a strong negative correlation with these pathways, underscoring the importance of HOIP for the correct function of LTβR-induced signaling (Fig. 3F).

Together, these results identify LUBAC as a bona fide component of the native LTβR-SC, where it places linear ubiquitin chains on different components of the complex, including TRAF2, TRAF3 and NEMO. Moreover, these linear ubiquitin chains are necessary for proper recruitment of NEMO, OPTN, A20 and TBK1 and they are crucial for optimal activation of LTβR-driven canonical NF-κB signaling and for limiting activation of the non-canonical NF-κB pathway. Thereby, LUBAC regulates the activation of different inflammatory and immuno-modulatory signaling pathways induced via LTβR.

### NEMO, A20 and OPTN cooperatively and inversely regulate LTβR-mediated NF-κB activation

**Fig. 4:**
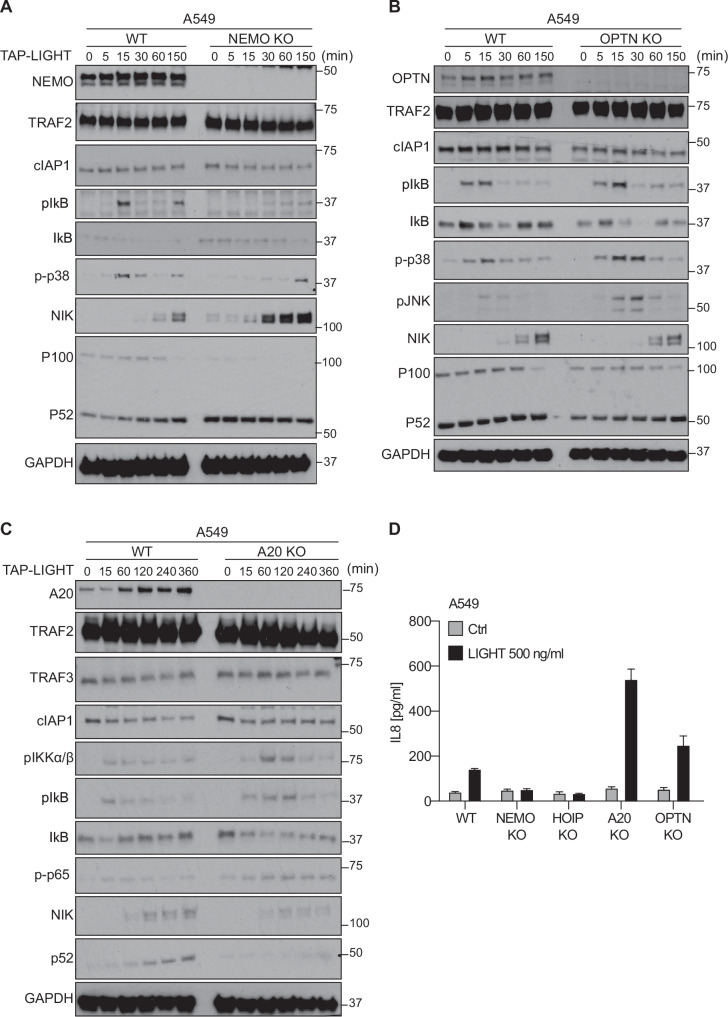
NEMO, A20 and OPTN cooperatively and inversely regulate LTβR-mediated NF-κB activation. **A**–**C** A549 WT or lacking expression of NEMO (**A**), OPTN (**B**) or A20 (**C**) were stimulated with TAP-LIGHT (200 ng/ml) for the indicated times followed by total protein extraction. Cell lysates were subjected to western blot analysis and probed for the indicated proteins. Representative results of at least three independent replicates are shown. **D** A549 WT or lacking expression of NEMO, OPTN or A20 were stimulated overnight with TAP-LIGHT (500 ng/ml). Supernatants were collected and levels of IL8 were quantified by ELISA. Error bars represent the mean ± SEM of three independent experiments. Statistical significance was assessed by two-tailed Student’s t-test. *p < 0.05, **p < 0.01, ***p < 0.001, ****p < 0.0001.

We next sought to analyze the functional role of the three linear-ubiquitin-dependently recruited factors NEMO, A20 and OPTN in LTβR signaling. To that end, we employed CRISPR/Cas9 to generate A549 cells lacking NEMO, OPTN or A20 and analyzed their respective LTβR signaling output by Western blotting. Expectedly, absence of NEMO completely blunted activation of canonical NF-κB, but it also attenuated MAPK activation, albeit not to the same extent as HOIP deficiency, and strongly increased non-canonical NF-κB activation as evidenced by a marked accumulation of NIK and a more pronounced decrease of the ratio p100/p52 (Fig. 4A). By contrast, OPTN-deficient cells presented slightly increased activation of the canonical NF-κB and MAPK pathways as well as a slightly decreased accumulation of NIK and p100/p52 processing upon LIGHT-mediated LTβR stimulation (Fig. 4B). We obtained similar findings with A20-deficient cells, as we observed increased canonical and decreased non-canonical NF-κB pathway activation upon LIGHT stimulation in these cells as compared to WT cells (Fig. 4C). The latter finding is in accordance with the known role of A20 as a negative regulator of canonical NF-κB activation in TNF signaling [37, 61, 62].

Importantly, the pattern of canonical NF-κB activation obtained by Western blotting in each mutant cell line correlated with the LIGHT-induced secretion of IL-8, as cells deficient for OPTN or A20 showed a clear increase in IL-8 secretion, whilst it was completely abrogated in NEMO-deficient cells (Fig. 4D). This was also true for cells expressing a truncated version of A20 lacking the ZnF7 domain (A20ΔZnF7) (Fig. S4A-C) which cannot bind to linear ubiquitin chains [37]. Importantly, LIGHT-induced IL-8 secretion was also blunted in HOIP-deficient cells (Fig. 4D), validating a functional link between HOIP and NEMO regarding activation of canonical NF-κB signaling upon LTβR stimulation. These results demonstrate that LUBAC and linear ubiquitin, together with NEMO, OPTN and A20 are crucial for regulating the balance between the canonical and non-canonical NF-κB pathways in LTβR signaling.

### Deficiency in NEMO, A20 and OPTN differentially affects LTβR-SC composition

**Fig. 5:**
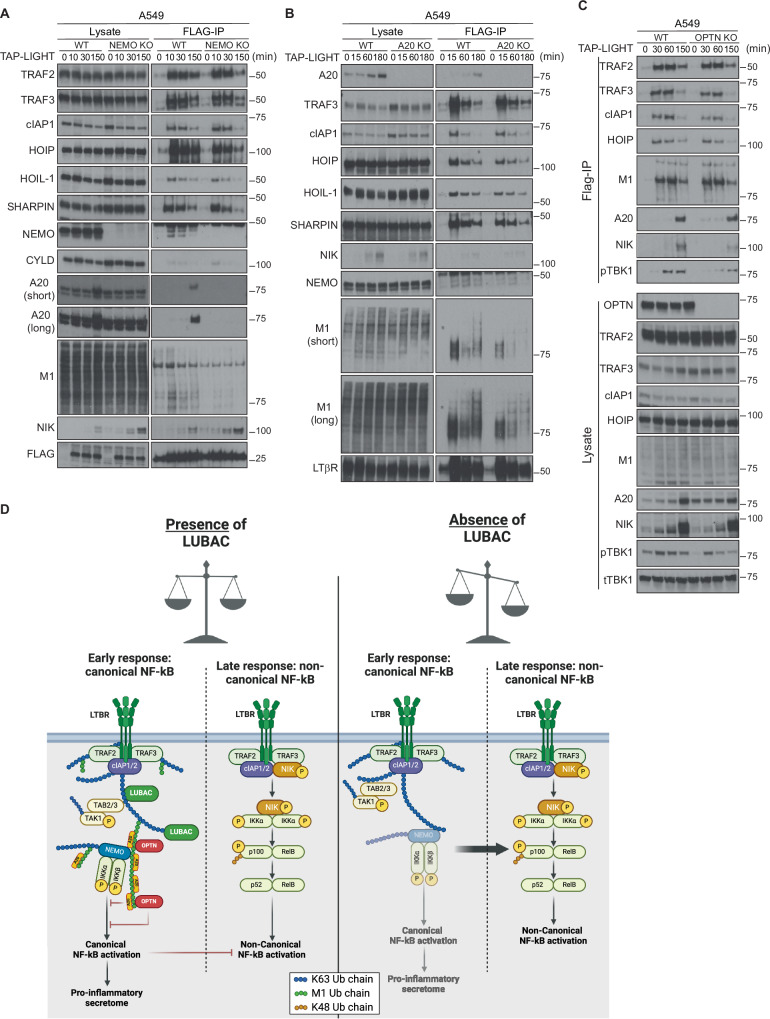
Loss of NEMO, A20 and OPTN differentially affect composition of the LTβR-SC. **A**–**C** A549 WT or lacking expression of NEMO (**A**), A20 (B) or OPTN (**C**) were stimulated with TAP-LIGHT (2000 ng/ml) for the indicated times followed by total protein extraction. Native LTβR-SC was isolated by using M2-beads and analyzed by Western blotting. Representative results of at least three independent replicates are shown. **D** Model of the regulation of LTβR signaling by LUBAC, NEMO and OPTN. In normal conditions, LTβR triggers a balanced activation of canonical and non-canonical NF-κB pathways. This balance is controlled by LUBAC mediated linear ubiquitination of several components within the LTβR-SC, which enables the recruitment of NEMO, OPTN and A20; NEMO positively regulates the canonical NF-κB pathway, whereas OPTN and A20 are negative regulators of this pathway and are involved in the late switch towards activation of the non-canonical NF-κB pathway. Absence of LUBAC causes an impaired recruitment of NEMO, A20 and OPTN, disrupting the signaling balance and causing an uncontrolled activation of the non-canonical NF-κB pathway.

To further dissect the respective roles of NEMO, A20 and OPTN at the LTβR-SC as well as the interplay between them and LUBAC, we analyzed the composition of the LTβR-SC in cells lacking expression of either NEMO, OPTN or A20. A549 cells lacking NEMO presented a stark decrease in linear ubiquitination in the LTβR-SC, whereas all three LUBAC components were still recruited to the complex, albeit slightly less efficiently than in cells expressing NEMO (Fig. 5A). This result is in line with the identification of NEMO as a target of linear ubiquitination within the LTβR-SC (Fig. S3), but also potentially with NEMO as a binder and, consequently, protector of linear ubiquitin chains. Intriguingly, in the absence of NEMO, the presence of NIK was not only increased in total lysates but also at the LTβR-SC (Fig. 5A), indicating that the dysregulation of the balance between canonical and non-canonical NF-κB pathways occurs at the level of the LTβR-SC.

Loss of A20 or its ZnF7 domain caused an overall reduction of linear ubiquitination within the LTβR-SC, along with a slight decrease in the recruitment of the three LUBAC components (Figs. 5B and S4A). These results are in line with previous reports on the role of A20 in the TNFR1-SC and the NOD2-SC in which A20 was described to protect linear ubiquitin chains from deubiquitinase (DUB)-mediated degradation and to thereby stabilize these signaling complexes [37]. Remarkably, in NEMO KO cells, A20 recruitment is completely abrogated (Fig. 5A), whilst in A20 KO cells, recruitment of NEMO to the LTβR-SC, is only slightly reduced (Fig. 5B). Taking into consideration that in A20-deficient cells there is increased canonical NF-κB activation (Fig. 4C), these results imply an intricate co-dependency between NEMO and A20 downstream of LUBAC, with NEMO enabling the recruitment of A20 to restrict NF-κB signaling in a negative feedback loop regulatory mechanism.

In OPTN-deficient cells, recruitment of NIK to the LTβR-SC appears to be reduced, whereas neither linear ubiquitination nor recruitment of LUBAC components are affected (Fig. 5C). This correlates with the imbalance on NF-κB activation upon LIGHT stimulation in the absence of OPTN observed by Western blot (Fig. 4B), shifted towards canonical NF-κB, and implies a functional antagonism between NEMO and OPTN in LTβR-mediated NF-κB regulation. Since OPTN has previously been shown to serve as an adaptor for TBK1 activation in certain signaling pathways [63], albeit not in others, including in TNFR1 signaling [31], and because we observed that activation of this kinase required HOIP (Figs. 1 and 2), we next explored whether activation and recruitment of TBK1 upon LTβR activation, which we found to be also dependent on LUBAC and its activity (Fig. 3C and SF5A, B), could be mediated by OPTN, downstream of HOIP. Indeed, recruitment of TBK1 to the LTβR-SC was dependent on OPTN (Fig. 5C and SF5C). Interestingly, and contrary to our previous findings in TNFR1 signalling [31], absence of NEMO reduced neither TBK1 activation upon LTβR stimulation (SF5E) nor its recruitment to the LTβR-SC (SF5D). Intriguingly, cells lacking both TBK1 and IKKε did not show defects in canonical (S5F, G) or non-canonical (S5F) NF-kB activation upon LIGHT stimulation. These results imply that OPTN does not only participate in modulating the balance between LTβR-induced canonical and non-canonical NF-κB activation, but that it is also required for optimal TBK1 activation upon LTβR stimulation.

Together, our results demonstrate that linear ubiquitination within the LTβR-SC enables the recruitment of both NEMO and OPTN, where they exert opposing effects in the regulation of canonical versus non-canonical NF-κB activation. A20, whose recruitment to the LTβR-SC requires NEMO, in turn restricts activation of the canonical NF-κB pathway and enables the activation of non-canonical NF-κB signaling (Fig. 5D). These findings reveal a prominent role of LUBAC as a crucial factor ensuring a correct signaling outcome from the LTβR-SC, and provide a possible explanation for how, in the absence of HOIP, LTβR-mediated canonical NF-κB signaling is impaired whilst non-canonical NF-κB signaling is activated.

### HOIP is a novel prognostic marker in liver cancer patients with high LTβR expression

**Fig. 6:**
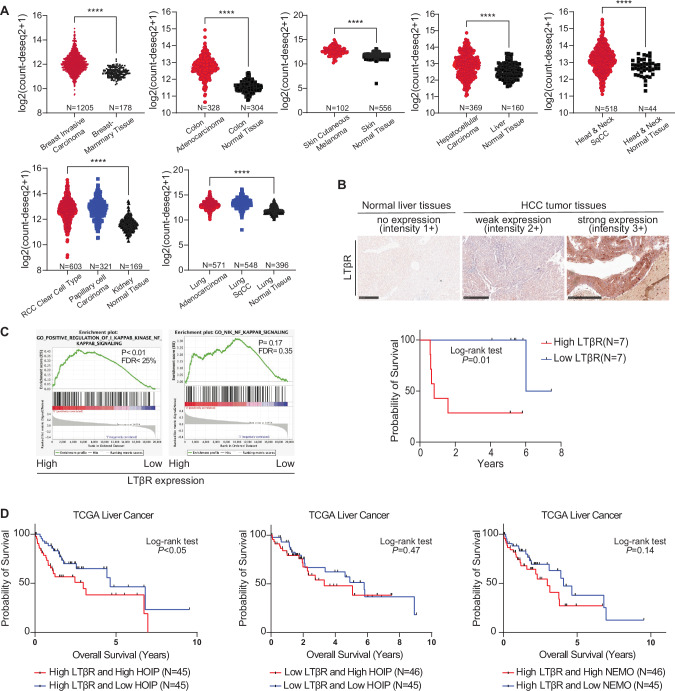
HOIP is a novel prognostic marker in liver cancer patients with high LTβR expression. **A** RNA-seq expression data obtained from TCGA and GTEx databases were analyzed. All normalized expression data were downloaded from UCSC Xena platform (unit: log2(expected_count-deseq2 + 1)). Generally, the transcripts of LTβR in tumor tissues were retrieved from TCGA database with different cancer types. The normal tissues from different organs in this analysis were obtained from GTEx and TCGA matched normal samples. Statistical significance was calculated using a two tailed student’s t test, and the asterisks indicate the statistical significance (* means: *p* < 0.05, **** means: *p* < 0.001). **B** Primary samples obtained from 14 liver cancer patients were stratified according to their LTβR expression (quantified by IHC staining intensity), and their respective survival rates were plotted in a Kaplan-Meier survival graph. Representative IHC images of normal tissue (negative control), low LTβR expression and high LTβR expression are shown. Statistical significance was calculated using a log-rank test, and p value is shown in the graph. **C** GSEA of RNA-seq expression data from TCGA liver cancer database (LIHC), comparing patients with high expression of LTβR (n = 183) versus low expression of LTβR (n = 182). Correlation between expression levels of LTβR and gene signatures of either canonical NF-κB pathway (GO:0043123) or non-canonical NF-κB pathway (GO:0038061) are analyzed. The median value of normalized mRNA transcripts was set up as a cut-off value for separating high expression and low expression LTβR group. **D** RNA-seq expression data from TCGA LIHC was used to stratify patients in two groups according to their LTβR expression. The median value of normalized LTβR mRNA transcripts was set up as a cut-off value for separating high and low expression groups. Subsequently, in the high LTβR expression group, patients were stratified again into high- or low-HOIP expression groups based on the quartile value, and survival results were represented in Kaplan-Meier survival graphs. Statistical significance was calculated using a log-rank test, and *p* values are shown in each graph.

LTβR-driven signaling mediators are considered as a prognostic factor in some cancer types, such as liver, head/neck and kidney cancer [19, 21, 64]. Because of the crucial role we uncovered for LUBAC in LTβR signaling, we next set out to determine whether expression of essential LUBAC components or the decisive downstream factor for canonical NF-κB activation we found to be recruited to the LTβR-SC in a linear-ubiquitin dependent manner, i.e. NEMO, could also be involved in the pathogenesis of LTβR-expressing tumors. To that end, we first performed a bioinformatic analysis of RNA-sequencing data obtained from the publicly available database, TCGA, through the UCSC Xena platform (https://xena.ucsc.edu/) [65]. We only included data from patients diagnosed with primary tumors, excluding recurrent or metastatic cases. As shown in Fig. 6A, we found expression of LTβR to be consistently increased in a variety of tumor entities as compared to corresponding adjacent normal tissues. We next analyzed the impact of LTβR expression on patient survival in different cancer types. High LTβR expression negatively correlates with overall survival in patients with liver cancer, lung adenocarcinoma, RCC (clear cell subtype), and HNSCC (Fig. S6A). This correlation was also observed for rectal cancer patients, albeit with an increase in the width of confidence intervals, likely as a consequence of a wider spread in overall survival in this disease (Fig. S6A). We next analyzed expression levels of LTβR in primary samples obtained from liver cancer patients by immunohistochemistry. A specificity control for LTβR staining was carried out in A549 cells lacking LTβR (Fig. S6B). A total of 14 liver cancer patients were stratified into high and low LTβR expression based on staining intensity for LTβR (Fig. 6B). In agreement with the RNA-sequencing data, high expression of LTβR correlated with poor prognosis of these patients (Fig. 6B).

The GSEA analysis of transcriptomic data demonstrated that only canonical NF-κB target genes were enriched in patients with high expression levels of LTβR, whereas there was no correlation between the gene signatures of non-canonical NF-κB target genes with expression of LTβR (Fig. 6C). Furthermore, in a correlation matrix LTβR only positively correlated with IL-8 and CCL20 (besides ICAM1) (Fig. S6C), which is entirely in line with the results we obtained when examining chemo-/cytokine induction by LTβR stimulation (Fig. 1A, B). When examining the impact of IL-8 and CCL20 expression on patient survival in the TCGA HCC data set, we detected that high expression of IL-8 or CCL20 negatively correlated with overall survival in HCC patients (Fig. S6D). Other members of the TNFRSF, as well as receptors of the Toll like receptor (TLR) family, can modulate secretion of pro-inflammatory chemo-/cytokines in cancer cells [59, 66, 67]. We therefore next explored to what extent the expression of LTβR as compared to that of other immune receptors affected patient outcome. We analyzed the correlation between activation of different pro-inflammatory pathways and patient survival in lung and liver cancer patients using the Gene Expression Profiling Interactive Analysis platform (GEPIA2, http://gepia2.cancer-pku.cn/#index) [68]. Intriguingly, amongst the relevant pro-inflammatory pathways analyzed, LTβR is the signaling pathway that most prominently correlates with the hazard ratio (HR) (Fig. S6E).

Because of our discovery of a crucial role of LUBAC and the linear ubiquitin chain-forming activity of its component HOIP for LTβR-induced canonical NF-κB signaling, we next examined whether the expression of LUBAC, and in particular of HOIP, could affect the overall survival of liver cancer patients with high or low LTβR expression. This analysis revealed a striking and highly significant negative correlation between overall survival and HOIP expression in patients with high LTβR expression (Fig. 6D). Interestingly, a similar, albeit statistically not significant, trend is also observed for patients expressing high NEMO (Fig. 6D). Additionally, we investigated whether the prognostic value of LTβR and HOIP expression could be attributed to infiltrating immune cells, which might affect our data interpretation. Using CIBERSORT, we found no statistical significance in the fractions of most infiltrating immune cell subtypes when comparing patients with high LTβR and high HOIP expression to those with high LTβR but low HOIP expression (Fig. S7A). However, we observed significantly lower levels of activated NK cells in patients with high LTβR and high HOIP expression compared to those with high LTβR and low HOIP expression (Fig. S7B). This analysis suggests that LTβR signaling may not primarily affect immune cells in the context of liver cancer but rather the tumor cells themselves. Additionally, we investigated the impact of another LUBAC component, HOIL-1, which positively regulates NF-κB signaling, on the overall survival of liver cancer patients with high LTβR expression. This analysis showed a similar trend to the effect observed with HOIP expression in liver cancer patients with high LTβR expression (Fig. S7C). This result provides further support for the notion that LUBAC and linear ubiquitination, rather than HOIP in isolation, are involved in tumorigenesis. Together, these results suggest that in HCC patients with high LTβR expression, the expression level of HOIP may serve as a previously unrecognized prognostic marker and that inhibition of LUBAC may be of therapeutic value, especially in such patients.

## Discussion

Signaling mediated by LTβR is crucial for the homeostasis of secondary lymphoid tissue, being mostly implicated in lymphoid neogenesis and the development of tertiary lymphoid structures [69, 70]. More recently, a crucial role for LTβR signaling in initiating inflammation-induced carcinogenesis was discovered [22]. However, despite these important functions of LTβR signaling, prior to this study only a few downstream regulators of LTβR signaling were known and information on the composition of the LTβR-SC was particularly scarce.

Seeking to shed light on the molecular regulation of LTβR signaling, we performed an unbiased proteomic analysis of the composition of the native LTβR-SC. Apart from identifying several other factors previously not known to form part of this complex, this analysis identified all three LUBAC components, HOIP, HOIL-1 and SHARPIN, as previously unrecognized bona fide components of the LTβR-SC. Given the prominent role of LUBAC and its product, linear ubiquitin chains, in other immune receptor signaling complexes, we went on to study their role in LTβR signaling in detail.

In line with previous studies, we observed a sequential activation of both canonical and non-canonical NF-κB pathways by Western blotting upon activation of LTβR in the three cell lines we tested. Functionally, this activation translated into secretion of a distinct chemo-/cytokine profile, mainly composed of pro-inflammatory factors such as IL-8 and CCL20. Despite the fact that non-canonical NF-κB was activated in these cell lines upon LTβR stimulation, we could not detect any of the chemo-/cytokines thought to be driven by LTβR-mediated non-canonical NF-κB signaling, including CXCL13, CCL19 or CCL21. This result suggests that non-canonical NF-κB signaling is required but not sufficient for the expression of these chemo-/cytokines upon LTβR stimulation. Independently thereof, our data shows that, in cancer cells, activation of LTβR induces secretion of pro-inflammatory cytokines known to be tumorigenic, and that this requires activation of the canonical NF-κB pathway which is, in turn, activated in a LUBAC- and linear-ubiquitin-dependent manner.

Although a counterbalance between canonical and non-canonical NF-κB pathways has been known for years [71], so far the mechanistic details that govern this crosstalk remained elusive. In the present study, we shed light on the molecular events that control this balance by identifying a completely new layer of regulation exerted by the coordinated action of OPTN, NEMO and A20 downstream of LUBAC. Our results show that, upon activation by LIGHT, LUBAC is rapidly recruited to the LTβR-SC where it places linear ubiquitin chains on several components, including TRAF2, TRAF3 and NEMO, with the latter being recruited in a linear-ubiquitin-dependent manner. These linear ubiquitin chains enable recruitment of OPTN and A20 and further recruitment of NEMO which, together, regulate the LTβR signaling output by activating canonical NF-κB and MAPKs and by keeping canonical and non-canonical NF-κB signaling in balance. NEMO is a key initiator of the canonical NF-κB pathway, serving as an adaptor for IKKα and IKKβ and mediating their activation. In general terms, for NEMO to be recruited to signaling complexes, it requires binding to K63- or, preferably, M1-linked ubiquitin chains via its UBAN domain [72]. Accordingly, we observed that activation of the canonical NF-κB pathway was greatly diminished in HOIP-deficient cells, correlating with a severely blunted recruitment of NEMO to the LTβR-SC. In turn, HOIP-deficient cells presented an earlier and increased activation of the non-canonical NF-κB pathway. This result is in line with previous reports of an inhibitory role of NEMO on activation of non-canonical NF-κB signaling [71]. Thus, our results show that linear ubiquitination within the LTβR-SC exerted by LUBAC is required for proper activation of canonical NF-κB.

A20 has been widely described to negatively regulate canonical NF-κB activation in several signaling pathways [73]. In the TNFR1-SC, A20 binds to linear ubiquitin chains via its ZnF7 domain and protects them from degradation [32]. We and others previously proposed that the negative impact of A20 on canonical NF-κB activation might be due to a direct competition with NEMO for binding to the same linear ubiquitin substrates [37, 74]. It was also suggested, however, that NEMO is necessary for A20 recruitment to certain signaling complexes by serving as an adaptor [74]. The results we present here in the context of the LTβR-SC are in agreement with the latter, as recruitment of A20 to the LTβR-SC was completely abrogated in NEMO-deficient cells. Interestingly, we also found that the recruitment of A20 to the LTβR-SC via NEMO was mediated by the ZnF7 domain of A20, in agreement with what we previously described for the TNFR1-SC [37, 73]. The most plausible conclusion we can draw from our findings is that recruitment of A20 to the LTβR-SC is mediated by an interaction between the ZnF7 domain of A20 and a NEMO-linked linear ubiquitin chain. Together, our results suggest an intricate co-dependency between NEMO and A20 downstream of LUBAC in LTβR signaling, according to which NEMO enables the recruitment of A20 to restrict NF-κB signaling in a negative feedback loop regulatory mechanism. In support of this conclusion, recruitment of A20 to the LTβR-SC in normal wild-type cells is consistently observed after the first wave of canonical NF-κB activation. Thus, in the absence of A20 a suboptimal level of linear ubiquitination and NEMO recruitment appears to be sufficient to promote canonical NF-κB. Whether the inhibitory role of A20 on canonical NF-κB activation depends on the catalytic OTU domain of A20 or on other functions of A20 warrants further investigation. That said, the proposed mechanism provides a plausible explanation for the seemingly contradictory phenotype of A20-deficient cells, with a reduced recruitment of NEMO to the LTβR-SC but increased activation of canonical NF-κB.

Although LUBAC is a component of both the TNFR1-SC and LTβR-SC, there are fundamental differences between both ligand-receptor systems. Whilst both receptors share a certain degree of similarity in the composition of the respective signaling complexes, the interplay between LUBAC, OPTN, A20 and NEMO in the LTβR-SC that we determined here is decisively distinct from that in the TNFR1-SC and other immune receptor-associated SCs. It is likely that one of the determinants of such differences is the presence of OPTN, which is recruited to the LTβR-SC in a LUBAC-dependent manner, but does not form part of the TNFR1-SC [31, 37, 75]. Interestingly, despite the structural similarity between NEMO and OPTN, with both factors containing a UBAN domain, which enables the binding to linear ubiquitin chains [40], these two proteins play opposite roles in the regulation of canonical NF-κB [76]. Moreover, OPTN has been described to compete with NEMO for binding to ubiquitin chains [77]. Our results are in line with these observations, as canonical NF-κB activation was increased upon LIGHT stimulation in cells lacking OPTN. Additionally, our data also revealed that OPTN is required for the adequate recruitment to, and activation at, the LTβR-SC of TBK1 upon LIGHT stimulation in a LUBAC-dependent manner.

Intriguingly, and contrary to the current dogma in which NIK exerts its activity as a cytosolic protein, we consistently observed specific recruitment of NIK to the LTβR-SC upon LIGHT stimulation in different cell lines. In WT cells, the recruitment kinetics were similar to those of A20, with NIK being more prominently recruited after 60 min of stimulation. This suggests that the NIK that is recruited to the LTβR-SC is a product of de novo protein translation induced in response to the initial activation of LTβR. Although further studies are necessary to dissect the mechanism of this recruitment, we hypothesize that this may be related to OPTN. Supporting this notion, our results show that NIK recruitment to the LTβR-SC is negatively affected in OPTN-deficient cells. Thus, similarly to TBK1, NIK recruitment to the LTβR-SC could also be mediated, at least in part, by OPTN. Of note, the role of OPTN as mediator of the recruitment of TBK1 and NIK to the LTβR-SC in a linear-ubiquitin-dependent manner has not been described before. TBK1 has previously been described to act as a negative regulator of non-canonical NF-kB by phosphorylating NIK, triggering its degradation [78]. Although we could not identify any functional role of TBK1 in LTβR signaling, it is tempting to speculate that TBK1 is involved in regulating the balance between canonical and non-canonical NF-kB pathways by controlling NIK levels at the LTβR-SC. Further studies using different experimental models will, however, be required to address this hypothesis.

Although our results establish the presence of LUBAC within the LTβR signaling complex and its impact on the downstream signaling pathways emanating from it, the mechanism of LUBAC recruitment into the LTβR-SC remain unclear. A possible mechanism can be inferred from the known biochemistry of LUBAC in other signaling complexes, such as the TNFR1-SC or the TRAILR-SC [79] and from the previous observation that inhibition of cIAP1/2 impairs NF-kB activation upon LIGHT stimulation. Thus, the recruitment of LUBAC to the LTβR-SC is likely dependent on the presence of K63-linked ubiquitin chains placed within the complex by cIAP1 and/or cIAP2. The presence of proteins such as TRAF2, cIAP1 and cIAP2 in the LTβR-SC supports this hypothesis. Nevertheless, future experiments will be required to test this hypothesis, not only in LTβR-SC but also in other TNFR-SC.

Interestingly, Jain et al. recently reported on the relevance of LUBAC for thymic epithelial homeostasis. In this study, conditional deletion of HOIP or HOIL-1 in thymic epithelial cells (TECs) induced severe thymic atrophy, concurring with loss of medullary and cortical TECs in the thymi of these mice [80]. Curiously, this phenotype was determined to be cell death-independent, despite the prominent role of LUBAC at preventing programmed cell death in several signaling pathways. In light of the results presented here, and considering that LTβR signaling is essential for survival, maturation and expansion of mTECs [81], a plausible explanation for the observed phenotype could be that absence of LUBAC impairs LTβR-derived canonical NF-κB signaling and that this interferes with normal thymic development. It would be interesting to investigate whether reactivation of canonical NF-κB signaling in *Hoil-1*^*TEC-KO*^ mice restores normal thymic development.

The role of LUBAC and linear ubiquitin in liver cancer has previously been reported by us and others [60, 82, 83] and the impact of LTβR signaling in hepatocarcinogenesis has also been studied [19]. Yet, a connection between the two has so far not been established. Because of the biochemical and functional results we obtained in our study we were prompted to determine whether there could be a connection between LUBAC and LTβR in cancer. Of note, LTβR signaling has been shown to be involved in the pathogenesis of many human diseases, not only cancer but also several autoimmune diseases such as, e.g., diabetes [8]. Moreover, high LTβR expression is considered a poor prognostic marker in liver cancer patients [19], an observation which is corroborated by the bioinformatic analysis we present here. Furthermore, increased levels of IL-8 and CCL20 have been reported in the tumor tissue of HCC patients, correlating with poor prognosis and decreased overall patient survival [84–86]. IL-8 and CCL20 functionally contribute to progression, invasion and metastasis of liver cancer [87–89]. Our results provide a direct link between the LUBAC and LTβR-induced activation of canonical NF-κB signaling and secretion of IL-8 and CCL20 by cancer cells. Intriguingly, in HCC patients we also observed a positive correlation of high LTβR expression with high levels of IL-8 and CCL20 and decreased survival probability. Hence, our combined unbiased proteomic, functional and bioinformatic analysis suggests that LUBAC- and linear ubiquitin-dependent LTβR-derived canonical NF-κB signaling is responsible for triggering the secretion of the pro-inflammatory chemokines IL-8 and CCL20 which, in turn, could potentially drive tumor progression and negatively impacts HCC patient survival. It is important to note that amongst the relevant pro-inflammatory pathways analyzed, including TNFRSF and TLR family members, LTβR is the signaling pathway that most prominently correlates with the hazard ratio (HR).

Analysis of HCC patient-derived expression databases identified HOIP as a novel prognostic marker in patients with high LTβR expression, but not in those with low LTβR expression. A similar trend was also observed in patients with high LTβR expression stratified by high *versus* low NEMO expression. Accordingly, LUBAC appears to be particularly important for sustaining pro-tumorigenic LTβR signaling in patients with high LTβR expression, whereas it seems less critical in patients with low LTβR expression. Although these concepts are exciting, validating the relationship between LTβR, HOIP expression and LUBAC activity in preclinical models and in human cancers requires further investigation. Another caveat of this analysis is that, even though we deem this likely, we cannot confirm that high expression of HOIP and HOIL-1 would translate into high LUBAC activity in cancer cells. Yet, it is important to highlight that overexpression of HOIP was reported to be sufficient to promote DLBCL-like B-cell lymphomagenesis [90]. Therefore, high expression of HOIP in tumor cells is likely translated into high LUBAC activity, even more so if this correlates with enhanced LTβR expression.

Collectively, the clinical observations align with the mechanistic results we obtained in this study, i.e. the identification of LUBAC as crucial for LTβR-mediated canonical NF-κB activation, as it implies that LTβR stimulation exerts pro-tumorigenic activity by inducing a pro-inflammatory secretome in a manner that requires the HOIP-activity-dependent activation of canonical NF-κB. These results suggest that, indeed, LUBAC-dependent increased cytokine production by LTβR, rather than by DRs, is responsible for creating a tumor-promoting environment in these cancers. Hence, therapeutic targeting of LTβR signaling, either by small molecule inhibitors of factors or activities identified here as crucial for LTβR signaling, e.g. LUBAC or the linear-ubiquitin-chain-forming activity of HOIP, or by using biotherapeutics that neutralize LTβR ligands or their interaction with LTβR, e.g. LTβR-Fc, could prove clinically useful to treat certain types of cancers, including HCC.

## Material and methods

### Preparation of recombinant proteins

#### Molecular cloning of the DNA sequence encoding extracellular LIGHT

THP-1 cells were incubated with 100 ng/ml para-Methoxyamphetamine (PMA) for 24 h followed by 10 ng TNF stimulation for 4 h. After that, cells were harvested, and total RNA was isolated by using RNeasy mini spin kit (Qiagen RNeasy Kits). 1ug of total RNA was converted to cDNA by reverse transcription using a High-Capacity cDNA Reverse Transcription Kit (ThermoFisher Scientific). Next, the extracellular TNF homology domain of LIGHT (aa 73–240) was amplified by using the following primers: atAGATCTcTAGGAGAGATGGTCACCCGCCT and atGTCGACTCACACCATGAAAGCCCCGAA. The obtained amplicon was purified and subcloned into a customized pQE30 vector between the sites BglII and SalI, keeping the reading frame with a six Histidine‐tag followed by 3xFLAG, a PreScission™ protease cleavage site and a 2xStrep‐tag II (construct schematics in Fig. 1C). The resulting construct (moTAP-LIGHT) was validated by Sanger sequencing.

#### Production of recombinant TAP-LIGHT and TAP-TNF

Competent *Escherichia coli* BL21-Codon Plus (DE3-RP) cells were transformed with a pQE30 plasmid containing a TAP-TNF sequence (previously generated in our laboratory and described in Draber et al. [37] Kupka et al. [75]) or the newly generated TAP-LIGHT construct. Recombinant TAP-TNF or TAP-LIGHT were subsequently produced and purified as previously described [37, 75]. Briefly, the recombinant proteins were expressed in BL21 (DE3) cells by addition of 1 mM isopropyl b-D-1-thiogalactopyranoside (IPTG) overnight and purified by affinity chromatography on His GraviTrap TALON columns (GE Healthcare), eluted with 500 mM imidazole and dialyzed against storage buffer (50 mM Tris [pH 7.4], 100 mM NaCl, 0.02% Tween, 2 mM DTT, and 0.5 M arginine). Protein concentration was determined with a Nanodrop 2000 (Thermo Scientific) and purified recombinant proteins were stored at −80 °C.

### Generation of stably transfected knockout cells

The cell lines JHH4, HLE, PLC and HSC3 cells were kindly gifted by Mr. Znati Sami and Dr. Kenrick Ng, respectively. Hep3B, A549, THP-1, Raji, and Hela cancer cell lines were purchased from the American Type Culture Collection. In some cases, expression of HOIP (RNF31), A20, NEMO or LTβR was abrogated by CRISPR/Cas9 using the following sgRNA sequences: RNF31: 5′-CGAGATGTGCTGCGATTATA, A20: 5′-GGCGCTGTTCAGCACGCTCA, optineurin: 5′-GATTTGAGGAGCTTTCGGCC, NEMO: 5′-CGGCAGCAGATCAGGACGTAC, TBK-1: 5′-TTTGAACATCCACTGGGCGA, LTβR: 5′-GTCTGGTTCTCCGACGCATA. Briefly, each individual sgRNA was subcloned into the plasmid- pSpCas9(BB)-2A-GFP (PX458, Addgene plasmid #48138). Subsequently, cells were transfected with the plasmids, and GFP-positive cells were sorted into 96-well plates. Single-cell clones were analyzed 4 weeks later by Western blot and Sanger Sequencing to assess KO status. The surviving clones were passed for several generations followed by determining the KO using Western blotting. The KO efficiency was evaluated by ICE Crispr analysis tool. (Synthego, URL: https://www.synthego.com/products/bioinformatics/crispr-analysis). Reconstitution of HOIP KO cells with either HOIP WT or HOIPC885S, was achieved by retroviral transduction as previously described [31, 37, 59]. Briefly, the packaging cells Phoenix-AMPHO (Cell Biolabs,Inc) were transfected with the vector pBabe-puro (Addgene) containing either the WT or the C885S mutant sequences of HOIP by using Lipofectamine 2000 (ThermoFisher Scientific). Two days later, viral supernatants were collected, cleared and filtered, and A549 HOIP KO cells were infected and then selected with puromycin for 4 weeks. All cell lines were regularly tested for mycoplasma using MycoAlert™ Mycoplasma Detection Kit (LONZA).

### Inhibitors and antibodies

The following inhibitors were used in this study (final concentration is indicated in parenthesis): TPCA-1 (5 µM; Tocris Bioscience); B022 (25 µM; MedChem express); 7-oxozeanol (10 µM; Tocris Bioscience); Nec-1s (10 µM; Biovision); zVAD-FMK (20 µM; Abcam); SMAC mimetic (Selleckchem). TNFR1-FC was purchased from Pfizer, and was used at 50 µg/ml. All antibodies utilized in flow cytometry, Western blot, immunoprecipitation, and immunohistochemistry study are listed in Supplementary Table 3.

### Cell proliferation assay

Cells were seeded in 6 well plates at a density of 2 × 10^3^ /well. 24 h later, the cells were treated as indicated or left untreated, in which case medium without FCS was added. 18 h prior to the end time point (96 h), the BrdU reagent (Merck Millipore) was added to the wells at 1:1000 dilution from the stock. At the endpoint, the cells were fixed using the supplied solution and stored at 4 °C. The following day, cells were washed with PBS and then incubated with the BrdU detection antibody for 1 h. The peroxidase conjugated secondary antibody was added and incubated for 30 min. After a final water wash step, the TBM peroxidase solution was added. After an additional 30 min, 2 N H_2_SO_4_ was added to stop the reaction and the absorbance was read at 450 nm. The raw OD values were used for quantification of BrdU incorporation.

### Viability and cell death assays

#### CellTiter-Glo Luminescent cell viability assay

Cells were seeded in 96 well plates in triplicates at a density of 15,000 cells/well. The following day, cells were treated with the indicated compounds, and left overnight. The following day, cell viability was measured by CellTiter-Glo (Promega) as previously described [31, 37, 59]. Briefly, the reconstituted CellTiter-Glo reagent was diluted 1:4 in PBS, and 20 µl of this solution were added to the cells and incubated for 12 mins. Next, 100 µl of the mixture were collected and transferred to an opaque 96 well plate, and luminescence emission was measured using a Mithras LB 940 plate reader (Berthold Germany). Viability was calculated as a percentage of luminescence relative to untreated control.

#### Real-time cell death monitoring system

Cells were seeded in 96 well plates in triplicates at a density of 20,000 cells/well. The following day, cells were treated with recombinant human TRAIL (1000 ng/ml), recombinant human TNF or human LIGHT in the presence of 5 µM Sytox Green (Thermo Fisher Scientific), and the plates were immediately placed inside an IncuCyte device (Essen Bioscience). Cells were imaged in real-time for the indicated times in 2 h intervals, and dead cells were quantified as Sytox-Green positive. Percentage of cell death was calculated by a proprietary IncuCyte software application.

### ELISA and Cytokine/chemokine array

All indicated cells were pre-treated with or without TPCA-1 (5 μM), 7-Oxozeaenol (10 μM), or B022 (25 μM) and further stimulated with human TAP-LIGHT (500 ng/ml) for 24 h. The following day, supernatants were collected and centrifuged at 400 × *g* for 5 min to remove cell debris. These cleared supernatants were used to assess cytokine secretion by ELISA (R&D Systems) or by a cytokine/chemokine array (R&D Systems, ARY022B), according to manufacturer’s instructions.

### Immunoprecipitation of native complexes

To analyze the native LTβR-signaling complex, cells were seeded in 10 cm plates. The following day, cells were washed with PBS, and subsequently TAP-LIGHT was added to the cells in medium for the indicated times. Cells were left in the incubator during the course of stimulation. Subsequently, stimulation medium was aspirated, and cells were washed with cold PBS. Cells were lyzed in IP-lysis buffer (30 mM Tris-HCl, pH 7.4, 120 mM NaCl, 2 mM EDTA, 2 mM KCl, 10% Glycerol, 1% Triton X-100, 50 mM NaF, 5 mM Na_3_VO_4_, 1x COMPLETE protease-inhibitor cocktail (Roche)) at 4 °C for 1 h, and cellular debris was cleared by centrifugation at 13,000 rpm for 20 min. 1/10 of the amount of FLAG-LIGHT used for the stimulation was added to lysates from non-stimulated cells as a negative control. 10 mg M2 beads (Sigma-Aldrich) were then added to the lysate and incubated overnight at 4 °C. The next day, samples were washed three times with IP buffer and then reduced in sample buffer.

### M1 Affinity Precipitation (M1-AP)

Pulldown of linear ubiquitin chains was performed by using a recombinant M1-affinity construct as previously described [37] (Draber et al., 2015). Briefly, cells were first detached by scratching in cold PBS containing 1% DTT. Next, AP‐lysis buffer containing 30 mM Tris–HCl, pH 7.4, 120 mM NaCl, 2 mM EDTA, 2 mM KCl, 1× Chloroacetamide, 1% SDS, 1× COMPLETE protease‐inhibitor and 1× PhosSTOP (Roche) was added to the collected cell suspensions. After that, these samples were diluted in 0.1% SDS and then incubated for 10 min on ice, sonicated and cleared by centrifugation at 17,000 × *g* for 30 min. Next, HALO beads (Promega) were incubated with the M1-affinity construct, and these pre-coupled beads were added to the cleared cell lysates and left overnight at 4 °C in rotation. The following day, beads were washed three times using AP‐lysis buffer lacking SDS, chloroacetamide and inhibitors of proteases and phosphatases and then reduced in sample buffer.

### Electrophoresis and Western blotting

Proteins were separated by using 4–15% Mini-PROTEAN® TGX™ Precast Protein Gels and TGX buffer (BioRad). Proteins were then transferred onto a 0.2 mM nitrocellulose membrane (Bio-Rad, Trans-Blot Turbo Mini Nitrocellulose Transfer Packs). On occasion, membranes were treated with stripping buffer containing 50 mM glycine, pH 2.3.

### Tandem affinity purification and mass spec sample preparation

Hep3B cells expressing HOIP WT or HOIP KO (9 × 10^8^ cells each) were first stimulated with TAP-LIGHT at the indicated time points. Cells were subsequently solubilized in IP-lysis buffer (30 mM Tris–HCl, pH 7.4, 120 mM NaCl, 2 mM EDTA, 2 mM KCl, 10% glycerol, 1% Triton X-100, 1× COMPLETE protease-inhibitor cocktail and 1× PhosSTOP (Roche)), cleared by centrifugation (13,000 rpm, 30 min, 4 °C), and incubated overnight with 100 μl of anti-Flag M2-Agarose beads per sample (Sigma). To assess basal nonspecific binding in the IP-MS experiments, beads were added to non-stimulated lysates as negative controls (0 time point). The following day, all samples were washed three times with IP-lysis buffer and proteins were eluted overnight in IP lysis buffer containing 200 μg/mL 3x Flag peptide (Sigma) and 25 U/ml PreScission Protease (GE Healthcare). Samples were subsequently subjected to a second affinity precipitation using Strep-Tactin magnetic resin (IBA) overnight at 4 °C and eluted through strep-tactin BXT elution buffer. Ice-cold acetone was added to the eluates at a ratio of 6:1 (v/v). The samples were vortexed for 30 s before centrifugation (13,000 rpm, 5 min, 4 °C). Proteins were allowed to precipitate overnight at −20 °C. 80% of the supernatant was removed by pipetting and the remainder was allowed to air dry (~20 min, 30 °C). Proteins were resuspended in denaturation buffer (50 mM TEAB, 8 M urea), reduced with 5 mM TCEP at 37 °C for 20 min, alkylated with 10 mM chloroacetamide at 22 °C in the dark for 20 min. Samples were diluted with 50 mM TEAB to reduce the urea concentration to 1.5 M. Subsequently, all samples were digested with trypsin (Promega V5113) (final concentration = 10 ng/uL) for 4 h at 37 °C. Digests were desalted with C18 microspin columns (SEM SS18V; The Nest Group), eluted with 50% acetonitrile 0.1% TFA, evaporated to dryness at 30 °C, and resolubilized in 20 µL 0.1% formic acid in water for LC-MS analysis.

### Mass spectrometry

We performed nLC-MS/MS on an Q Exactive Plus interfaced to a NANOSPRAY FLEX ion source and coupled to an Easy-nLC 1200 (Thermo Scientific). Twenty five percent of each sample were analyzed as 5 µL injections. Peptides were separated on a 27 cm fused silica emitter, 75 µm diameter, packed in-house with Reprosil-Pur 200 C18-AQ, 2.4 µm resin (Dr. Maisch) using a linear gradient from 5% to 30% acetonitrile/ 0.1% formic acid over 30 (Fig. 1D) or 60 min (Fig. 3A), at a flow rate of 250 nL/min. Peptides were ionized by electrospray ionization using 1.8 kV applied immediately prior to the analytical column via a microtee built into the nanospray source with the ion transfer tube heated to 320 °C and the S-lens set to 60%. Precursor ions were measured in a data-dependent mode in the orbitrap analyzer at a resolution of 70,000 and a target value of 3e6 ions. The ten most intense ions from each MS1 scan were isolated, fragmented in the HCD cell, and measured in the orbitrap at a resolution of 17,500. The mass spectrometry proteomic data have been deposited on the ProteomeXchange via the PRIDE partner repository with the dataset identifier PXD034142 (Reviewer account details: Username: reviewer_pxd034142@ebi.ac.uk, Password: gfukklaI).

### Processing of LTβR-SC AP-MS data

Raw data were analyzed with MaxQuant (version 1.6.17.0) where they were searched against the human SwissProt database (http://www.uniprot.org/, downloaded 06/11/2020) using default settings. Carbamidomethylation of cysteines was set as fixed modification, and oxidation of methionines and acetylation at protein N-termini were set as variable modifications. Enzyme specificity was set to trypsin with maximally 2 missed cleavages allowed. To ensure high confidence identifications, peptide-spectral matches, peptides, and proteins were filtered at a less than 1% false discovery rate (FDR). Label-free quantification in MaxQuant was used with a LFQ minimum ratio count of 2, Fast LFQ selected and the ‘skip normalization’ option selected [91]. The ‘match between runs’ feature was deselected. For the LTβR-SC discovery experiment, log2 LFQ protein intensity values from each replicate were averaged. For the experiment comparing LTβR-SC in Hep3B cells either proficient or deficient in HOIP, quantified proteins were analyzed within the model-based statistical framework MSstats [92] (version 3.20.0, run through RStudio (version 1.2.5042, R version 4.0.0)). Data were log2 transformed, quantile normalized, and a linear mixed-effects model was fitted to the data. The group quantification function was used to obtain model-based protein abundance across biological replicates [93]. To identify specific proteins associated with theLTβR-SC, the following filtering steps were applied. First, proteins quantified in unstimulated controls either in Hep3B WT or Hep3B HOIP KO cells were regarded as contaminants and filtered out. Second, proteins detected in more than 15% of 716 experiments in the Contaminant Repository for Affinity Purification (CRAPome) were filtered out [94]. CRAPome is introduced as a valuable resource for evaluating protein-protein interactions. It offers a comprehensive database and computational tools at www.crapome.org. This resource helps researchers score protein interactions more accurately by providing an extensive background dataset, allowing them to filter out potential contaminants from the beads used in affinity purification coupled with mass spectrometry. Finally, proteins associated with the LTβR-SC together with LUBAC and its previously reported interactors within other signaling complexes [44, 55, 95, 96] were highlighted graphically. The list of proteins with their relative intensities can be found in the Supplementary Table 1 and X. In Fig. 1D, proteins were functionally associated using the stringApp (v2.0.1) [97] via Cytoscape (v3.9.1) [98, 99].

### Immunohistochemistry and immunofluorescence staining

Original data obtained from hepatocellular carcinoma patients was obtained from patients from the Tri-Service General Hospital, National Defense Medical Centre, Taiwan. Ethical issues regarding human rights and specimen management were approved by the Institutional Review Board (IRB) of the Tri-Service General Hospital. The registration number is TSGH-IRB No. 1-107-05-023. The patients’ information regarding all clinical characteristics were summarized in Supplementary Table 4. Resected livers were fixed in 10% formalin and embedded in paraffin blocks and cut into sections of 4 μm in thickness. For antigen retrieval, samples were subjected to heat-induced epitope retrieval with citrate buffer (10 mM sodium citrate, pH 6.0). Immunohistochemistry staining was performed by BOND-III Fully Automated IHC and ISH Staining System. For immunofluorescence staining of A549 WT and A549 LTβR KO cells, culture medium was removed followed by fixation with 4% Formaldehyde. PBS containing 10% normal serum with 0.1% Triton X-100 was used for blocking unspecific binding. Next, samples were incubated subsequently with primary antibodies followed by fluorophore-conjugated secondary antibodies. Nuclear counterstain was performed with DAPI. Images were obtained by confocal microscopy (Zeiss LSM900). For both stainings, anti-LTβR polyclonal antibodies were purchased from Proteintech (20331-1-AP).

### Public dataset retrieved and analysis

Normalized RNA-seq data from different cancer types was obtained from the publicly available database TCGA (The Cancer Genomic Atlas) and GTEx (The Genotype-Tissue Expression). According to this database, all data was obtained by using the Illumina HiSeq platform and retrieved by the bioinformatics tool UCSC Xena browser (URL: https://xenabrowser.net/). The phenotypic cohort including survival data and clinical parameters were also retrieved from the patients diagnosed of colon cancer (COAD), stomach (STAD), sarcoma (SARC), pancreatic cancer (PAAD), ovarian cancer (OV), hepatocellular carcinoma(LIHC), renal papillary cell carcinoma(KIRP), renal clear cell carcinoma(KIRC), head and neck cancer(HNSC), esophageal cancer (ESCA), cervical cancer (CESC), breast cancer(BRCA), bladder cancer (BLCA), rectal cancer(READ), and lung cancer(LUAD/LUSC). Samples without detailed information in terms of normalized mRNA expression, survival status and overall times were removed before enrollment. The clinical characteristics regarding the patients with high LTβR/RNF31 expression and high LTβR/low RNF31 expression were summarized as Supplementary Table 5.

### Statistics

GraphPad Prism 9 was used for data analysis. Two-sided unpaired t-test was used for evaluating the statistical significance between groups as indicated. For Survival analysis, Log-rank test was used for assessing the survival difference between different groups. For loss of viability data, values are demonstrated as mean percentage of loss of viability ± SD for 3–4 independent experiments as specified for each panel in the legends. *P*-values < 0.05 are indicated and considered as statistically significant.

## Supplementary information

Supplementary figures

Supplementary Table 1

Supplementary Table 2

Supplementary Table 3

Supplementary Table 4

Supplementary Table 5

Original uncropped blots

## Data Availability

Mass spectrometry proteomic data is deposited on the ProteomeXchange via the PRIDE partner repository with the dataset identifier PXD034142. Additionally, all data is available upon request.
