# Supplementary material for: LUBAC enables tumor-promoting LTβ receptor signaling by activating canonical NF-κB
Source: Cell Death Differ. 2024 Aug 30;31(10):1267–84. doi: 10.1038/s41418-024-01355-w (PMC11445442; doi:10.1038/s41418-024-01355-w)

Supplementary Figures

Figure S1. The activation of LTβR is required for the secretion of proinflammatory chemokines.

(A) Surface expression of the receptors LTβR and HVEM was assessed on the indicated cell lines. For each cell line, both the isotype control and the specific staining for LTβR or HVEM are shown. (B) Hep3B, HLE, HSC3, and JHH4 cells were stimulated with TAP-LIGHT (200 ng/ml) for the indicated time points followed by total protein extraction. Cell lysates were subjected to Western blot analysis and probed for the indicated proteins. Representative results of at least three independent replicates are shown. (C) JHH4 were stimulated with TAP-LIGHT or TAP-TNF for 24 hours. Etanercept/Enbrel (50 µg/ml) was added 1 hour before ligand-induced stimulation. Supernatants were collected and levels of IL8 and CCL20 were quantified by ELISA. Error bars represent the mean +/-SEM of at least three independent experiments. (D) HLE and JHH4 cells were stimulated with increasing concentrations of LT-α1β2 in the presence of 1% FCS for 24 hours. Supernatants were collected and levels of IL8 and CCL20 were quantified by ELISA. Error bars represent the mean +/-SEM of at least three independent experiments. (E) Assessment of LTβR expression in LTβR KO cells was performed by LTβR surface staining and Western blotting (top panels) and functional assessment of the effect of LTβR absence on TNF- and LIGHT-induced IL-8 secretion (bottom panels). WT or LTβR KO cells were stimulated with TAP-TNF (250 ng/ml) or the indicated concentrations of TAP-LIGHT, and levels of IL8 were quantified by ELISA. Error bars represent the mean +/-SEM of at least three independent experiments. (F) A549, JHH4 and HLE were stimulated with TAP-LIGHT (500 ng/ml) and co-incubated with inhibitors of TAK1 ((5Z)-7-Oxozeaenol (“7-ox”, 10µM)), IKKα/β (TPCA (5µM)) or NIK (B022 (25µM)). All inhibitors were incubated 1 hour before adding TAP-LIGHT. Supernatants were collected and levels of IL-8 were quantified by ELISA. Error bars represent the mean +/-SEM of at least three independent experiments.

Figure S1

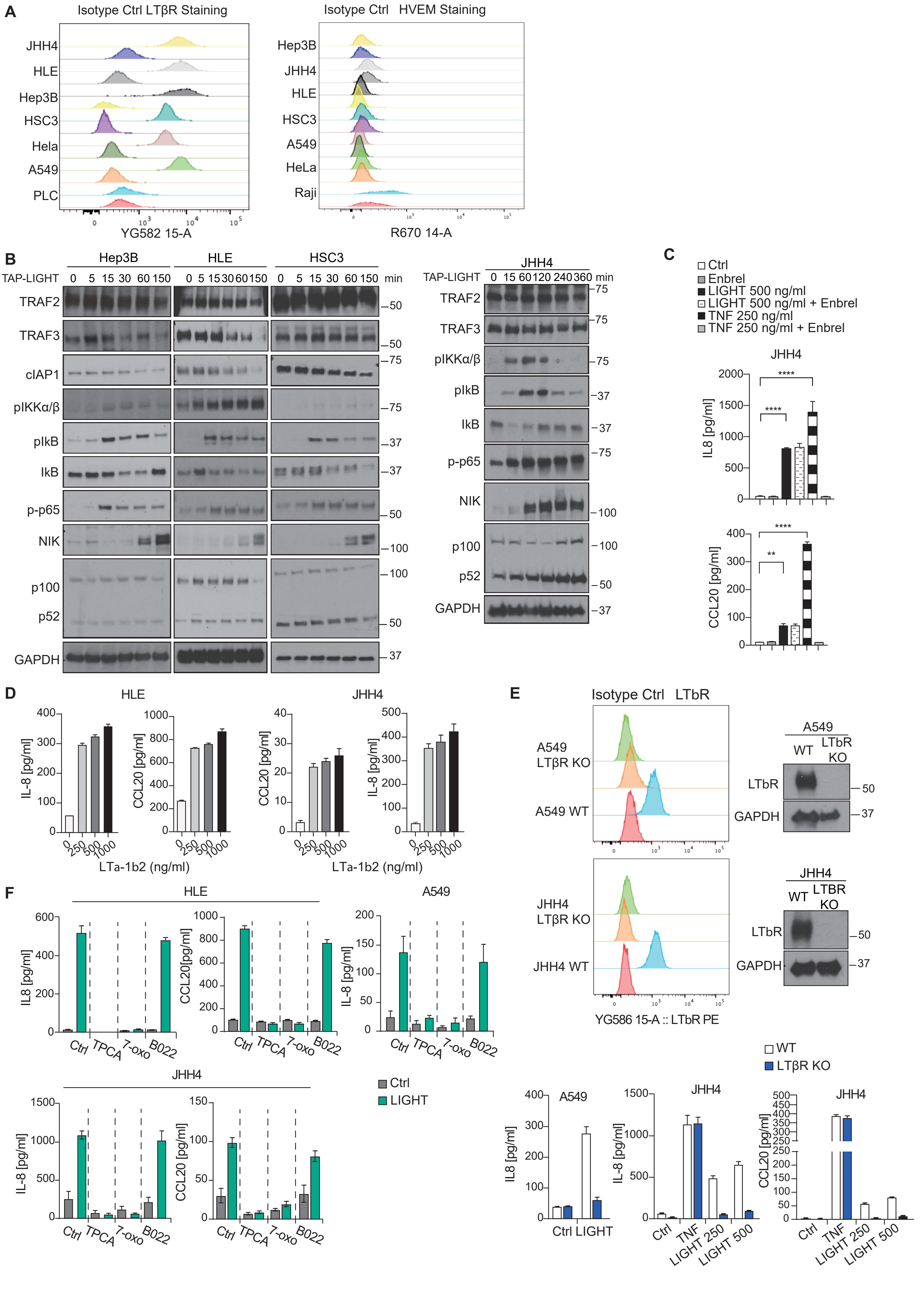
A
Isotype Ctrl LTβR Staining
JHH4
HLE
Hep3B
HSC3
Hela
A549
PLC
YG582 15-A
Isotype Ctrl HVEM Staining
Hep3B
JHH4
HLE
HSC3
A549
HeLa
Raji
R670 14-A
B
Hep3B
HLE
HSC3
TAP-LIGHT 0 5 15 30 60 150 min
TRAF2
TRAF3
cIAP1
pIKKα/β
pIkB
IkB
p-p65
NIK
p100
p52
GAPDH
JHH4
TAP-LIGHT 0 15 60 120 240 360 min
C
Ctrl
Enbrel
LIGHT 500 ng/ml
LIGHT 500 ng/ml + Enbrel
TNF 250 ng/ml
TNF 250 ng/ml + Enbrel
JHH4
IL8 [pg/ml]
CCL20 [pg/ml]
D
HLE
JHH4
IL-8 [pg/ml]
CCL20 [pg/ml]
LTa-1b2 (ng/ml)
E
Isotype Ctrl LTbR
A549 LTβR KO
A549 WT
JHH4 LTβR KO
JHH4 WT
YG586 15-A :: LTbR PE
A549
WT
LTbR KO
LTbR
GAPDH
JHH4
LTBR KO
F
HLE
A549
JHH4
IL8 [pg/ml]
CCL20[pg/ml]
IL-8 [pg/ml]
Ctrl TPCA 7-oxo B022
Ctrl
LIGHT
WT
LTβR KO
Ctrl LIGHT
TNF
LIGHT 250
LIGHT 500

Figure S2. LUBAC deficiency does not sensitize to cell death upon engagement of LTβR.

(A, E, F) The indicated cells were incubated with medium alone (Ctrl), medium with 20% FCS or different concentrations of LIGHT with 10% FCS for 24hrs. Cell viability was determined by CellTiter-Glo. Error bars represent the mean +/-SEM of at least three independent experiments. (B, C, D) A549 (B) or Hep3B (C,D) cells were stimulated with the indicated concentrations of TAP-LIGHT, iz-TRAIL, or TAP-TNF in the presence or absence or SMAC mimetics, and a time-lapse analysis of cell death by Incucyte was performed using Sytox green positivity as the readout for cell death.

Figure S2

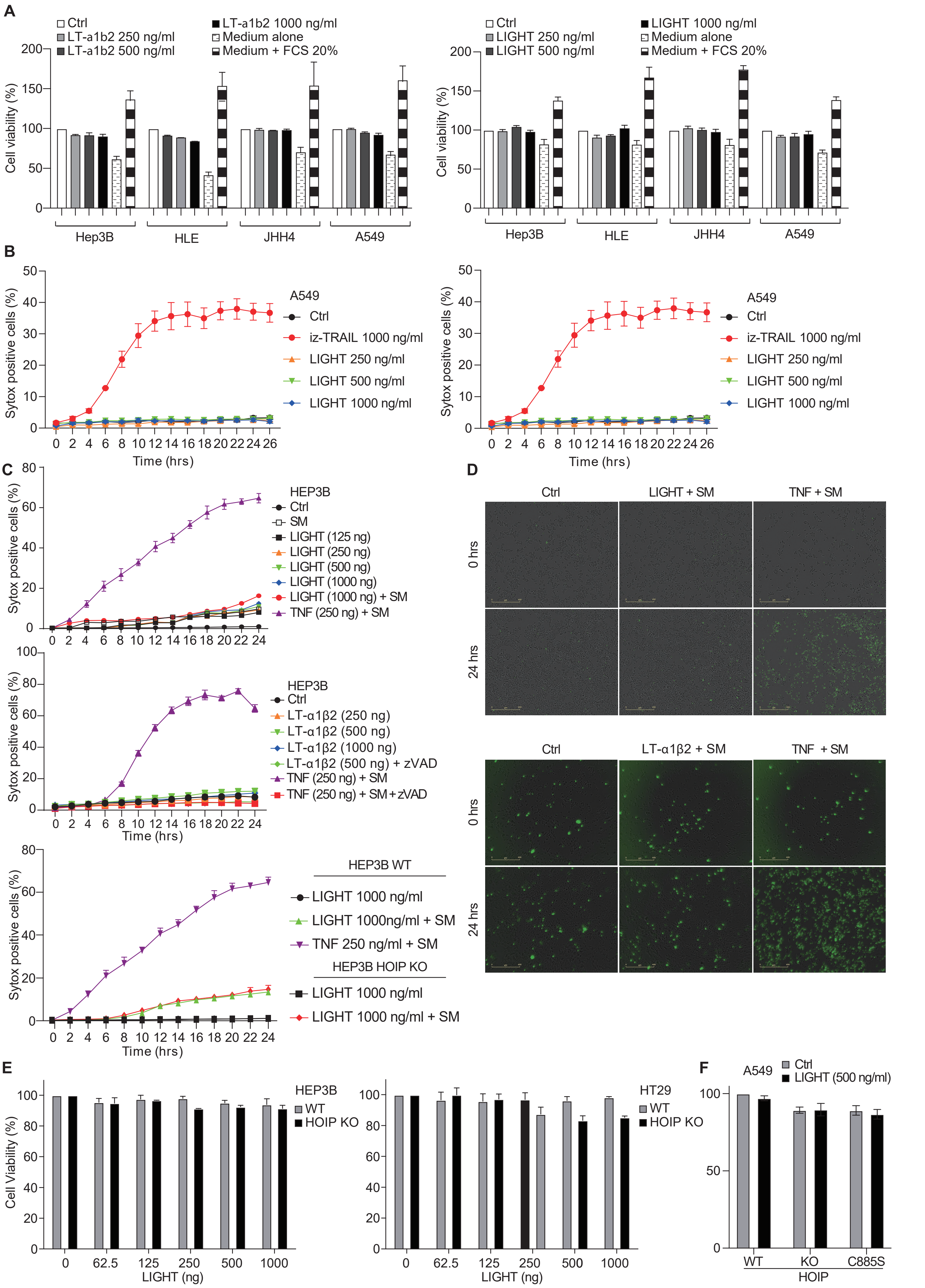

A
Ctrl
LT-a1b2 250 ng/ml
LT-a1b2 500 ng/ml
LT-a1b2 1000 ng/ml
Medium alone
Medium + FCS 20%
Cell viability (%)
Hep3B
HLE
JHH4
A549
LIGHT 250 ng/ml
LIGHT 500 ng/ml
LIGHT 1000 ng/ml
B
A549
iz-TRAIL 1000 ng/ml
Sytox positive cells (%)
Time (hrs)
C
HEP3B
SM
LIGHT (125 ng)
LIGHT (250 ng)
LIGHT (500 ng)
LIGHT (1000 ng)
LIGHT (1000 ng) + SM
TNF (250 ng) + SM
LT-α1β2 (250 ng)
LT-α1β2 (500 ng)
LT-α1β2 (1000 ng)
LT-α1β2 (500 ng) + zVAD
TNF (250 ng) + SM + zVAD
HEP3B WT
LIGHT 1000 ng/ml
LIGHT 1000ng/ml + SM
TNF 250 ng/ml + SM
HEP3B HOIP KO
LIGHT 1000 ng/ml + SM
D
Ctrl
LIGHT + SM
TNF + SM
0 hrs
24 hrs
LT-α1β2 + SM
E
Cell Viability (%)
HEP3B
WT
HOIP KO
HT29
LIGHT (ng)
F
A549
LIGHT (500 ng/ml)
WT
KO
C885S
HOIP

Figure S3. TRAF2, TRAF3 and NEMO, but not NIK or LTβR, are linearly ubiquitinated upon LIGHT stimulation.

Hep3B cells were treated with TAP-LIGHT (2000 ng/ml) for 30 mins and lyzed in denaturing conditions. Linear ubiquitin affinity immunoprecipitation (M1-AP) was performed, and samples were analyzed by Western blot.

Figure S3 (related to Figure 3)

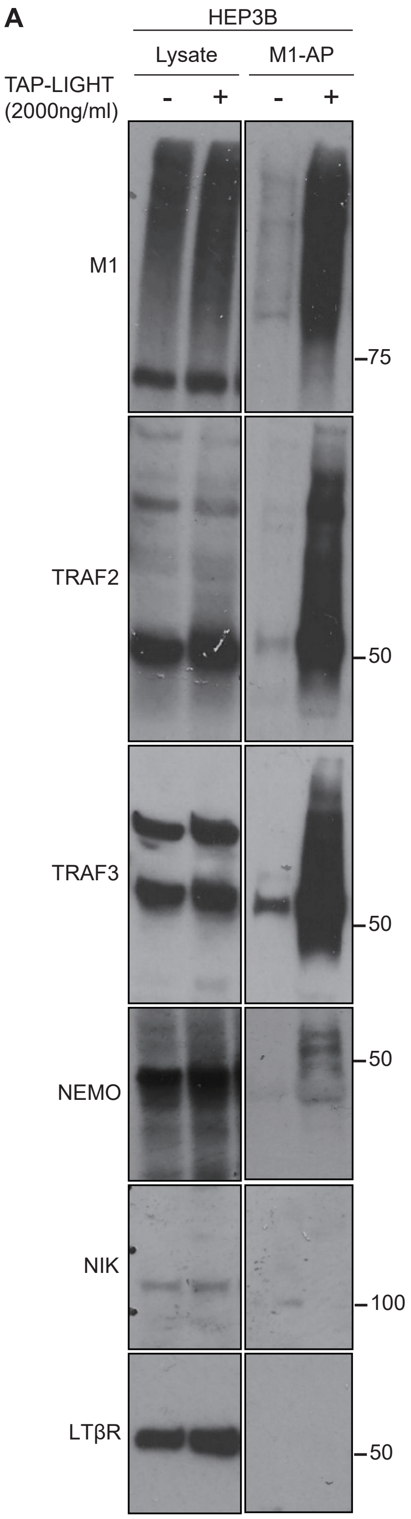

Figure S4. Linear ubiquitin binding capacity of A20 is required for its recruitment to LTβR-SC.

(A) A549 control cells or A549 cells lacking zinc finger 7 of A20 (A20-ΔZnF7) were stimulated with TAP-LIGHT (2000 ng/ml) for the indicated times followed by total protein extraction. Native LTβR-SC was isolated by using M2-beads and analyzed by Western blotting. Representative results of at least three independent replicates are shown. (B) IL8 concentration was analyzed by ELISA. Error bars represent the mean +/-SEM of experiments performed in triplicate. (C) A549 WT, A49 A20 KO, and A20-ΔZnF7 were stimulated with TAP-LIGHT (500 ng/ml) for 24 hours. Cell viability was measure via Cell Titer Glow.

Figure S4 (related to Figure 5B)

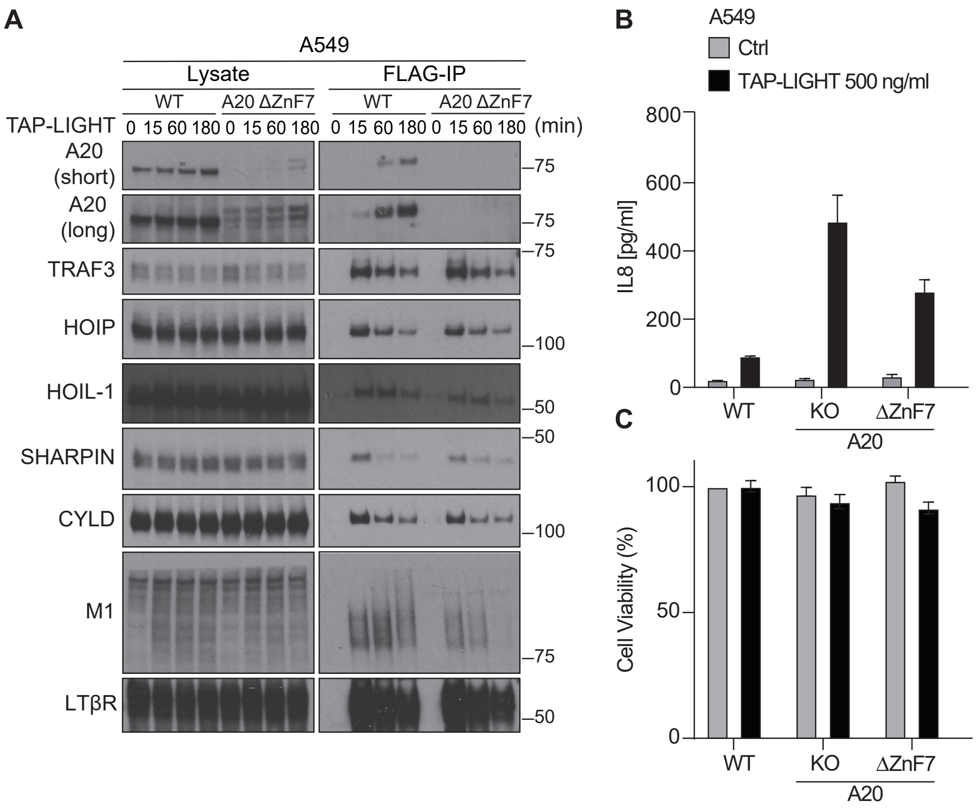

Figure S5. OPTN is required for optimal TBK1 activation, but is not indispensable for it.

(A, D, E) The indicated cells were stimulated with LIGHT (200 ng/ml) for the indicated times before cell lysis. The lysates were separated by SDS-PAGE and analyzed by Western blot. (B, C) A549 WT, A549 OPTN KO or A549 OPTN KO cells reconstituted with full length OPTN (B) or A549 NEMO KO cells (C) were stimulated with TAP-LIGHT (2000 ng/ml) for the indicated times followed by LTβR-SC purification, SDS-PAGE separation and Western blotting. (F) IL-8 levels were analyzed by ELISA. Error bars represent the mean +/-SEM of experiments performed in triplicate. Representative results of at least three independent replicates are shown.

Figure S5

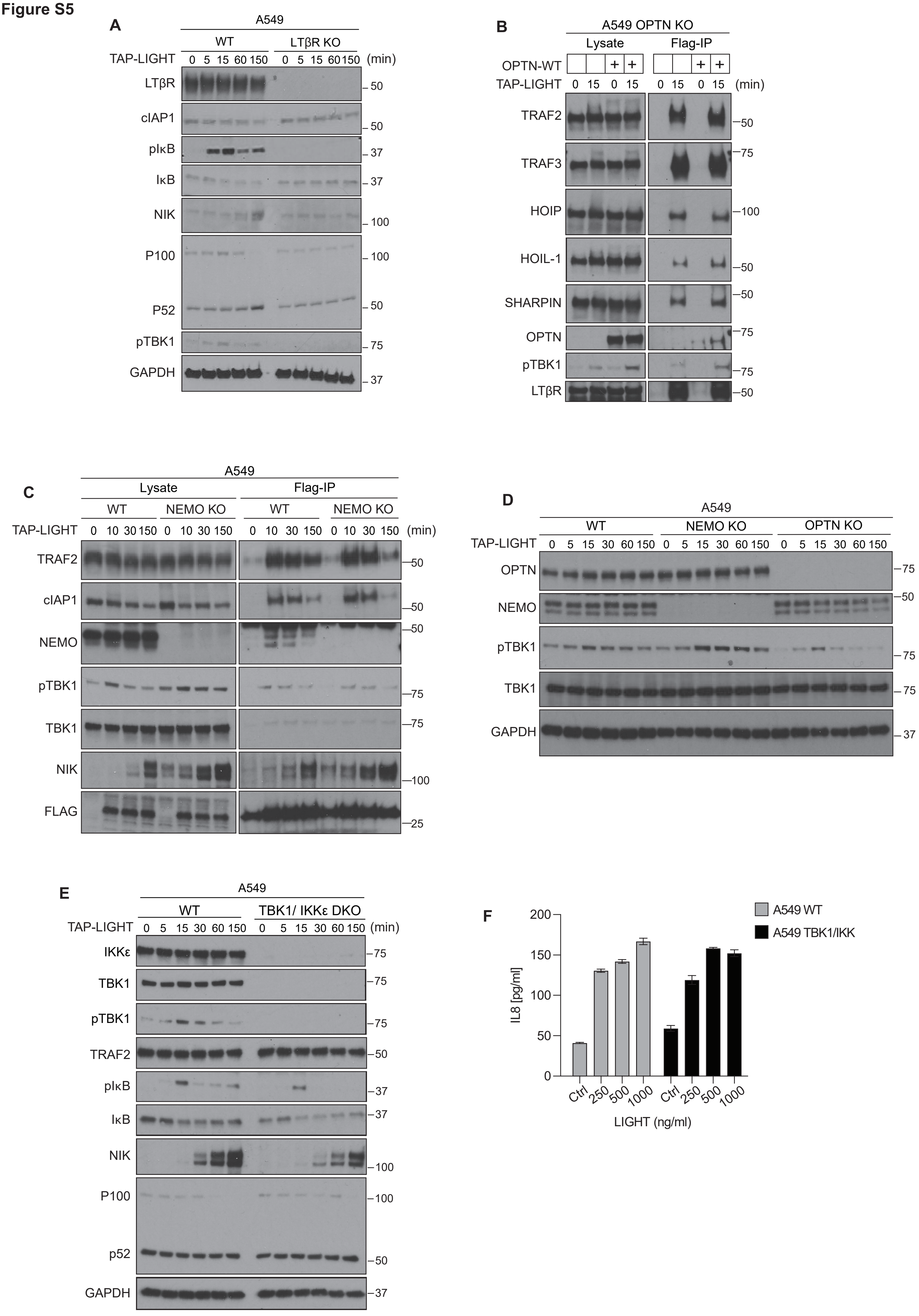
A
A549
WT
LTβR KO
TAP-LIGHT 0 5 15 60 150 0 5 15 60 150 (min)
LTβR
cIAP1
pIκB
IκB
NIK
P100
P52
pTBK1
GAPDH
50
50
37
37
100
100
50
75
37
B
A549 OPTN KO
Lysate
Flag-IP
OPTN-WT + + + +
TAP-LIGHT 0 15 0 15 0 15 0 15 (min)
TRAF2
TRAF3
HOIP
HOIL-1
SHARPIN
OPTN
pTBK1
LTβR
50
75
100
50
50
75
75
50
C
A549
Lysate
Flag-IP
WT
NEMO KO
WT
NEMO KO
TAP-LIGHT 0 10 30 150 0 10 30 150 0 10 30 150 0 10 30 150 (min)
TRAF2
cIAP1
NEMO
pTBK1
TBK1
NIK
FLAG
50
50
50
75
75
100
25
D
A549
WT
NEMO KO
OPTN KO
TAP-LIGHT 0 5 15 30 60 150 0 5 15 30 60 150 0 5 15 30 60 150
OPTN
NEMO
pTBK1
TBK1
GAPDH
75
50
75
75
37
E
A549
WT
TBK1/ IKKε DKO
TAP-LIGHT 0 5 15 30 60 150 0 5 15 30 60 150 (min)
IKKε
TBK1
pTBK1
TRAF2
pIκB
IκB
NIK
P100
p52
GAPDH
75
75
75
50
37
37
100
100
50
37
F
A549 WT
A549 TBK1/IKK
IL8 [pg/ml]
200
150
100
50
0
Ctrl
250
500
1000
Ctrl
250
500
1000
LIGHT (ng/ml)

Figure S6. The LTβR-mediated signaling output correlates with decreased survival in various cancer entities.

(A) Forest plot showing the correlation between high expression of LTβR and overall survival of patients suffering from different types of cancer. Raw data of mRNA transcripts among different TCGA databases was normalized by log_2FPKM, which was downloaded from UCSC XENA. The median value of mRNA transcripts was set up as a cut-off value for separating high expression and low expression LTβR groups among different databases. (B) Assessment of LTβR expression in A549 WT and A549 LTβR-KO cells by confocal microscopy. (C) Correlation matrix of the cyto-/chemokines and LTβR retrieved from the TCGA LIHC dataset. The correlation coefficient between the expression values of two different genes was assessed by the strength of Spearman's rho correlation coefficient. Color intensity as well as the size of the circles represent the degree of correlation between two given cyto-/chemokines. Stars indicate statistical significance between correlation coefficients. Significance was tested using a Spearman rank test and level of significance was set at $p < 0.01$. (D) Kaplan–Meier survival analysis depicting the difference of overall survival between the group with high level transcripts of IL-8 or CCL20, and the group with lower level transcripts of these genes in liver cancer patients. Data obtained from the TCGA LIHC database. (E) Survival map retrieved from GEPIA 2 showing the contribution of different genes related to proinflammatory or cell death pathways with overall survival. Data used was obtained from TCGA LIHC and LUAD datasets. A bold border of the indicated genes indicates statistical significance in their overall survival. Separation between high expression and low expression groups was performed by using the median expressional value of the indicated gene. The hazard ratio was assessed by cox regression hazard model via GEPIA-2 interactive website (http://gepia2.cancer-pku.cn/#index). The statistical significance was calculated using the cox-regression hazard model, and level of significance was set at $p < 0.05$.

Figure S6 (related to Figure 6)

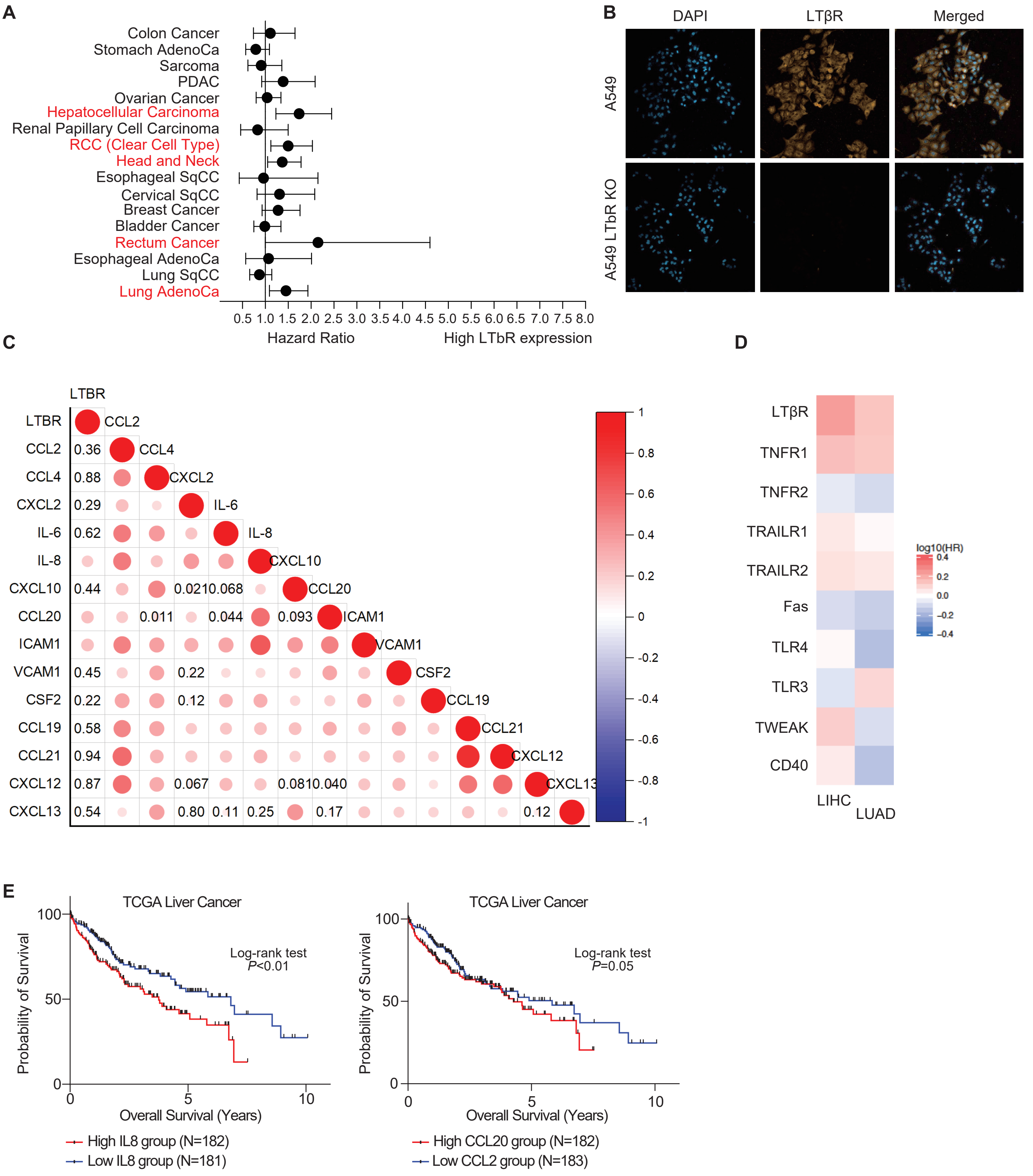

Figure S7. LTβR signaling in liver cancer may primarily affect the tumor cells themselves rather than the immune cells.

Immune cell subsets in our patient populations of interest were analyzed using CIBERSORT. (A) A bar chart displays the proportion of immune cell subsets, with the X-axis showing the indicated cases and the Y-axis showing the percentage of 22 immune cell types. The infiltrating immune cells were compared in high LTβR expression HCC patients, stratified into high- or low-HOIP expression groups. (B) The proportion of the indicated immune cell types is compared between the high- and low-HOIP expression groups in high LTβR HCC patients. (C) In the high LTβR expression group, patients were further stratified into high or low RBCK (HOIL-1) expression groups based on quartile values, and survival results were represented in Kaplan-Meier survival

Figure S7 (related to Figure 6)

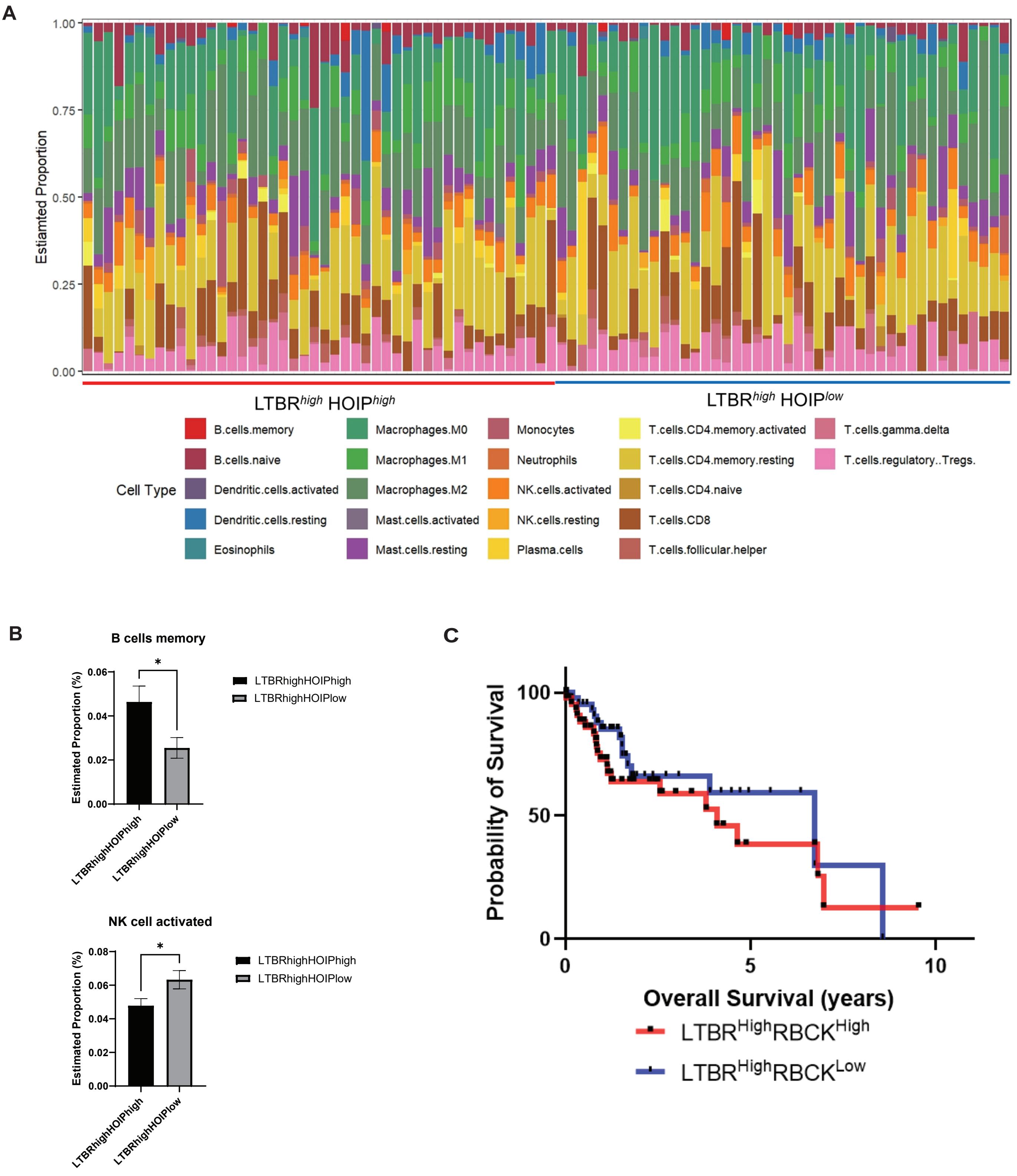

Supplement: Supplementary file 1 — Supplementary figures [file 41418_2024_1355_MOESM1_ESM.pdf]
