# Supplementary material for: LUBAC enables tumor-promoting LTβ receptor signaling by activating canonical NF-κB
Source: Cell Death Differ. 2024 Aug 30;31(10):1267–84. doi: 10.1038/s41418-024-01355-w (PMC11445442; doi:10.1038/s41418-024-01355-w)
Supplement: Supplementary file 4 — Supplementary Table 3 [file 41418_2024_1355_MOESM4_ESM.docx]

Supplementary Table 3: Key source Table

1. Antibodies

The following primary antibodies used for Western Blot were listed as below.

| **Antibody** | **Manufacturer** | **Catalogue number** | **Dilution** |
| --- | --- | --- | --- |
| TRAF2 | Enzo | ADI-AAP-422-E | 1:1000 |
| TRAF3 | Santa Cruz | sc-6933 | 1:1000 |
| c-IAP1 (D5G9) | CST | 7065S | 1:1000 |
| Phospho IKK α and β (S176/180) | CST | 2697S | 1:500 |
| IkBa | CST | 9242S | 1:1000 |
| Phospho-IκBα (Ser32/36) (5A5) | CST | 9246S | 1:1000 |
| Phospho-NF-κB p65 (Ser536) | CST | 3033S | 1:2000 |
| NIK Antibody | CST | 4994S | 1:1000 |
| NF-κB2 p100/p52 (18D10) | CST | 3017S | 1:1000 |
| Phospho-SAPK/JNK (Thr183/Tyr185) (98F2) | CST | 4671S | 1:500 |
| Phospho-p38 MAPK (Thr180/Tyr182) (3D7) | CST | 9215S | 1:1000 |
| Phospho-TBK1 (S172) (D52C2) | CST | 5483P | 1:1000 |
| TBK1/NAK | CST | 3013S | 1:1000 |
| HOIP (human; full length) | Ubiquigent | 68-0013-100 | 1:1000 |
| HOIL-1 (human / Mice) | Home made | NA | 1:1000 |
| Linear ubiquitin | Home made | NA | 1:1000 |
| Sharpin | Proteintech | 14626-I-AP | 1:2000 |
| CYLD | Santa Cruz | sc-74435 | 1:1000 |
| A20/TNFAIP3 (D13H3) | CST | 5630 | 1:1000 |
| Optineurin(D2L8S) | CST | 58981 | 1:1000 |
| IKKg (NEMO) | CST | 2685 | 1:1000  1:500 in IP |
| Anti-FLAG | Sigma | F1804-200UG | 1:4000 |
| GAPDH | Abcam | ab8245 | 1:4000 |
| Lymphtoxin beta receptor | Proteintech | PA5-75283 | 1:1000 |

Conjugated secondary antibodies

| **Antibody** | **Manufacturer** | **Catalogue number** |
| --- | --- | --- |
| Rat Anti-Mouse IgG2a-HRP | South biotech | 115505 |
| Goat Anti-Mouse IgG1, HRP | South biotech | 107005 |
| Goat Anti-Rabbit IgG(H+L), HRP | South biotech | 405005 |
| Donkey Anti-Sheep IgG Antibody, HRP | Millipore | AP184P |

| **Antibody** | **Manufacturer** | **Catalogue number** | **Dilution** |
| --- | --- | --- | --- |
| PE anti-human Lymphotoxin beta receptor | Biolegend | 322008 | 1:100 |
| PE anti-mouse IgG2b Antibody | Biolegend | 406707 | 1:100 |
| APC anti-human CD270 (HVEM, TR2) Antibody | Biolegend | 318807 | 1:100 |
| APC Mouse IgG1, κ Isotype Ctrl Antibody | Biolegend | 400119 | 1:100 |

The following primary antibodies used for FACS staining were listed

1. Chemicals, Peptides, and Recombinant Proteins

| **Items** | **Manufacturer** | **Catalogue number** |
| --- | --- | --- |
| iz-TRAIL | Ganten et al., 2006 | NA |
| moTAP-TNF | Haas et al.,2011 | NA |
| Fc block | BioLegend | Cat#422302 |
| TPCA-1 | Tocris Bioscience | Cat. No. 2559 |
| 7-oxozeanol | Tocris Bioscience | Cat. No. 3604 |
| B022 | MCE | Cat. No.: HY-120501 |

1. Critical Commercial Assays

| **Items** | **Manufacturer** | **Catalogue number** |
| --- | --- | --- |
| Human Cytokine Array kit | R&D Systems | Cat# ARY022B |
| Human CCL2/MCP-1 DuoSet ELISA | R&D Systems | Cat#DY279 |
| Human CXCL8/IL-8 DuoSet ELISA | R&D Systems | Cat#DY208 |
| Human CCL20/MIP-3 alpha DuoSet ELISA | R&D Systems | Cat#DY360 |
| CellTiter-Glo | Promega | Cat#G7572 |
| Cell proliferation assay kit | Millipore | Cat#2750 |
| RNeasy mini spin kit | QIAGEN | Cat#74104 |
| RevertAid First Strand cDNA synthesis kit | Thermo Fisher | Cat#K1632 |

1. Software and Algorithms

| **Software** | **Manufacturer** | **Catalogue number** |
| --- | --- | --- |
| MaxQuant version 1.4.1.2 | NA | NA |
| MSstats converter script | https://msstats.org/ | NA |
| Limma R package | https://bioconductor.org/packages/  release/bioc/html/limma.html | NA |
| Prism-GraphPad | https://www.graphpad.com/features | NA |
