# Supplementary material for: LUBAC enables tumor-promoting LTβ receptor signaling by activating canonical NF-κB
Source: Cell Death Differ. 2024 Aug 30;31(10):1267–84. doi: 10.1038/s41418-024-01355-w (PMC11445442; doi:10.1038/s41418-024-01355-w)
Supplement: Supplementary file 5 — Supplementary Table 4 [file 41418_2024_1355_MOESM5_ESM.doc]

|  | High LTβR N=7 | | Low LTβR  N=7 | *p-value* |
| --- | --- | --- | --- | --- |
| Age |  |  | | |
| (Yrs; Mean±SD) | 76.01±5.92 | | 71.2±10.01 | 0.33 |
| BMI ( Mean±SD) | 23.09±3.58 | | 24.43±3.90 | 0.51 |
| Gender |  | |  | 0.55 |
| Male | 4 | | 6 |  |
| Female | 3 | | 1 |  |
| cTNM stage (AJCC) |  | |  | 0.17 |
| I | 2 | | 5 |  |
| II | 3 | | 2 |  |
| IIIa | 2 | | 0 |  |
| BLCL stage |  | |  | 0.14 |
| A | 4 | | 7 |  |
| B | 1 | | 0 |  |
| C | 2 | | 0 |  |
| CTP score |  | |  |  |
| A | 7 | | 7 |  |
| Differentiation |  | |  | 0.07 |
| Grade I | 0 | | 2 |  |
| Grade II | 4 | | 5 |  |
| Grade III | 3 | | 0 |  |
| Distant Metastases |  | |  | 0.46 |
| Yes | 2 | | 0 |  |
| No | 5 | | 7 |  |
| Albumin |  | |  |  |
| Mean±SD | 3.85±0.54 | | 3.89±0.49 | 0.91 |
| Total bilirubin |  | |  |  |
| Mean±SD | 0.57±0.17 | | 0.61±0.36 | 0.78 |
| Platelet count (*10^3^; mean±SD) | 183.9±33.35 | | 152.9±32.55 | 0.10 |

Supplementary Table 4: Clinical features of patient samples
