# Supplementary material for: LUBAC enables tumor-promoting LTβ receptor signaling by activating canonical NF-κB
Source: Cell Death Differ. 2024 Aug 30;31(10):1267–84. doi: 10.1038/s41418-024-01355-w (PMC11445442; doi:10.1038/s41418-024-01355-w)
Supplement: Supplementary file 6 — Supplementary Table 5 [file 41418_2024_1355_MOESM6_ESM.doc]

| Supplementary Table 5: Clinical features of patient samples used for correlation of expression data with probability of survival | | | | |
| --- | --- | --- | --- | --- |
|  |  | |  |  |
|  | High LTβR  Low RNF31 N=45 | | High LTβR  Low RNF31 N=45 | *p-value* |
| Age |  |  | | |
| (Yrs; Mean±SD) | 59.31±14.13 | | 61.11±12.29 | 0.52 |
| BMI ( Mean±SD) | 25.78±17.51 | | 24.99±5.27 | 0.78 |
| Gender |  | |  | 0.27 |
| Male | 25 | | 31 |  |
| Female | 20 | | 14 |  |
| Mean LTβR expression | 4.72 | | 4.80 | 0.21 |
| Mean RNF31 expression | 2.25 | | 0.83 | <0.0001 |
| cTNM stage (AJCC) |  | |  | 0.71 |
| I | 20 | | 19 |  |
| II | 10 | | 13 |  |
| IIIa | 10 | | 10 |  |
| IIIb | 2 | | 0 |  |
| IIIc | 1 | | 1 |  |
| IV | 0 | | 1 |  |
| Missing | 2 | | 1 |  |
| CTP score |  | |  | 0.50 |
| A | 24(36.7%) | | 28(43.5%) |  |
| B | 4(63.3%) | | 2(56.5%) |  |
| Missing | 19 | | 15 |  |
| **Vascular invasion** |  | |  | 0.92 |
| None | 27 | | 26 |  |
| Micro | 12 | | 11 |  |
| Macro | 2 | | 2 |  |
| Missing | 4 | | 6 |  |
| **fibrosis_ishak_score** |  | |  | 0.36 |
| No Fibrosis | 10 | | 9 |  |
| Portal Fibrosis | 1 | | 0 |  |
| Fibrous Speta | 5 | | 5 |  |
| Nodular Formation and Incomplete Cirrhosis | 1 | | 1 |  |
| Established Cirrhosis | 9 | | 3 |  |
| Missing | 19 | | 27 |  |
| **Adjacent hepatic tissue**  **inflammation** |  | |  | 0.69 |
| None | 13 | | 14 |  |
| Mild | 14 | | 10 |  |
| Severe | 2 | | 1 |  |
| Missing | 16 | | 20 |  |
| Albumin |  | |  |  |
| Mean±SD | 3.931±0.98 | | 3.55±1.06 | 0.12 |
| Total bilirubin |  | |  |  |
| Mean±SD | 1.038±1.51 | | 0.84±0.42 | 0.45 |
| Platelet count (*10^3^; mean±SD) | 248.2±107.7 | | 225.6±86.87 | 0.33 |
