# Supplementary material for: LUBAC enables tumor-promoting LTβ receptor signaling by activating canonical NF-κB
Source: Cell Death Differ. 2024 Aug 30;31(10):1267–84. doi: 10.1038/s41418-024-01355-w (PMC11445442; doi:10.1038/s41418-024-01355-w)

# Original Blot

Figure 1A

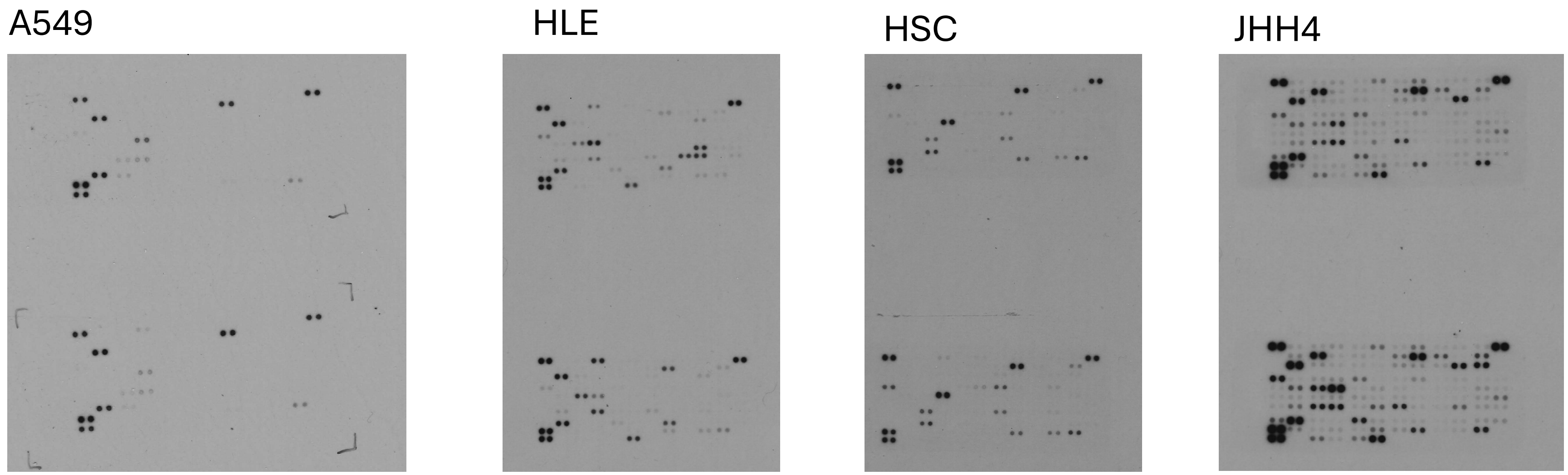

Figure 1E A549

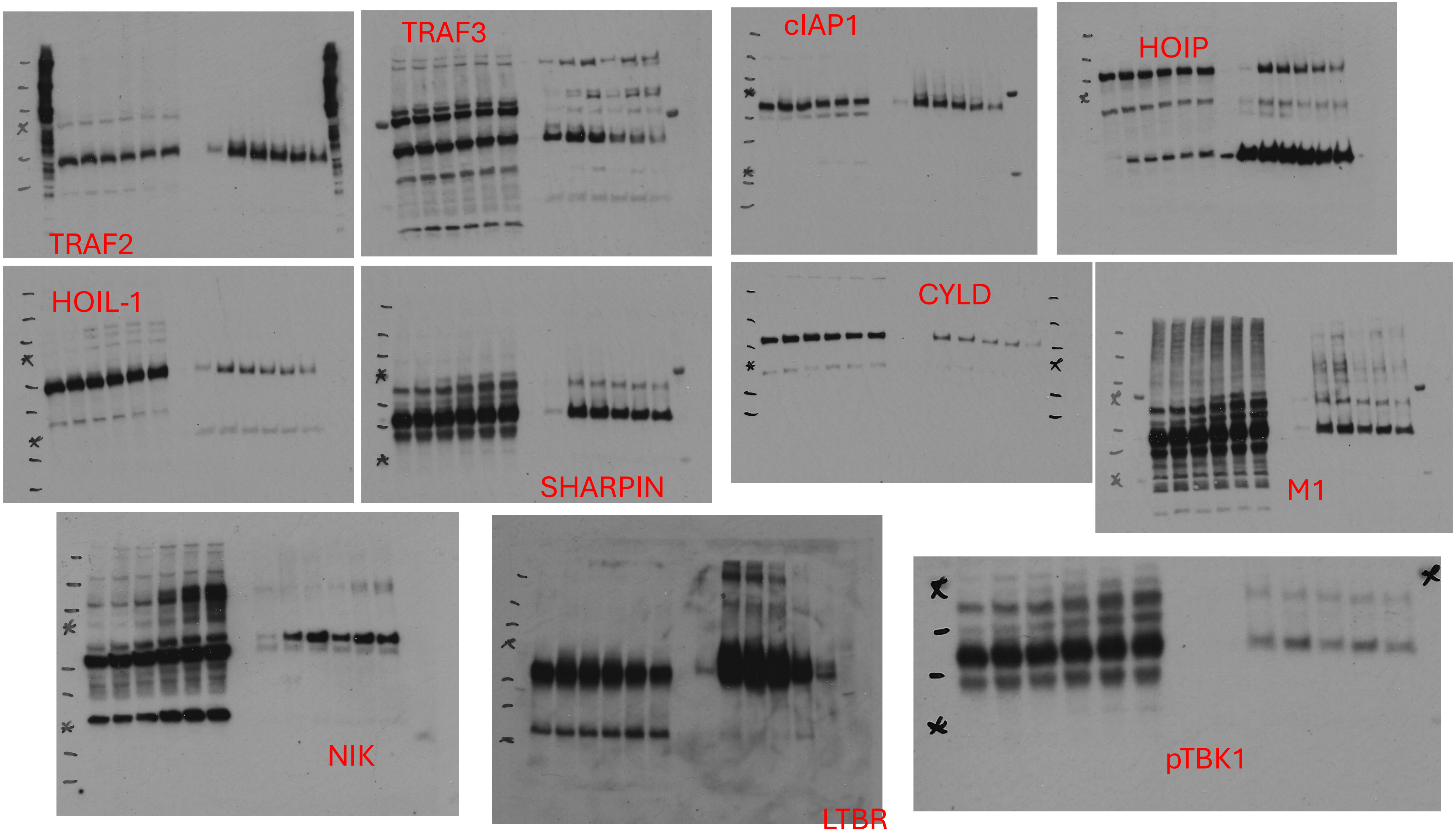

Figure 1E JHH4

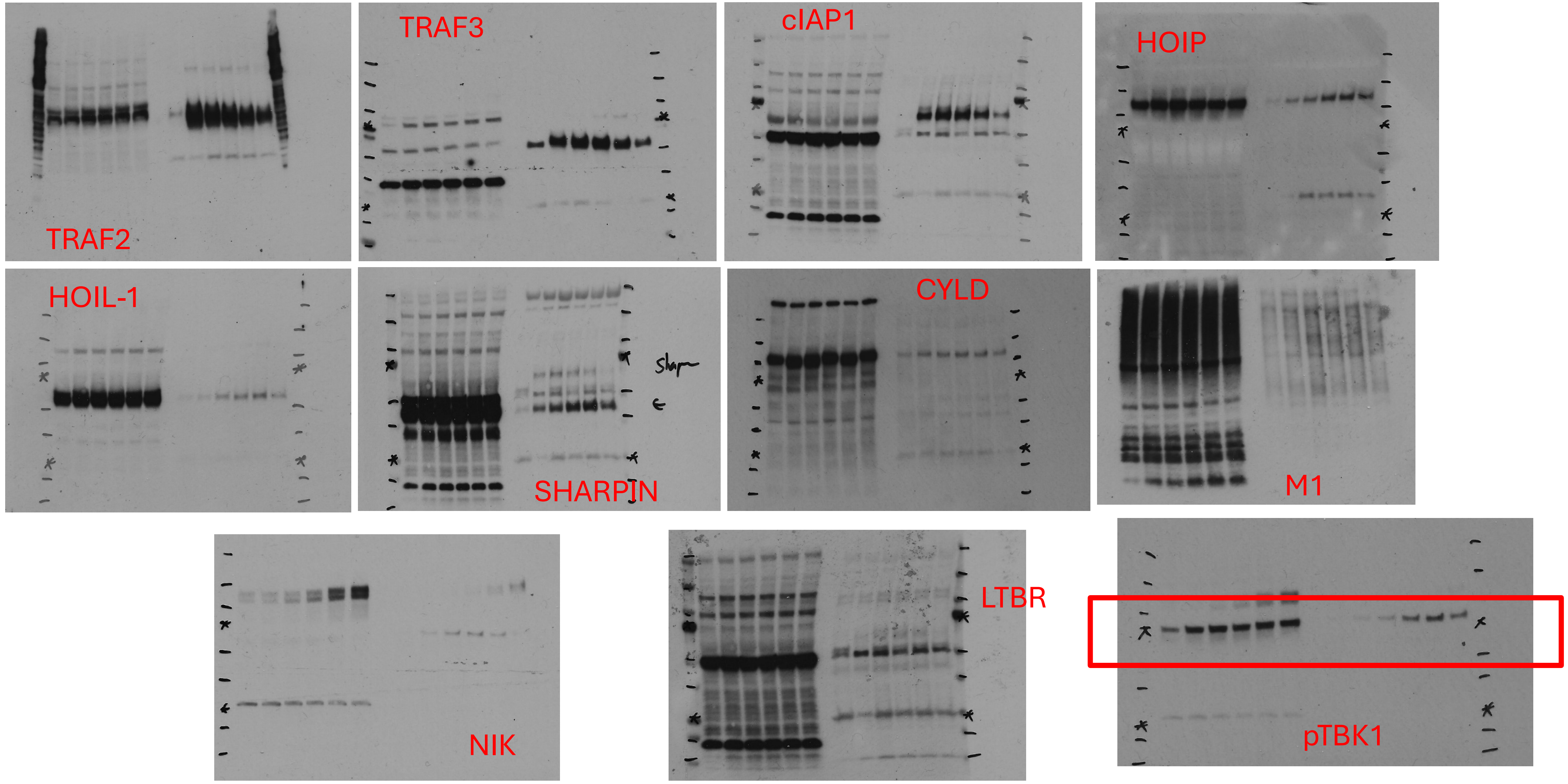

Figure 1E HLE

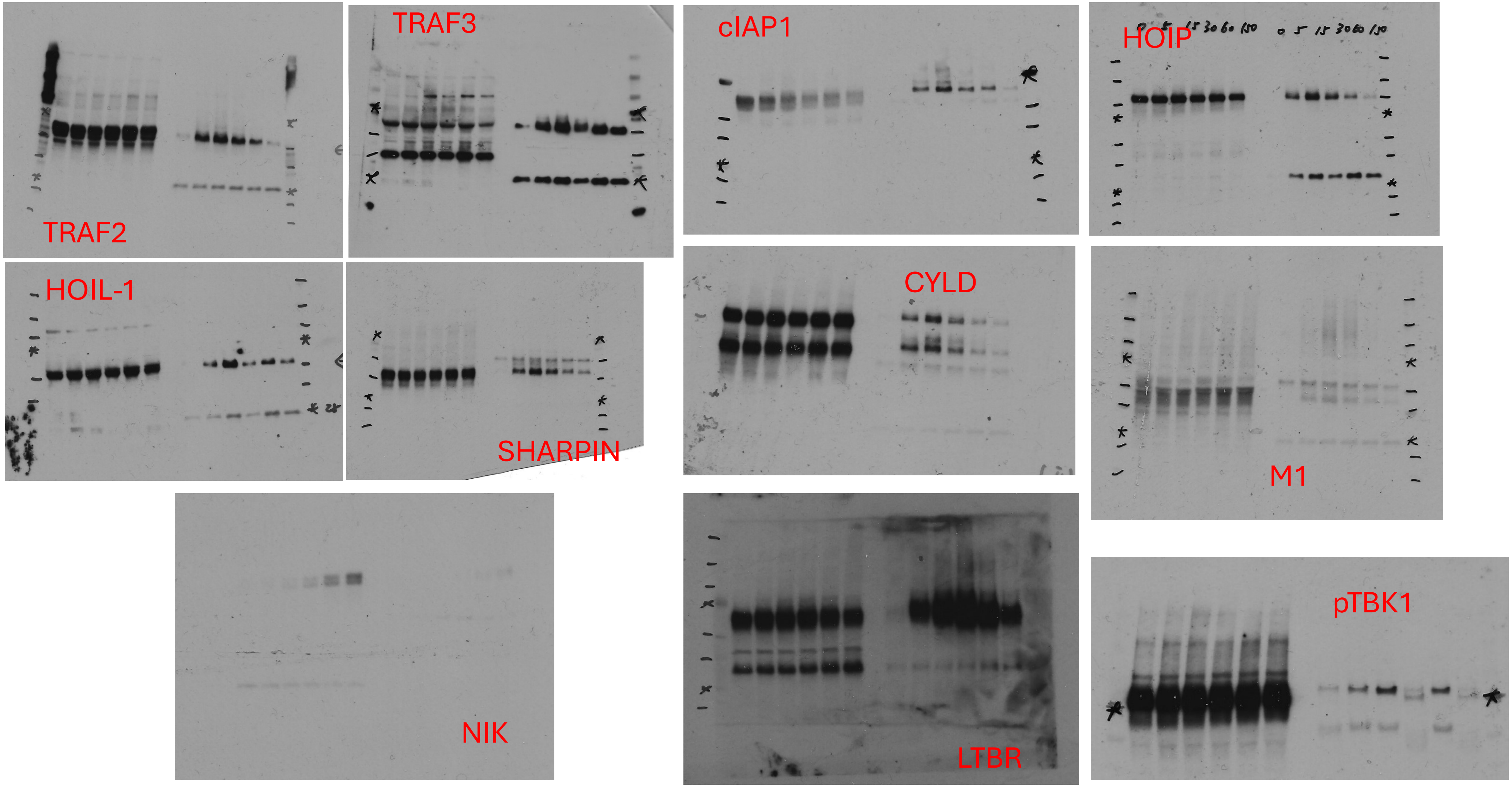

Figure 1E Hep3B

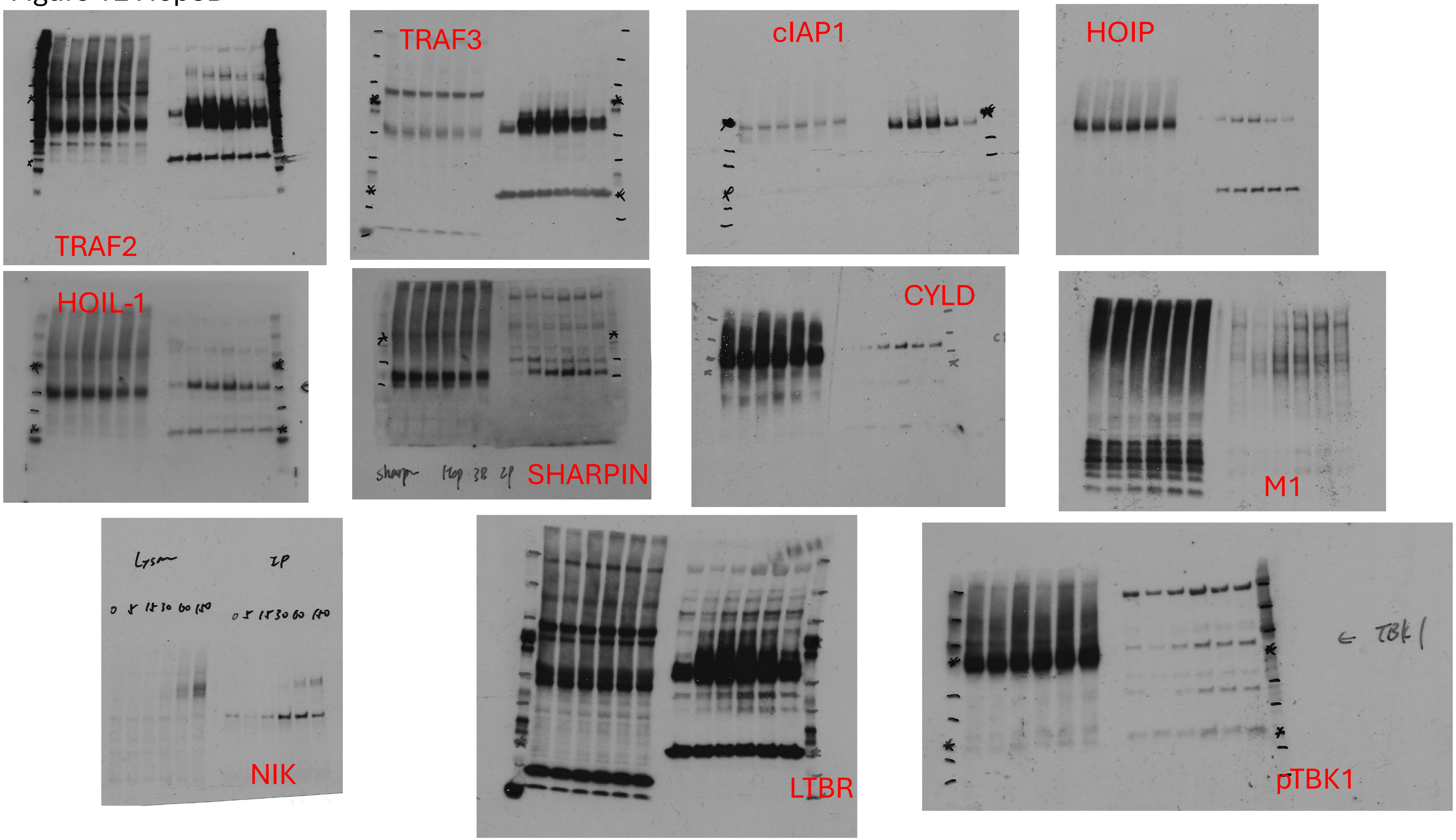

Figure 2A-1

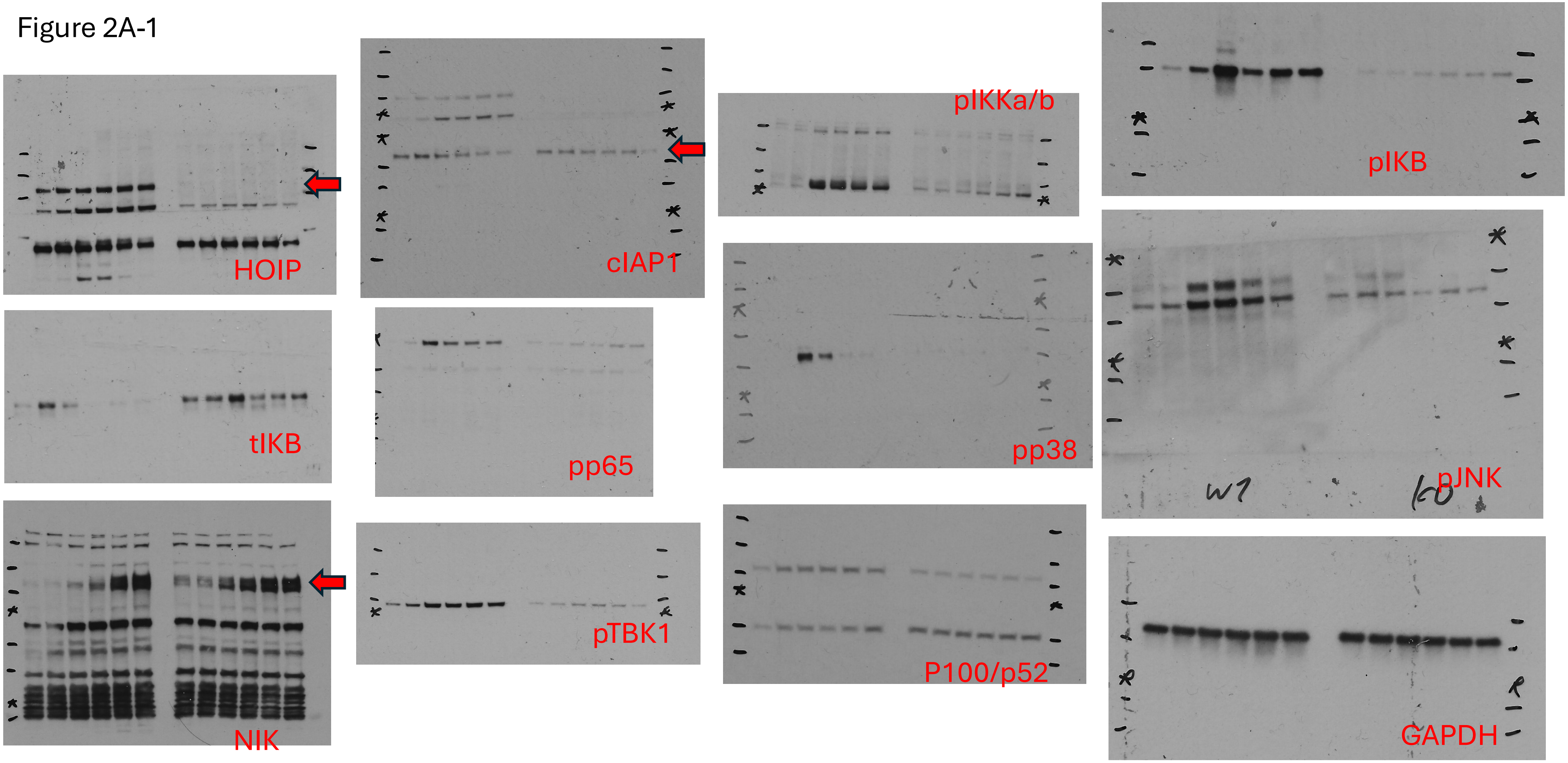
HOIP
cIAP1
pIKKa/b
pIKB
tIKB
pp65
pp38
pJNK
WT
KO
NIK
pTBK1
P100/p52
GAPDH

Figure 2A

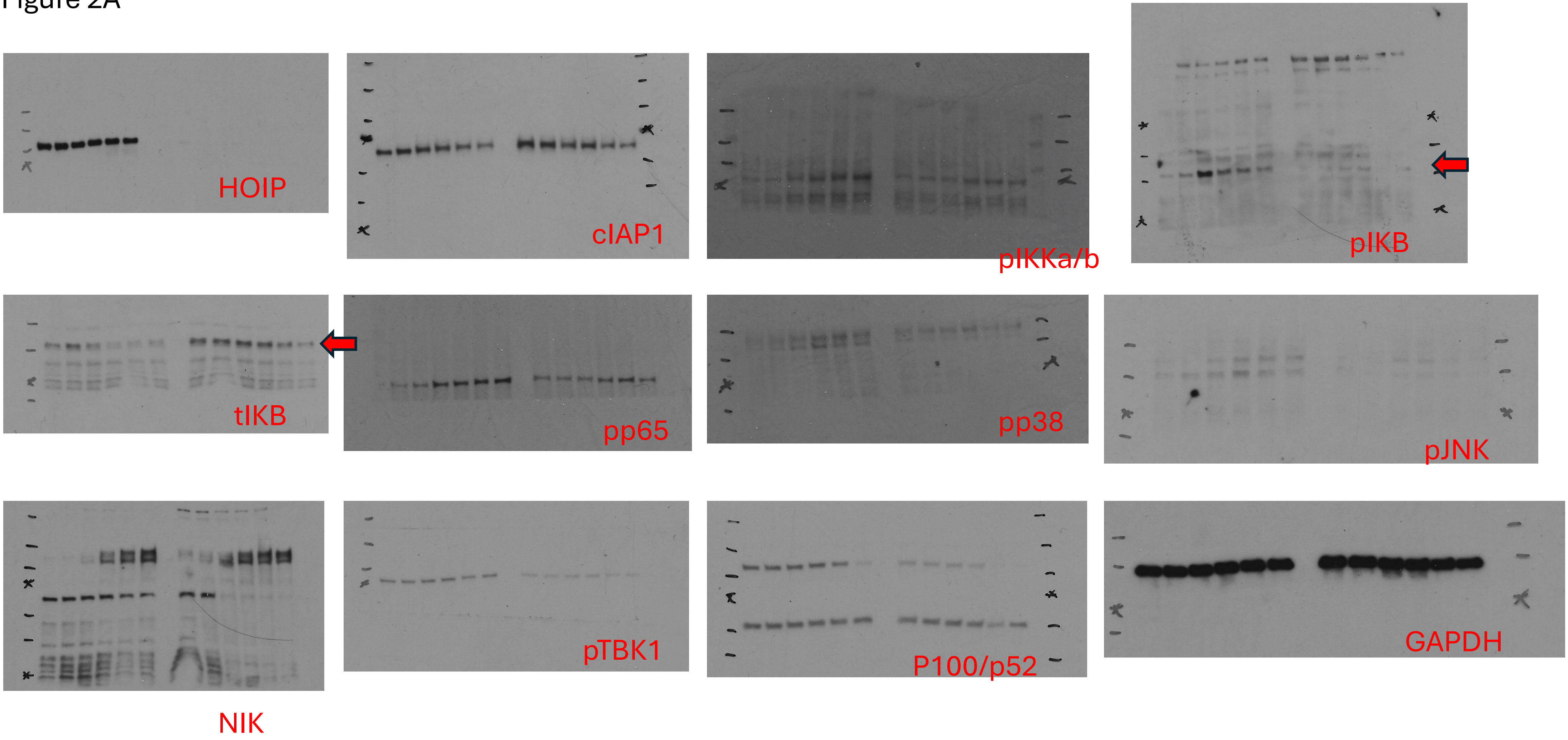

HOIP
cIAP1
pIKKa/b
pIKB
tIKB
pp65
pp38
pJNK
NIK
pTBK1
P100/p52
GAPDH

Figure 2A

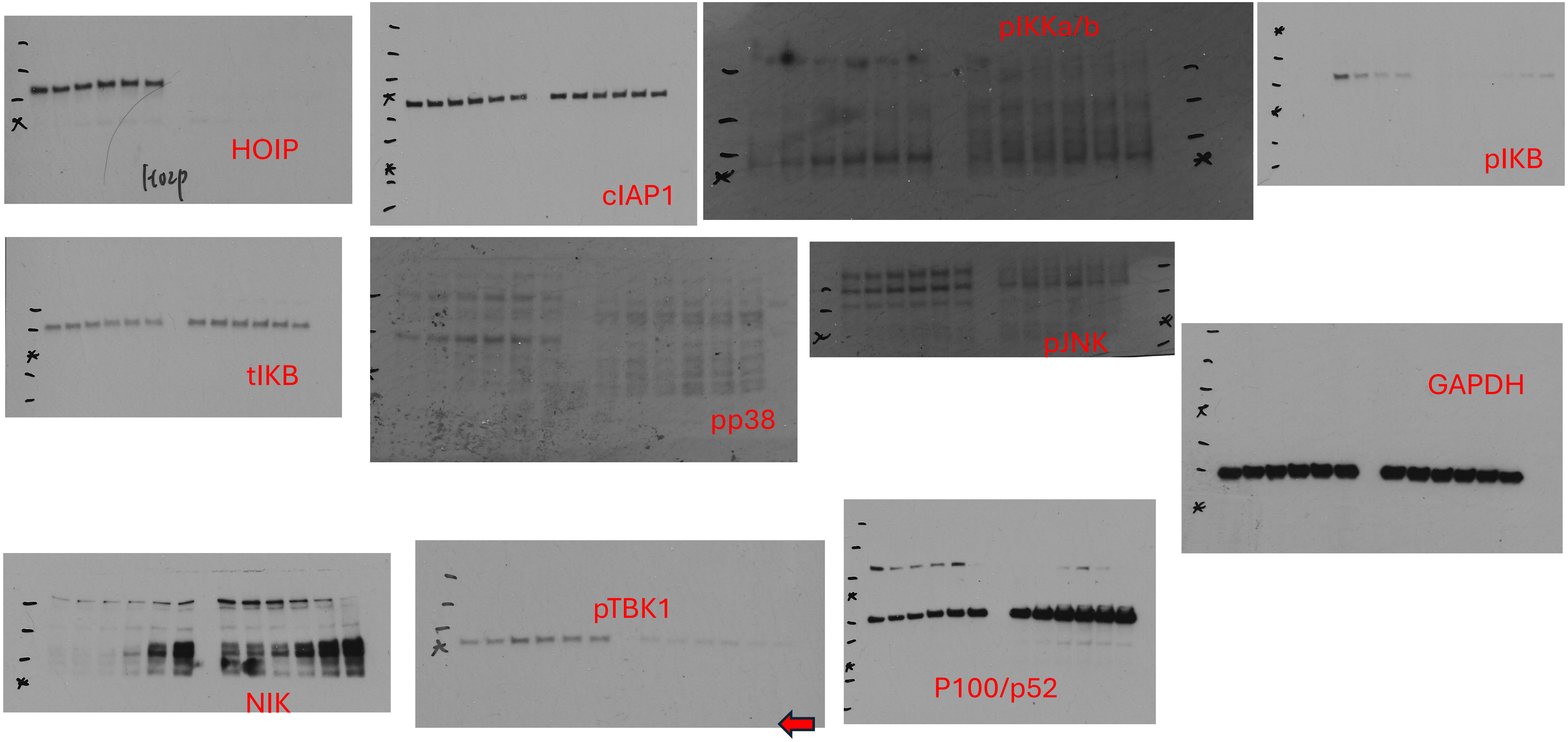
HOIP
cIAP1
pIKKa/b
pIKB
tIKB
pp38
pJNK
GAPDH
NIK
pTBK1
P100/p52

Figure 2E

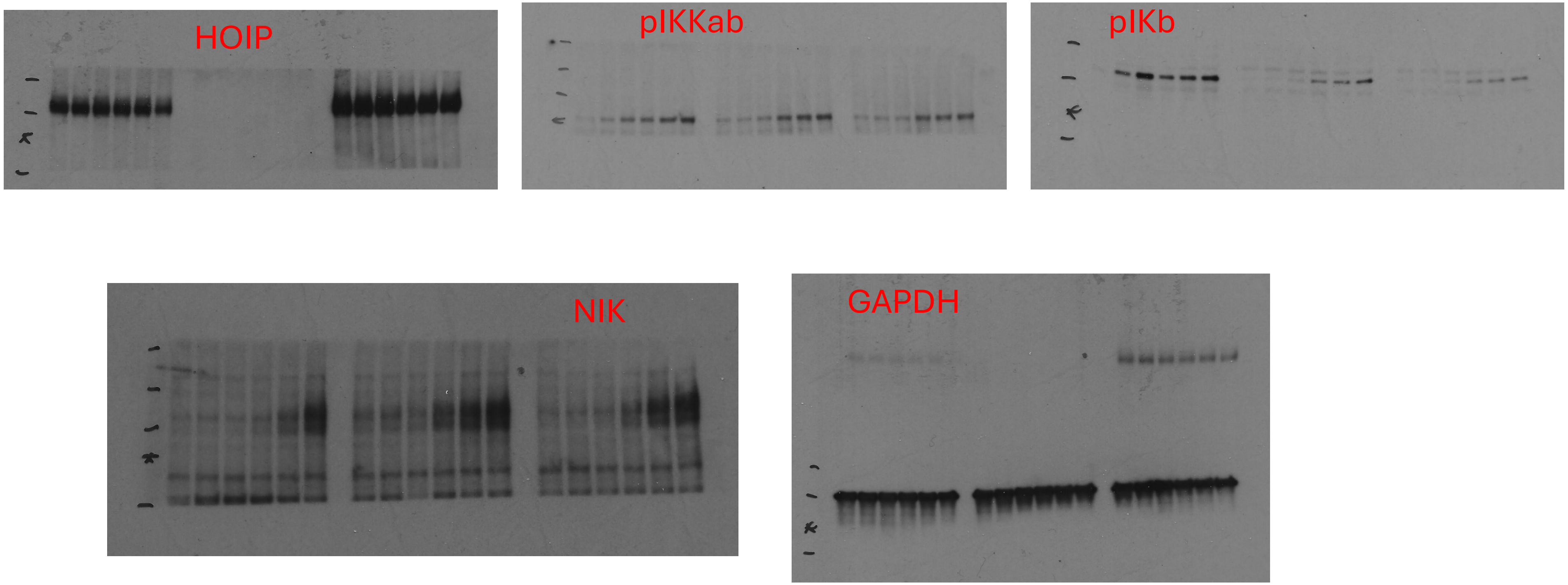

Figure 3C

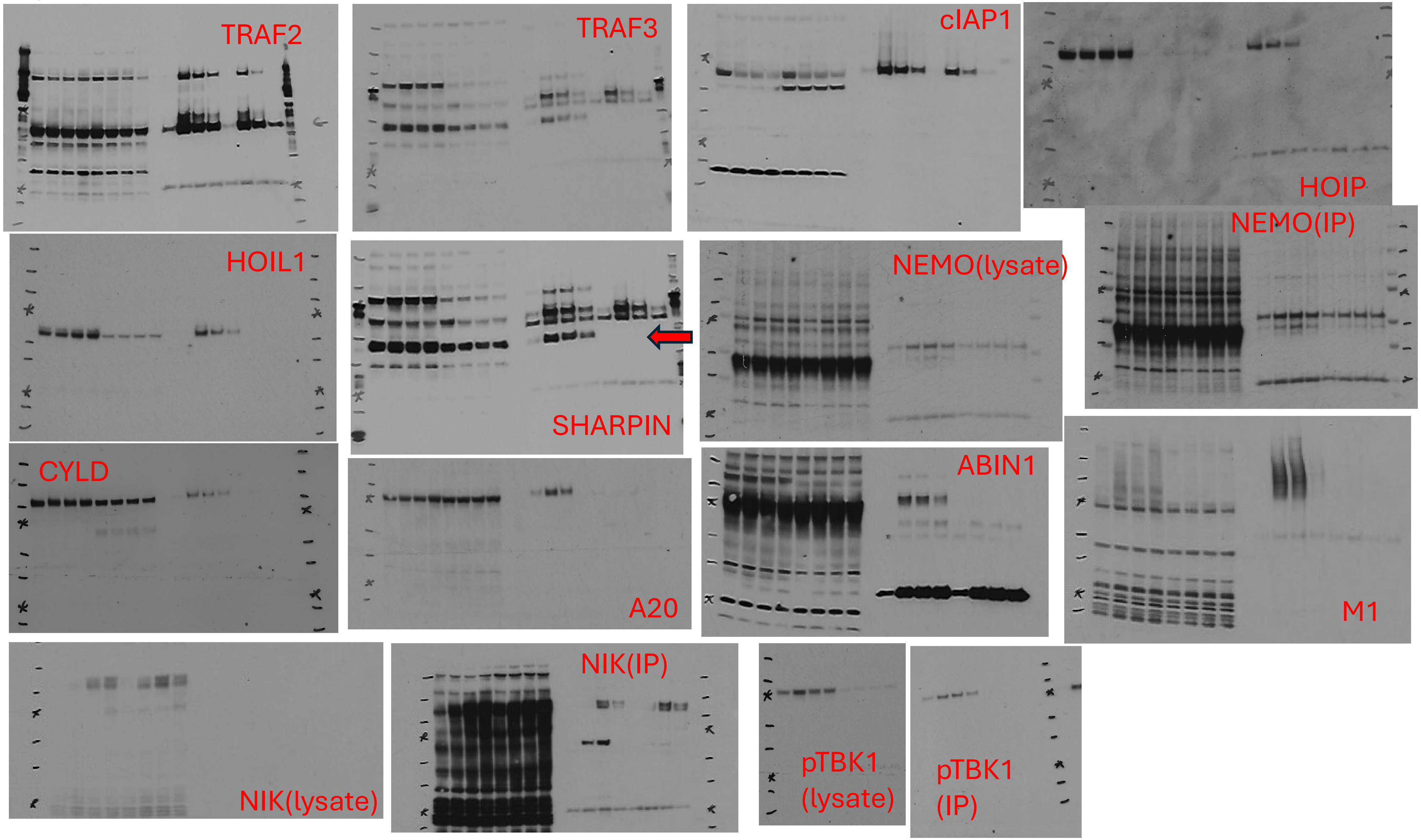
TRAF2
TRAF3
cIAP1
HOIP
NEMO(IP)
HOIL1
SHARPIN
NEMO(lysate)
CYLD
A20
ABIN1
M1
NIK(lysate)
NIK(IP)
pTBK1 (lysate)
pTBK1 (IP)

Figure 3D

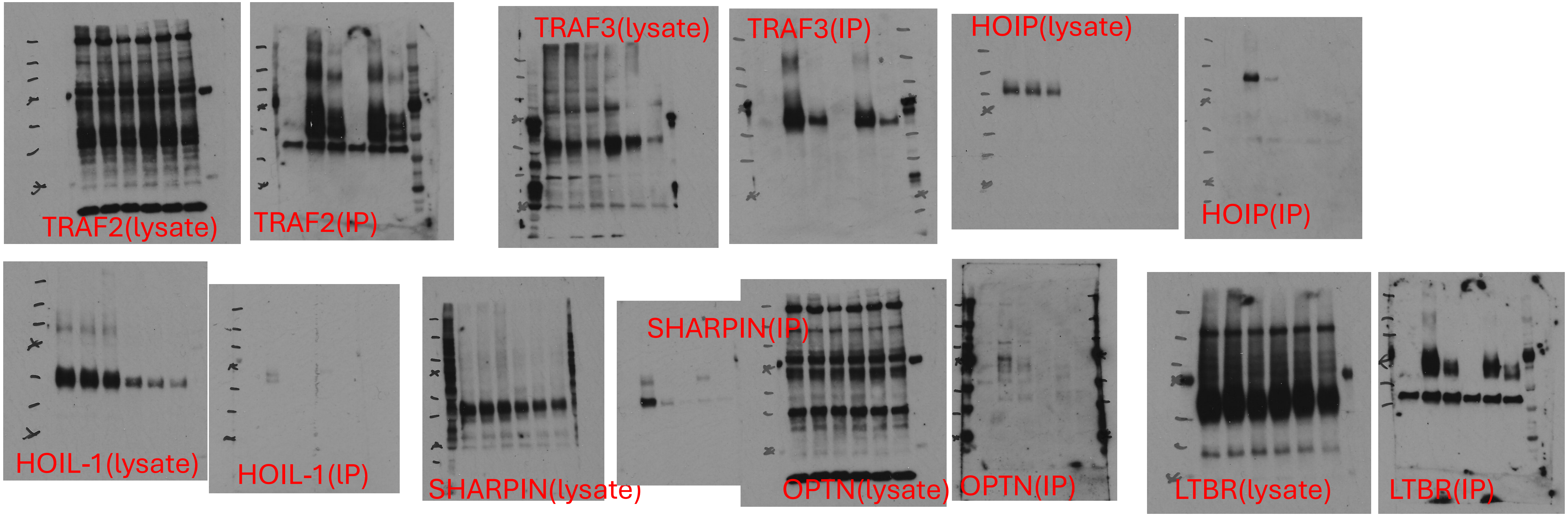
TRAF2(lysate)
TRAF2(IP)
TRAF3(lysate)
TRAF3(IP)
HOIP(lysate)
HOIP(IP)
HOIL-1(lysate)
HOIL-1(IP)
SHARPIN(lysate)
SHARPIN(IP)
OPTN(lysate)
OPTN(IP)
LTBR(lysate)
LTBR(IP)

Figure 3E

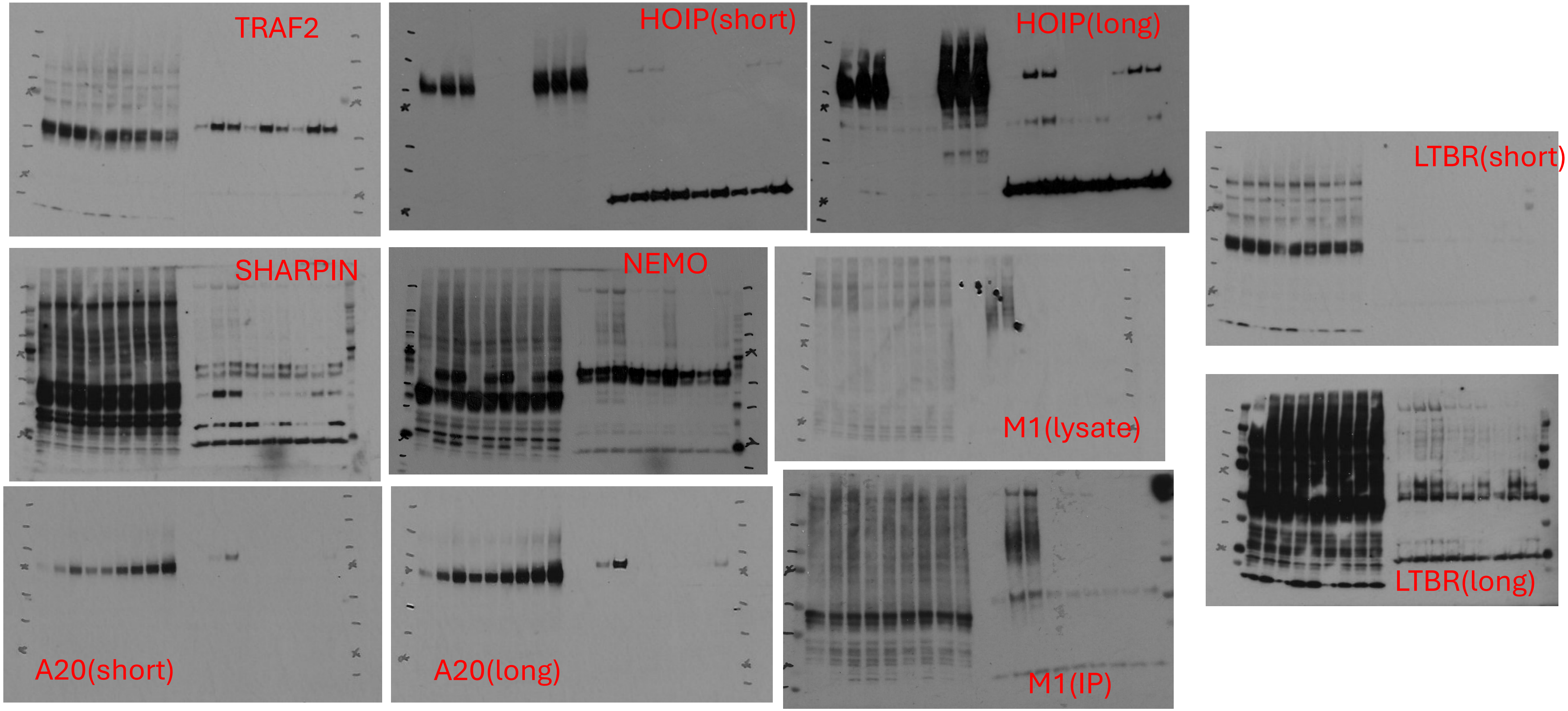
TRAF2
HOIP(short)
HOIP(long)
LTBR(short)
SHARPIN
NEMO
M1(lysate)
LTBR(long)
A20(short)
A20(long)
M1(IP)

Figure 4A

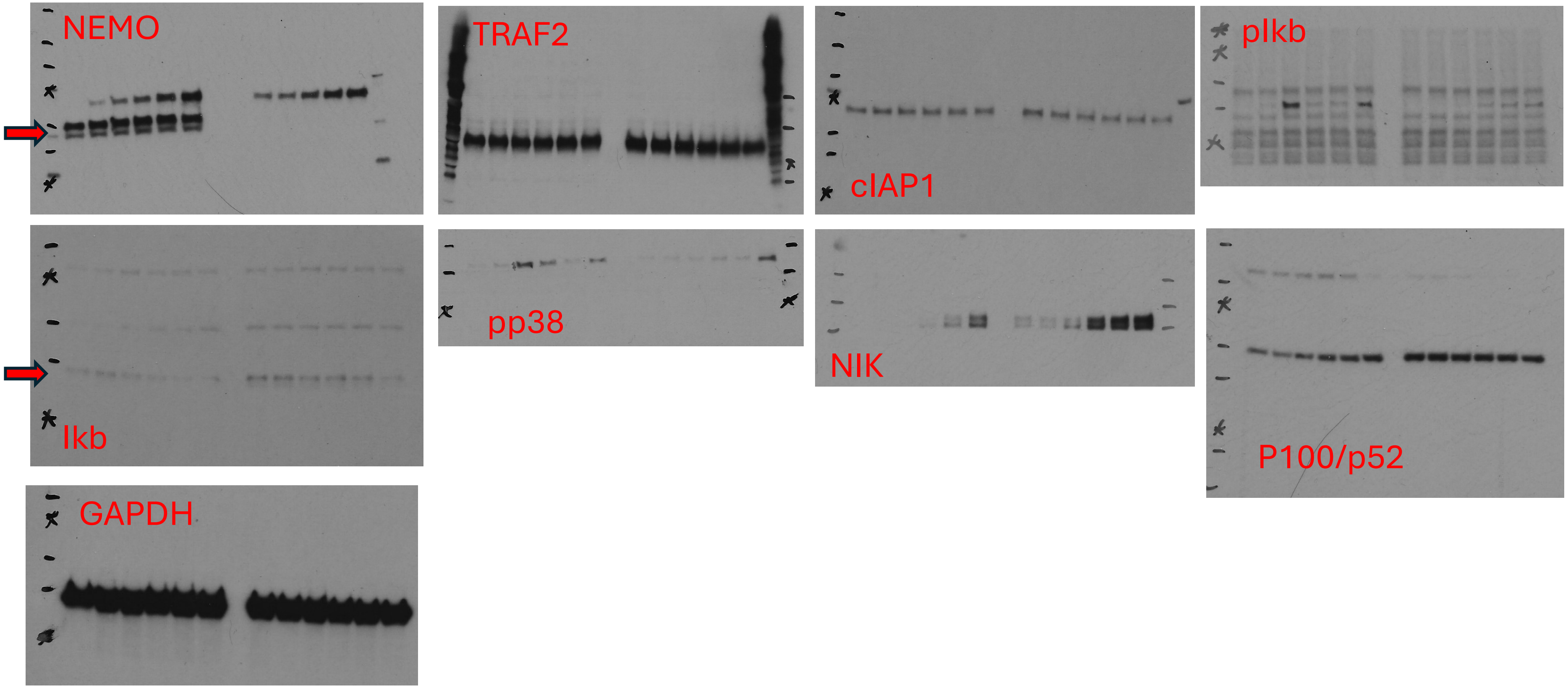

Figure 4B

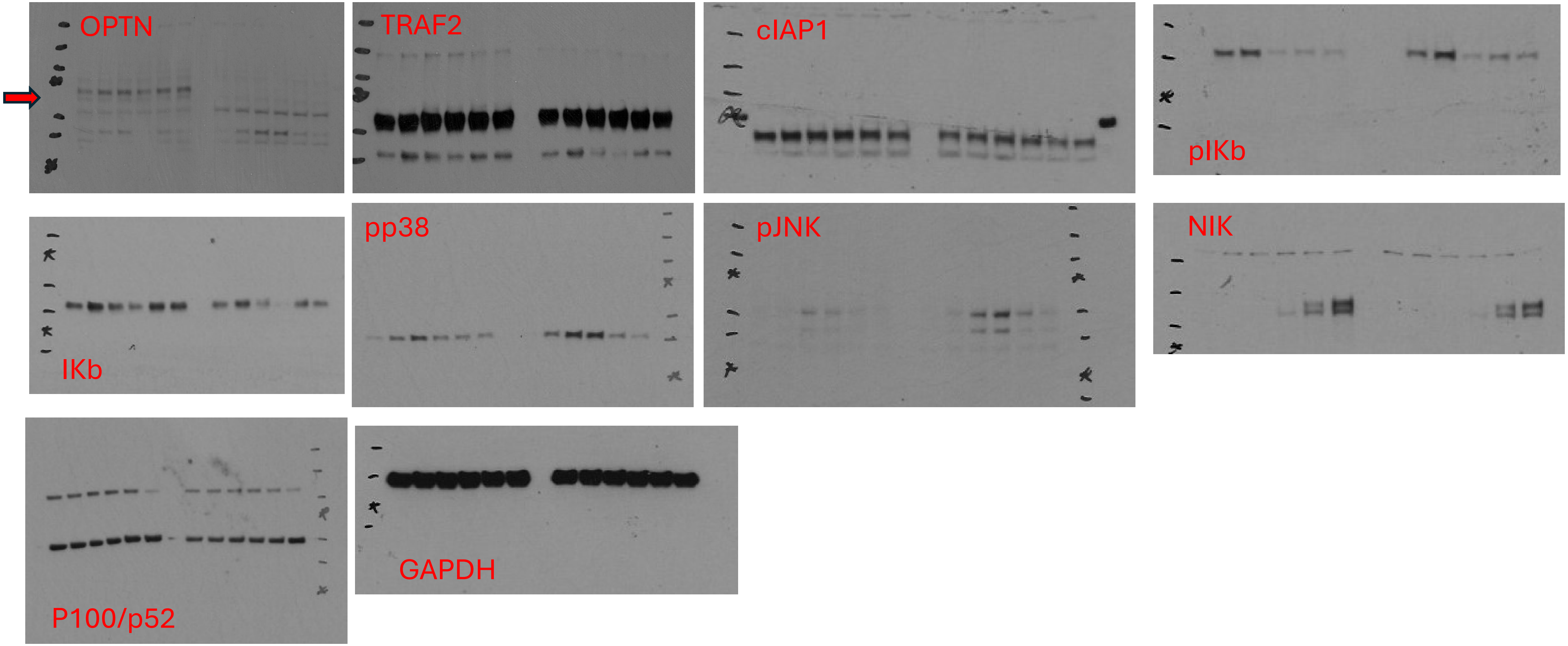

Figure 4C

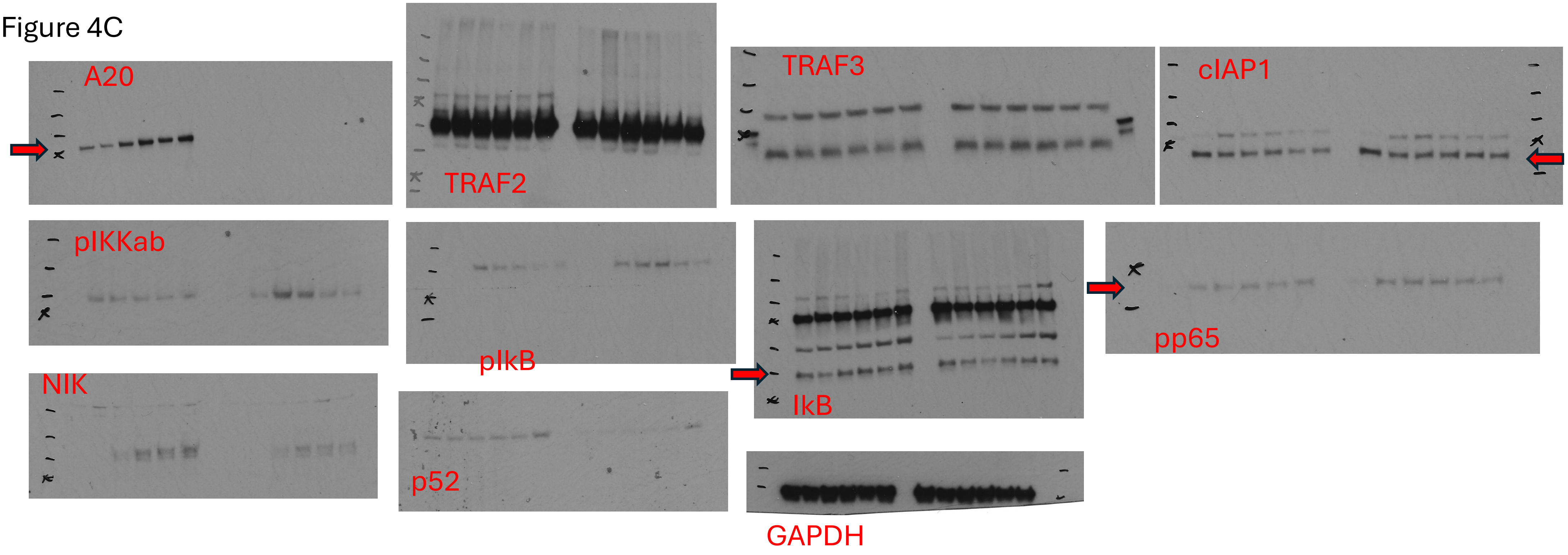

Figure 5A

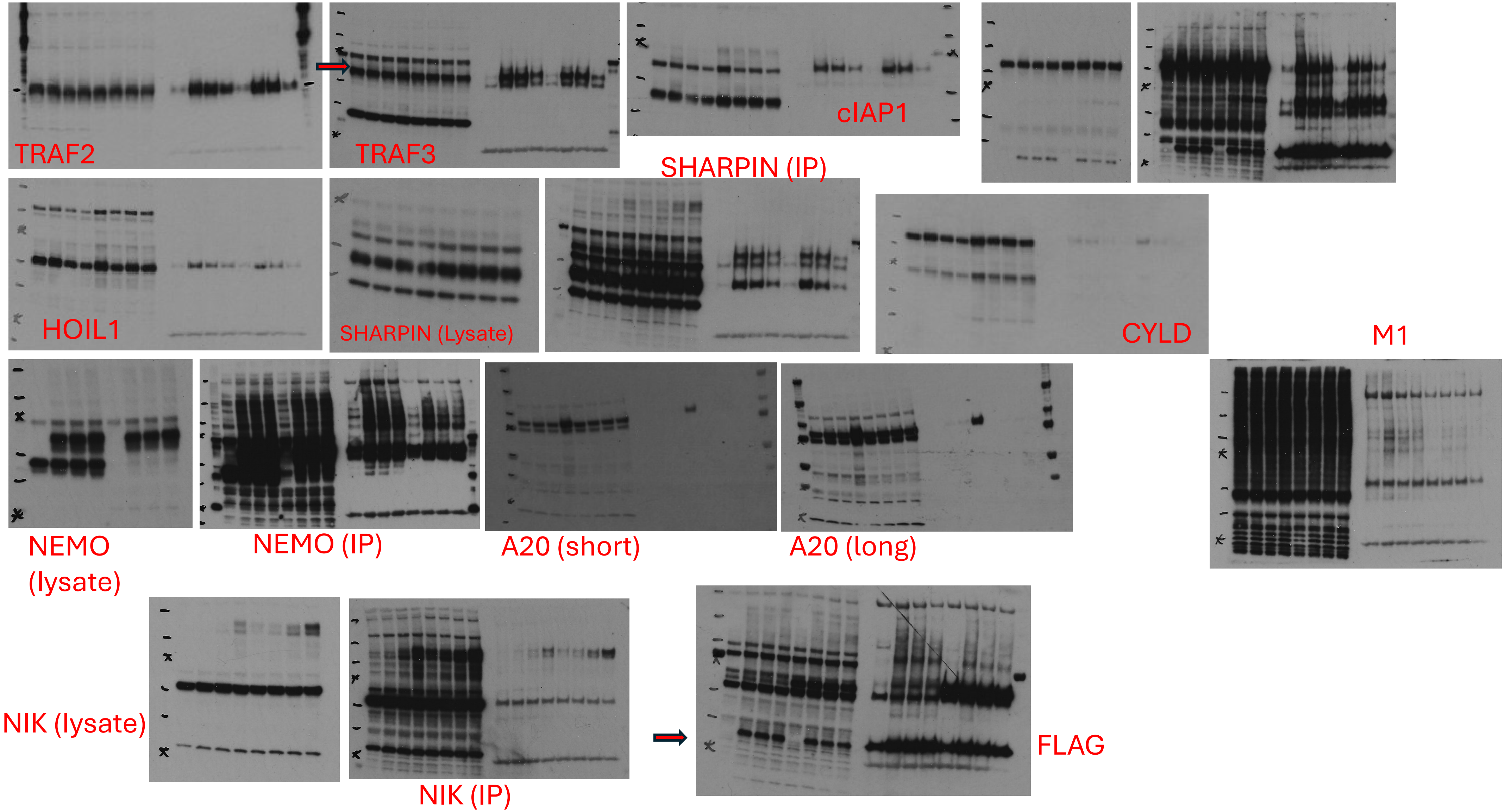
TRAF2
TRAF3
cIAP1
HOIP (Lysate)
HOIP (IP)
HOIL1
SHARPIN (Lysate)
SHARPIN (IP)
CYLD
M1
NEMO (lysate)
NEMO (IP)
A20 (short)
A20 (long)
NIK (lysate)
NIK (IP)
FLAG

Figure 5B

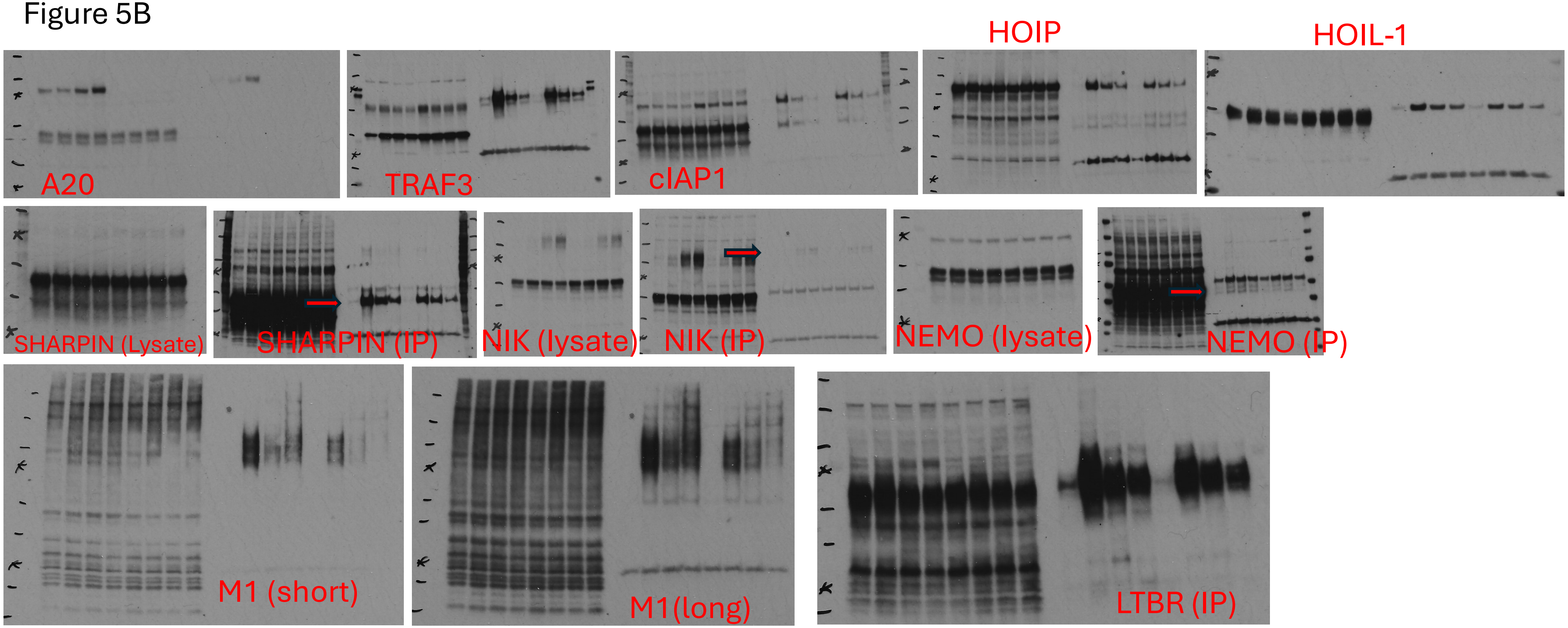
A20
TRAF3
cIAP1
HOIP
HOIL-1
SHARPIN (Lysate)
SHARPIN (IP)
NIK (lysate)
NIK (IP)
NEMO (lysate)
NEMO (IP)
M1 (short)
M1(long)
LTBR (IP)

Figure 5C

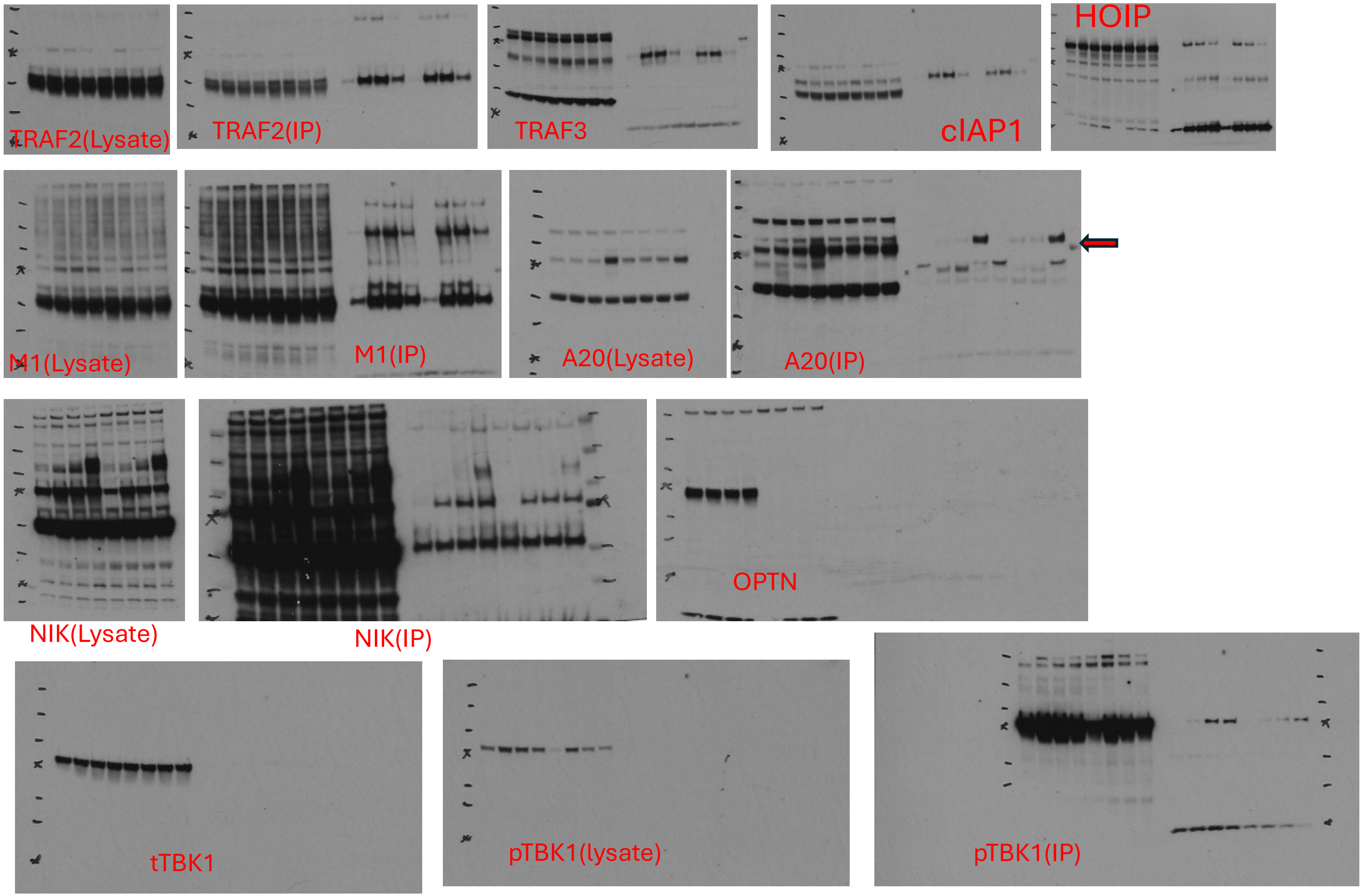
TRAF2(Lysate)
TRAF2(IP)
TRAF3
cIAP1
HOIP
M1(Lysate)
M1(IP)
A20(Lysate)
A20(IP)
NIK(Lysate)
NIK(IP)
OPTN
tTBK1
pTBK1(lysate)
pTBK1(IP)

Figure SF1

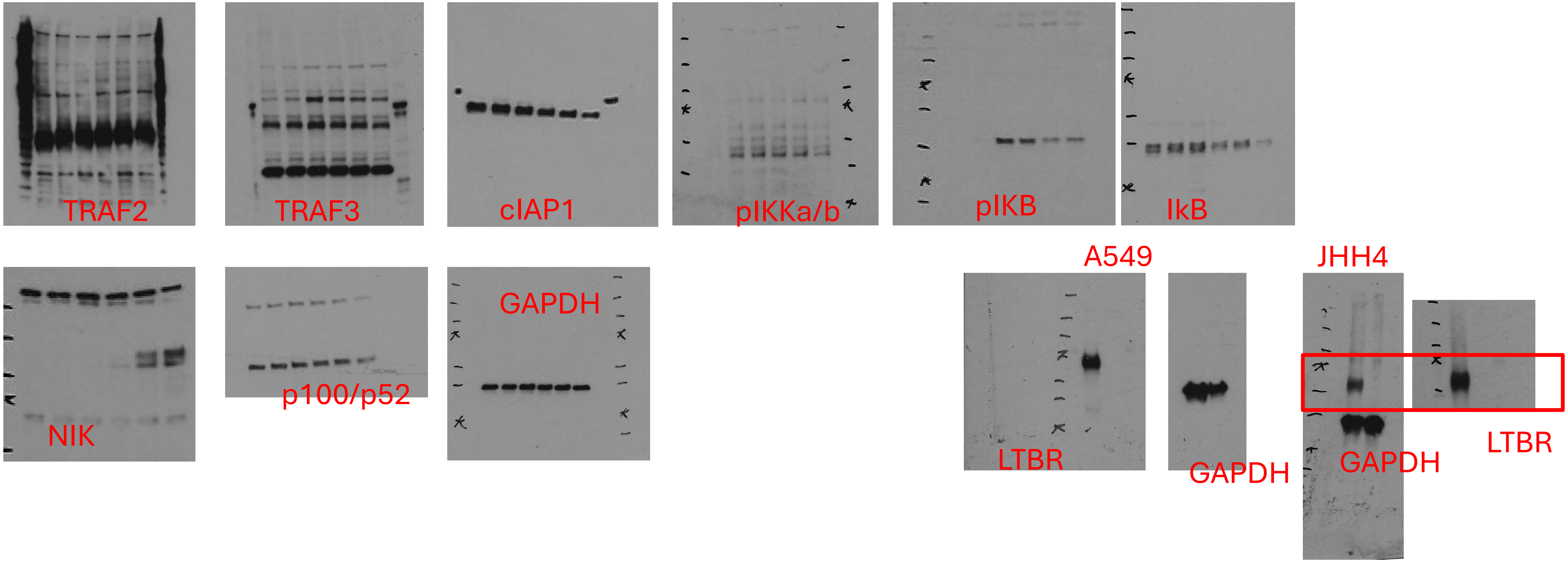

TRAF2
TRAF3
cIAP1
pIKKa/b
pIKB
IkB
NIK
p100/p52
GAPDH
A549
LTBR
GAPDH
JHH4
GAPDH
LTBR

Figure SF1B long

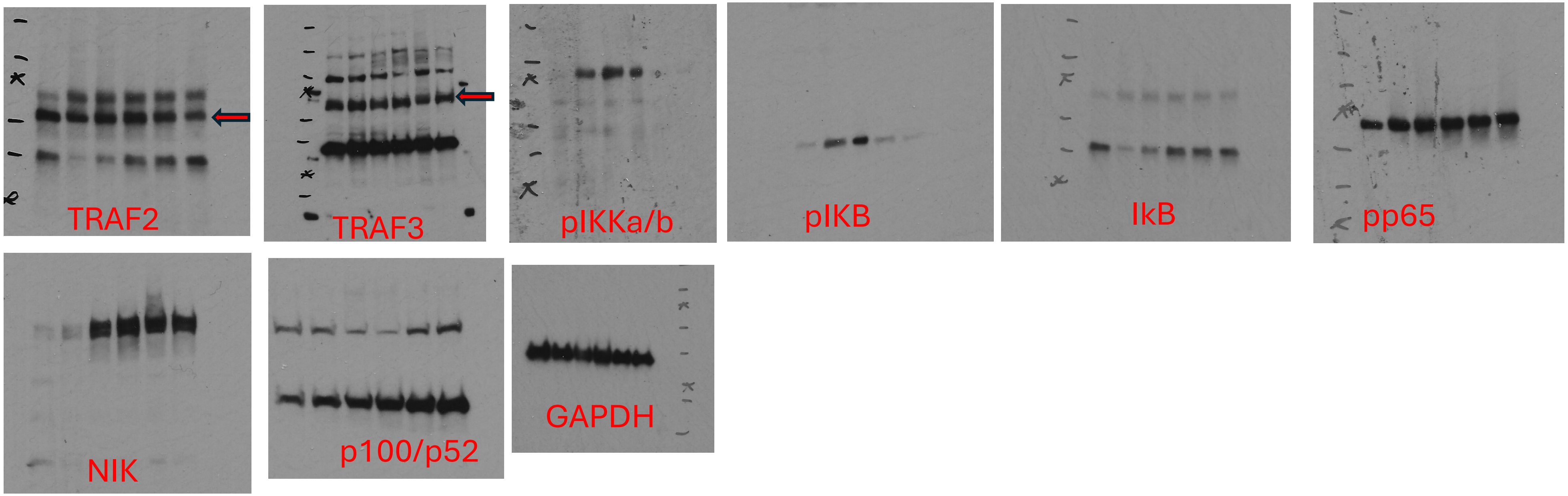

Figure SF3

* Ignore these two lines due to a repeated control

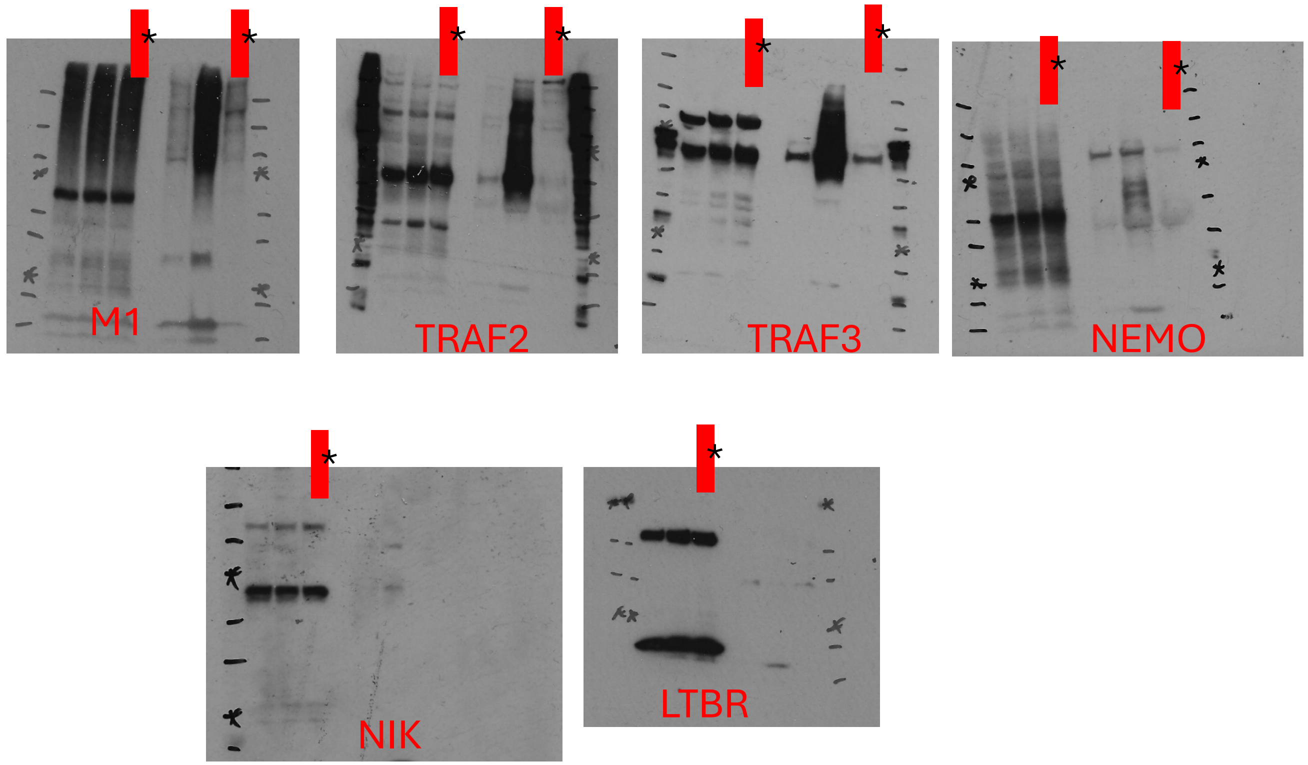

Figure SF4

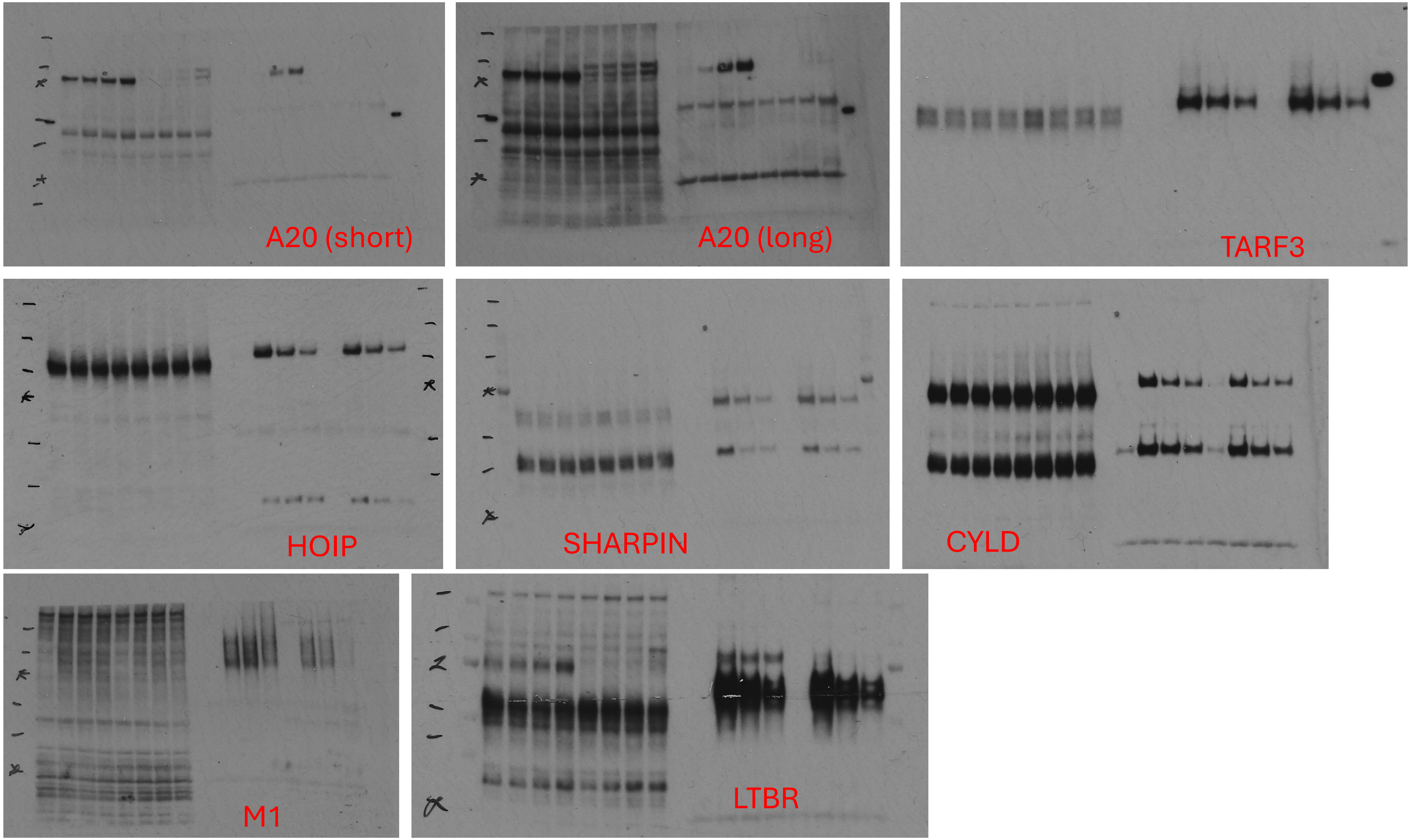

Figure SF5A

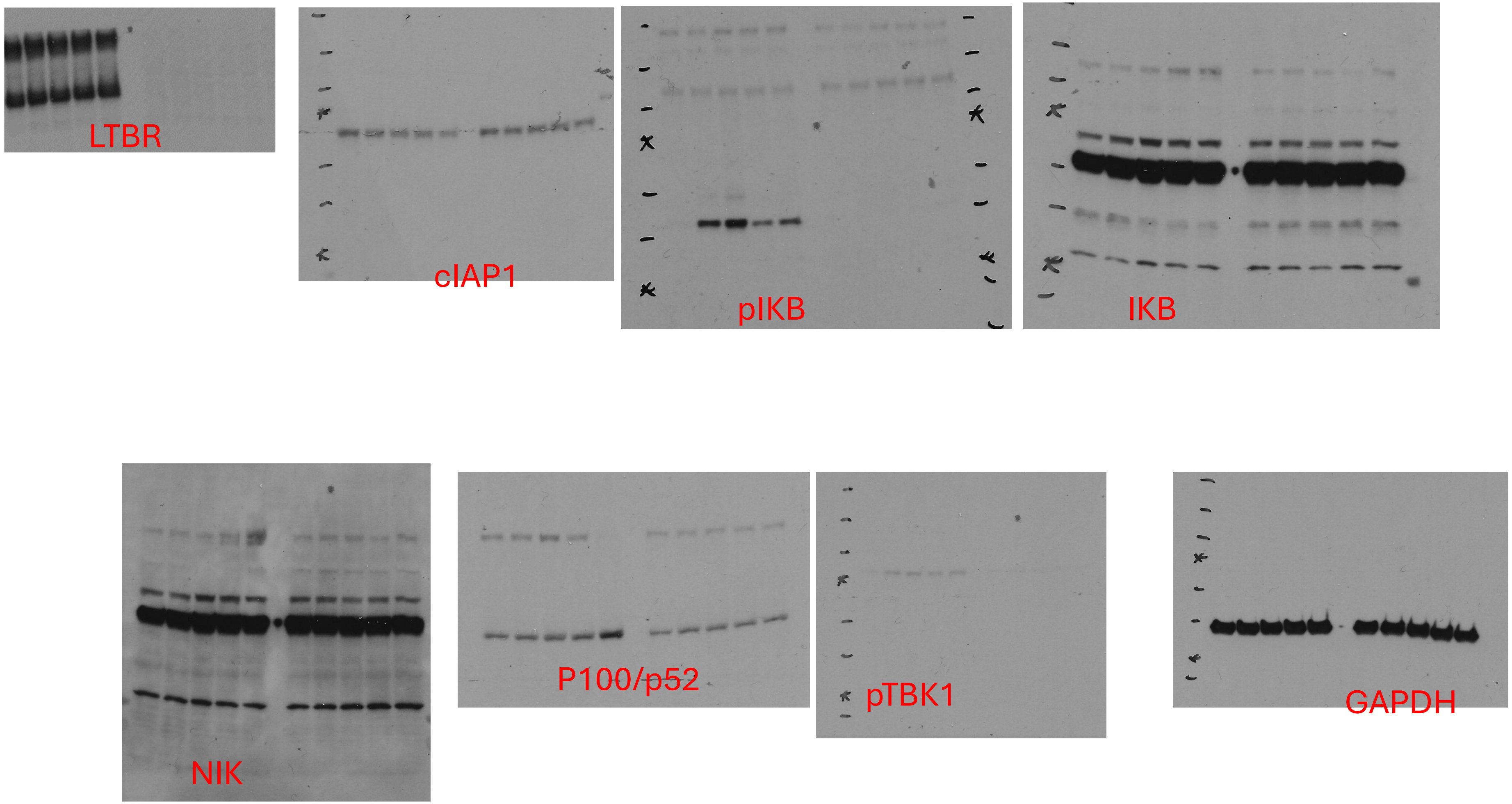

Figure SF5B

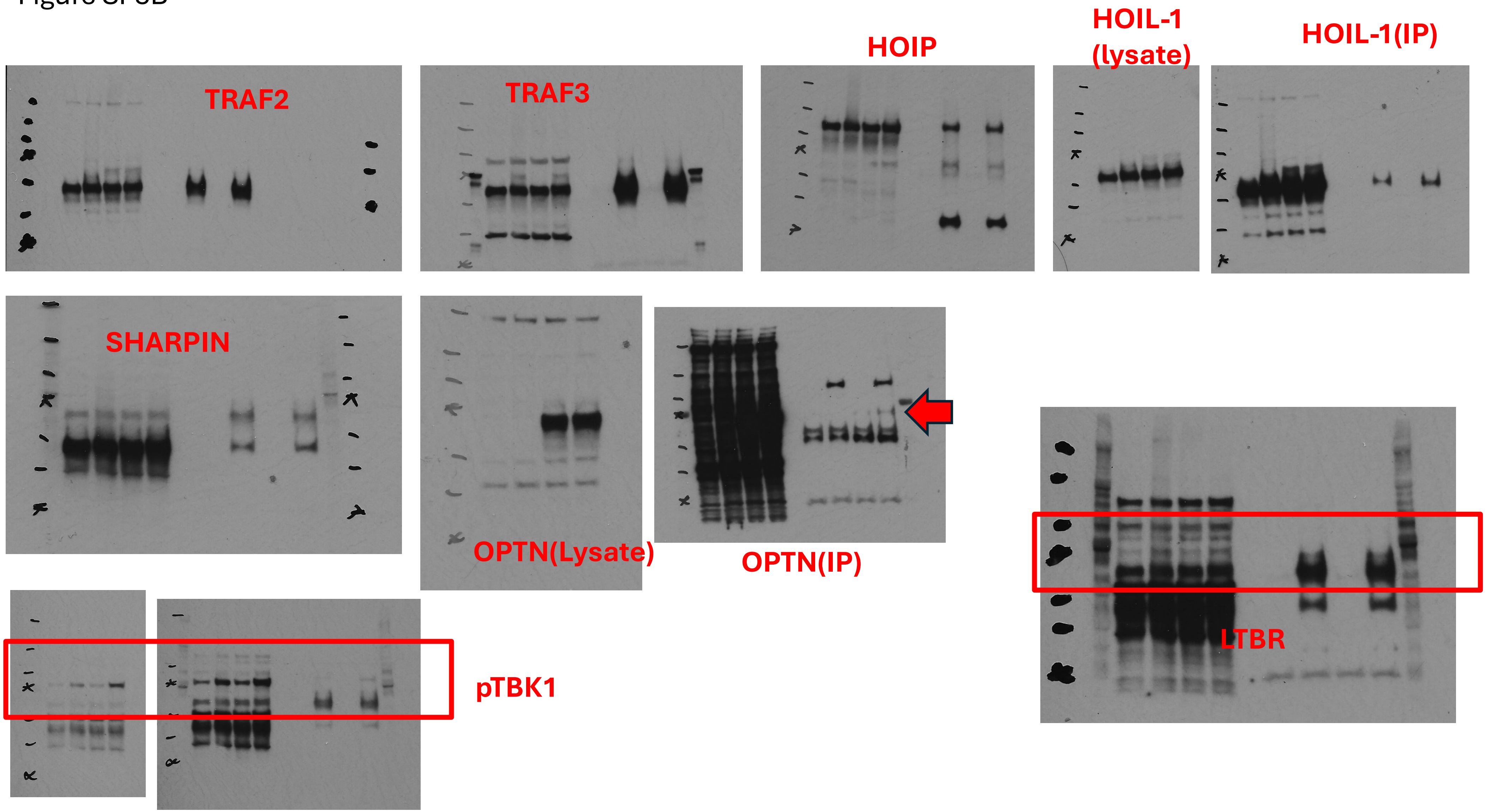
TRAF2
TRAF3
HOIP
HOIL-1 (lysate)
HOIL-1(IP)
SHARPIN
OPTN(Lysate)
OPTN(IP)
LTBR
pTBK1

Figure SF5C

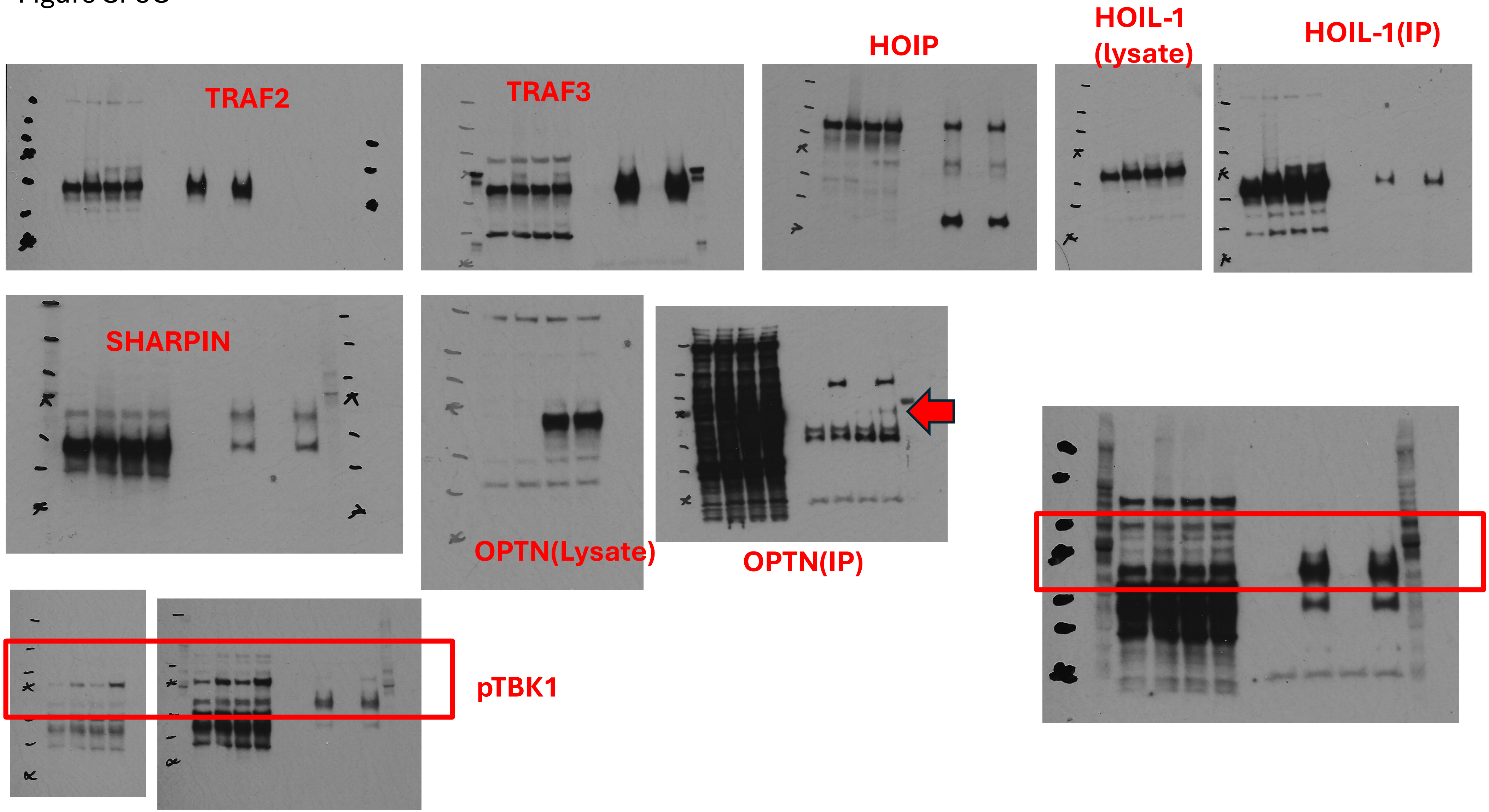

TRAF2
TRAF3
HOIP
HOIL-1 (lysate)
HOIL-1(IP)
SHARPIN
OPTN(Lysate)
OPTN(IP)
pTBK1

Figure SF5D

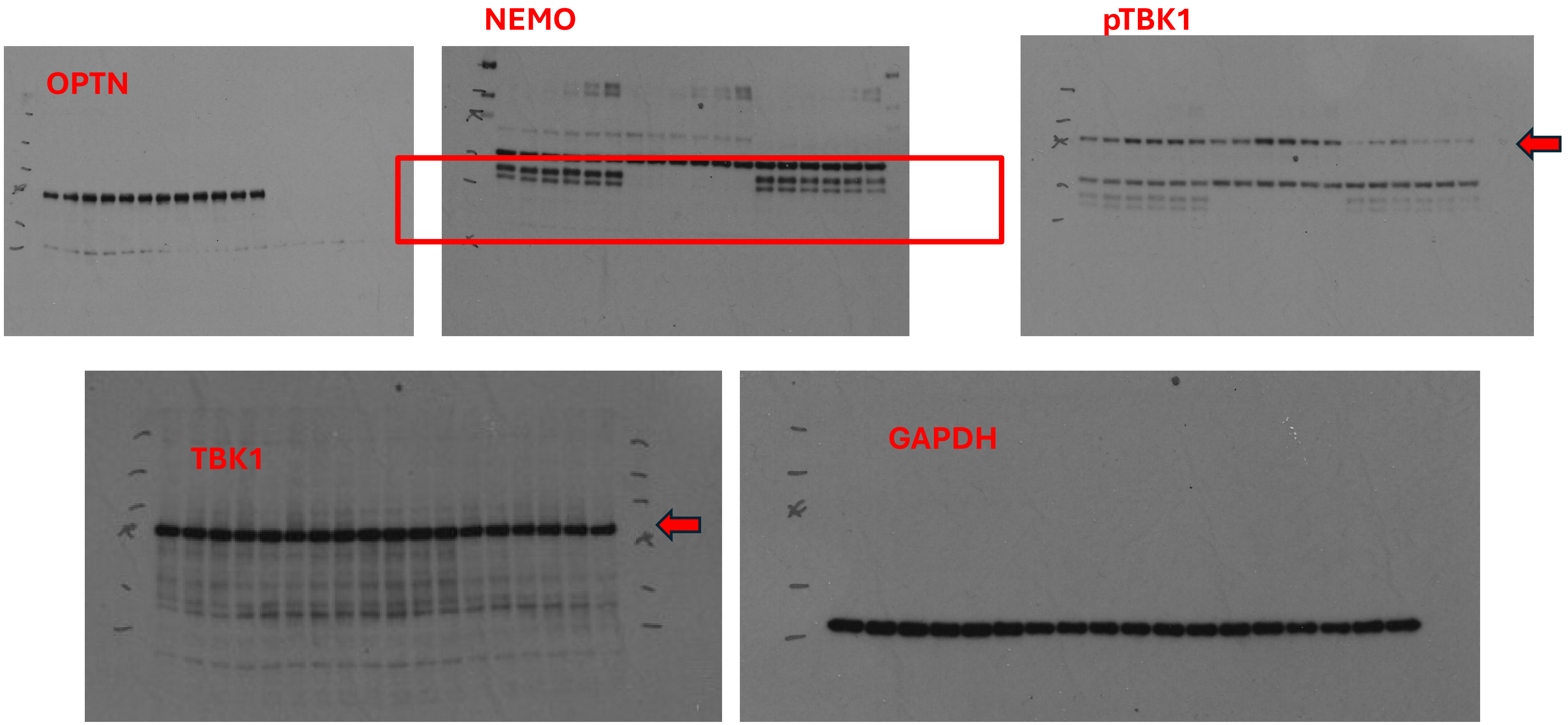

Figure SF5E

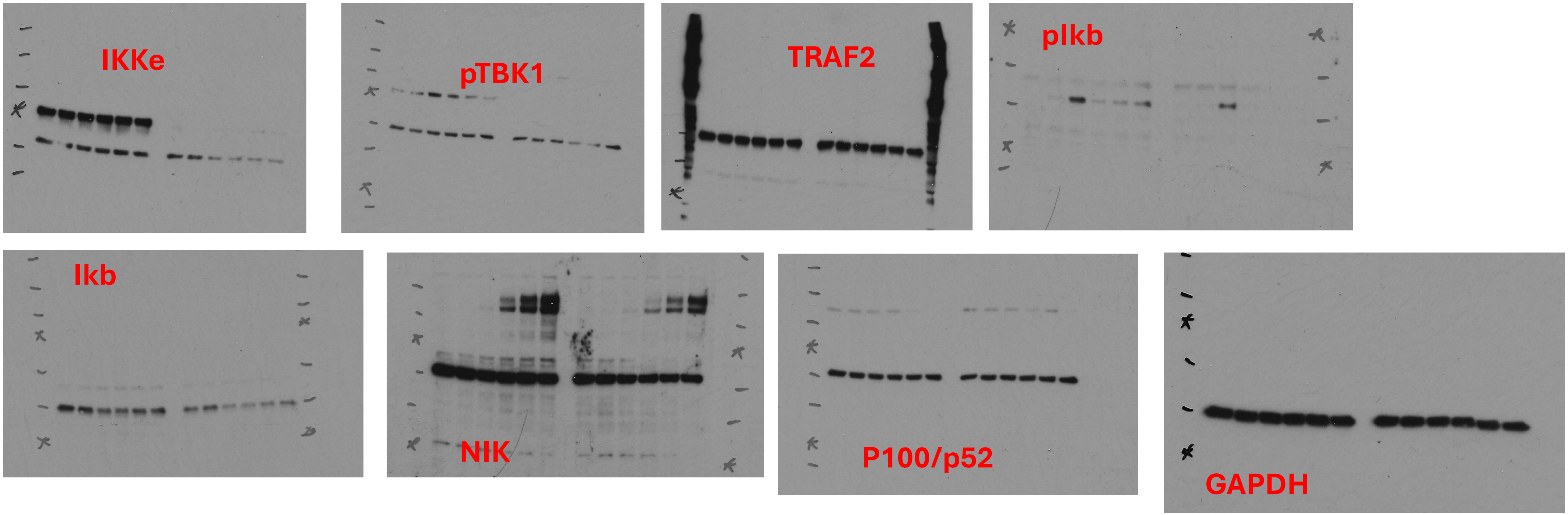

Supplement: Supplementary file 7 — Original uncropped blots [file 41418_2024_1355_MOESM7_ESM.pdf]
